# Novel Molecular Networks and Regulatory MicroRNAs in Type 2 Diabetes Mellitus: Multiomics Integration and Interactomics Study

**DOI:** 10.2196/32437

**Published:** 2022-02-23

**Authors:** Manoj Khokhar, Dipayan Roy, Sojit Tomo, Ashita Gadwal, Praveen Sharma, Purvi Purohit

**Affiliations:** 1 Department of Biochemistry All India Institute of Medical Sciences Jodhpur India

**Keywords:** type 2 diabetes mellitus, interactomics, integrative genomics, protein-protein interaction, microRNAs, miRNA, bioinformatics, multiomics, genomics, gene expression

## Abstract

**Background:**

Type 2 diabetes mellitus (T2DM) is a metabolic disorder with severe comorbidities. A multiomics approach can facilitate the identification of novel therapeutic targets and biomarkers with proper validation of potential microRNA (miRNA) interactions.

**Objective:**

The aim of this study was to identify significant differentially expressed common target genes in various tissues and their regulating miRNAs from publicly available Gene Expression Omnibus (GEO) data sets of patients with T2DM using in silico analysis.

**Methods:**

Using differentially expressed genes (DEGs) identified from 5 publicly available T2DM data sets, we performed functional enrichment, coexpression, and network analyses to identify pathways, protein-protein interactions, and miRNA-mRNA interactions involved in T2DM.

**Results:**

We extracted 2852, 8631, 5501, 3662, and 3753 DEGs from the expression profiles of GEO data sets GSE38642, GSE25724, GSE20966, GSE26887, and GSE23343, respectively. DEG analysis showed that 16 common genes were enriched in insulin secretion, endocrine resistance, and other T2DM-related pathways. Four DEGs, *MAML3, EEF1D, NRG1*, and *CDK5RAP2*, were important in the cluster network regulated by commonly targeted miRNAs (hsa-let-7b-5p, hsa-mir-155-5p, hsa-mir-124-3p, hsa-mir-1-3p), which are involved in the advanced glycation end products (AGE)-receptor for advanced glycation end products (RAGE) signaling pathway, culminating in diabetic complications and endocrine resistance.

**Conclusions:**

This study identified tissue-specific DEGs in T2DM, especially pertaining to the heart, liver, and pancreas. We identified a total of 16 common DEGs and the top four common targeting miRNAs (hsa-let-7b-5p, hsa-miR-124-3p, hsa-miR-1-3p, and has-miR-155-5p). The miRNAs identified are involved in regulating various pathways, including the phosphatidylinositol-3-kinase-protein kinase B, endocrine resistance, and AGE-RAGE signaling pathways.

## Introduction

Interactions among DNA, RNA, and proteins regulate their functions and have an immense effect on the underlying mechanistic processes in the pathophysiology of many diseases. Owing to the advent of newer technologies such as microarray and genome sequencing, it is now possible to investigate and analyze an enormous amount of genomic and proteomic data to predict disease pathology, outcome, and possible therapeutic targets [1]. Diabetes is a metabolic disorder characterized by hyperglycemia and glycosuria, which, if left untreated, leads to an array of complications and associated comorbidities [2]. These can include obesity, cardiomyopathy, nephropathy, retinopathy, neuropathy, and peripheral vascular disease, which have a lasting adverse effect on the quality of the patient’s life. To date, diabetes has affected almost half a billion individuals worldwide [3]. The absence of effective treatment strategies for this disease makes it a challenge to manage. The obligatory lifestyle changes and multiple treatment modalities, along with lifelong disease monitoring, depict an urgent and unmet need to develop newer and specific preventive and treatment strategies. Mortality rates in patients with type 2 diabetes mellitus (T2DM) are higher than those of individuals without diabetes and are linked to increased cardiovascular, renovascular, and neuropathic risks [4,5]. Thus, to reduce the morbidity and mortality associated with T2DM, it is important to gain a better understanding of its pathogenic pathways and regulation mechanisms. There is accumulating evidence that microRNAs (miRNAs) play an essential role in diabetes by reducing the expression of their various target genes [6,7]. It is also crucial to select the right target for disease treatment strategies in the early discovery phases, thus maximizing the drug’s success rates in the latter phases [8].

Currently, there is a vast amount of genomic data on diabetes and its complications. However, from its detection to the management of its late-stage complications, many areas still need to be explored and lacunae need to be filled. The role of molecular integration networks regulating the pathogenesis of T2DM in specific tissues is unknown. In this study, we have undertaken an in silico approach with existing tissue-specific microarray data of patients with diabetes to address this particular area by detecting novel diabetes-associated genes, their regulatory miRNAs, and their interactions to predict the pivotal pathways in tissues that are associated with disease onset and progression.

We selected five data sets from the Gene Expression Omnibus (GEO) database comprising the expression profiles of patients with diabetes and corresponding controls, and identified 16 differentially expressed genes (DEGs) overlapping the three preassigned groups. Gene Ontology (GO) and Kyoto Encyclopedia of Genes and Genomes (KEGG) pathway enrichment analyses were further used to classify the DEGs into cellular component (CC), biological process (BP), and molecular function (MF) classes. We selected four clusters from the protein-protein interaction (PPI) network and identified the seed genes. We further investigated the miRNA and hub gene network. Finally, we explored the 16 hub genes for biological pathway enrichment and their targeting miRNAs.

## Methods

### Data Collection

We searched several keywords, including “type 2 diabetes mellitus,” “tissue,” “pancreas,” “liver,” “heart,” “expression profiling by array,” and “*Homo sapiens*,” in the GEO data sets, among which five were selected for this study: GSE38642 [9-11], GSE25724 [12], GSE20966 [13], GSE26887 [14], and GSE23343 [15] (Table 1).

**Table 1:** Description of Gene Expression Omnibus data sets for three groups of organs.

Sample organ		T2DM^a^				Control				Platform		Country		Year
		Samples, n	Sex (M/F)	Mean age (years)	Samples, n		Sex (M/F)	Mean age (years)						
**Pancreas**														
	GSE25724 [12]	6	3/3	58.1	7		4/3	70.5	Affymetrix Human Genome U133A Array		Italy		2010	
	GSE20966 [13]	10	7/3	60.3	10		6/4	67.3	Affymetrix Human X3P Array		United States		2010	
	GSE38642 [9-11]	9	5/4	57.0	54		31/23	56.6	Affymetrix Human Gene 1.0 ST Array		Sweden		2012	
Heart (GSE26887) [14]		7	6/1	65.1	5		2/3	48.4	Affymetrix Human Gene 1.0 ST Array		Italy		2012	
Liver (GSE23343) [15]		10	4/6	—^b^	7		4/3	—	Affymetrix Human Genome U133 Plus 2.0 Array		Japan		2010	

^a^T2DM: Type 2 diabetes mellitus.

^b^Information not available.

### Inclusion and Exclusion Criteria

Data were restricted to human (*Homo sapiens*) samples, data set as the data type, expression profiling by array, tissue samples, and T2DM compared to controls (without diabetes). Thus, data from other organisms (*Mus musculus, Rattus norvegicus*, *Xenopus laevis*); series data; expression profiling by other methods (eg, massively parallel signature sequencing, reverse transcription-polymerase chain reaction, serial analysis of gene expression, genome variation or occupancy profiling by single-nucleotide polymorphism array, genome tiling array); nontissue samples (eg, blood, serum, semen, saliva, urine, body fluid); and data from patients with type 1 diabetes, gestational diabetes, or prediabetes were excluded.

**Figure 1:**
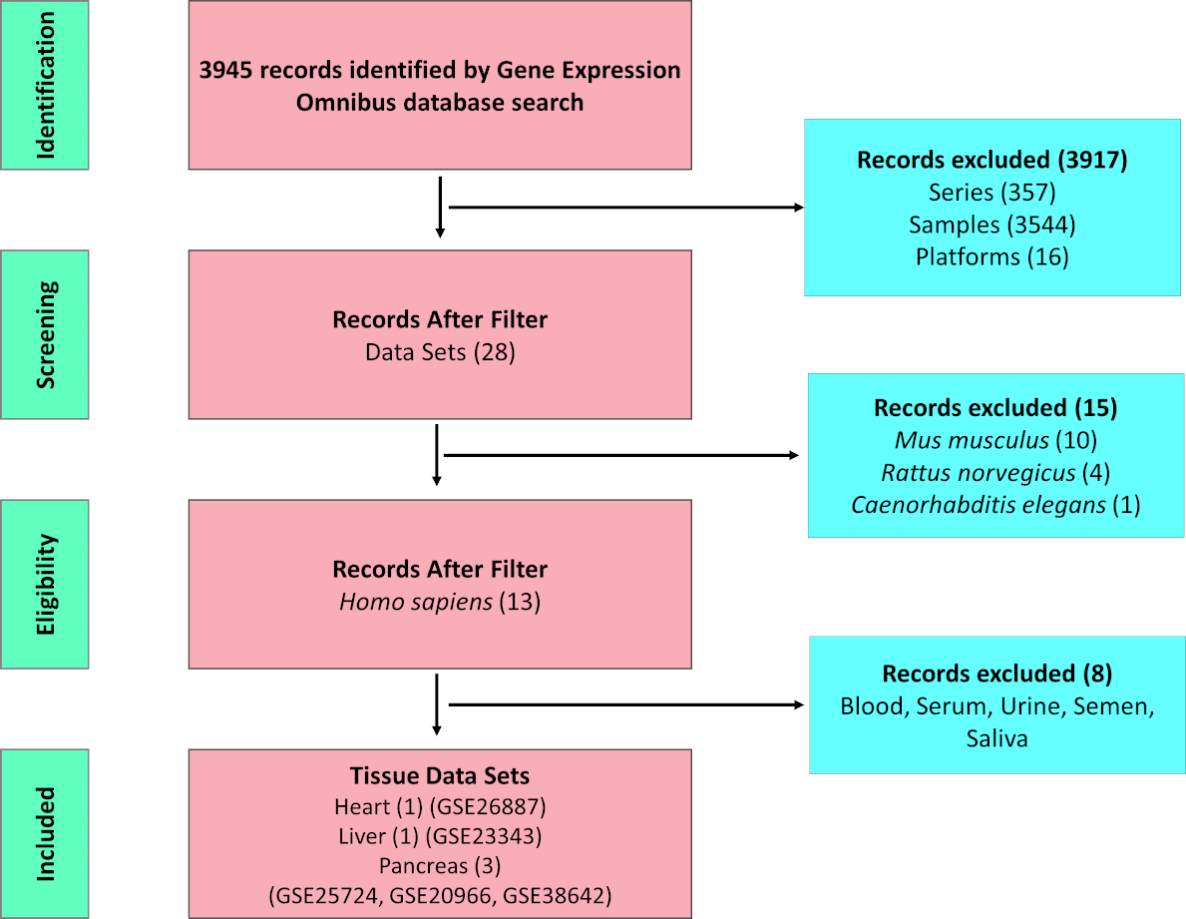
Flow diagram illustrating the process of data collection and number of data sets considered for inclusion.

The data collection process is summarized in Figure 1.

### Identification and Assortment of Differentially Expressed mRNAs

The DEGs were obtained from the five data sets using the online interactive tool GEO2R [16]. The cutoff for the selection was kept at the default of *P*<.05. The relaxed *P*-value cutoff was fixed for the initial selection because (1) we were subjecting the selected genes for a repeated analysis using ImaGEO software with a cutoff adjusted *P*<.05, and (2) the application of a stringent *P*-value cutoff in the initial selection did not enable obtaining an adequate number of genes from each data set for undertaking a meta-analysis. The overlapping DEGs among the three data sets of pancreatic tissues from patients with T2DM and controls (GSE38642, GSE25724, and GSE20966) were identified using the Venn diagram tool [17,18]. Subsequently, the common DEGs of these three data sets (GSE38642, GSE25724, and GSE20966) with those of the other two data sets for heart (GSE26887) and liver (GSE23343) samples were identified separately. The fold change expression distribution was visualized by heat maps and violin plots using the R *limma* (linear models for microarray data) package and Orange Data Mining software [19,20].

**Figure 2:**
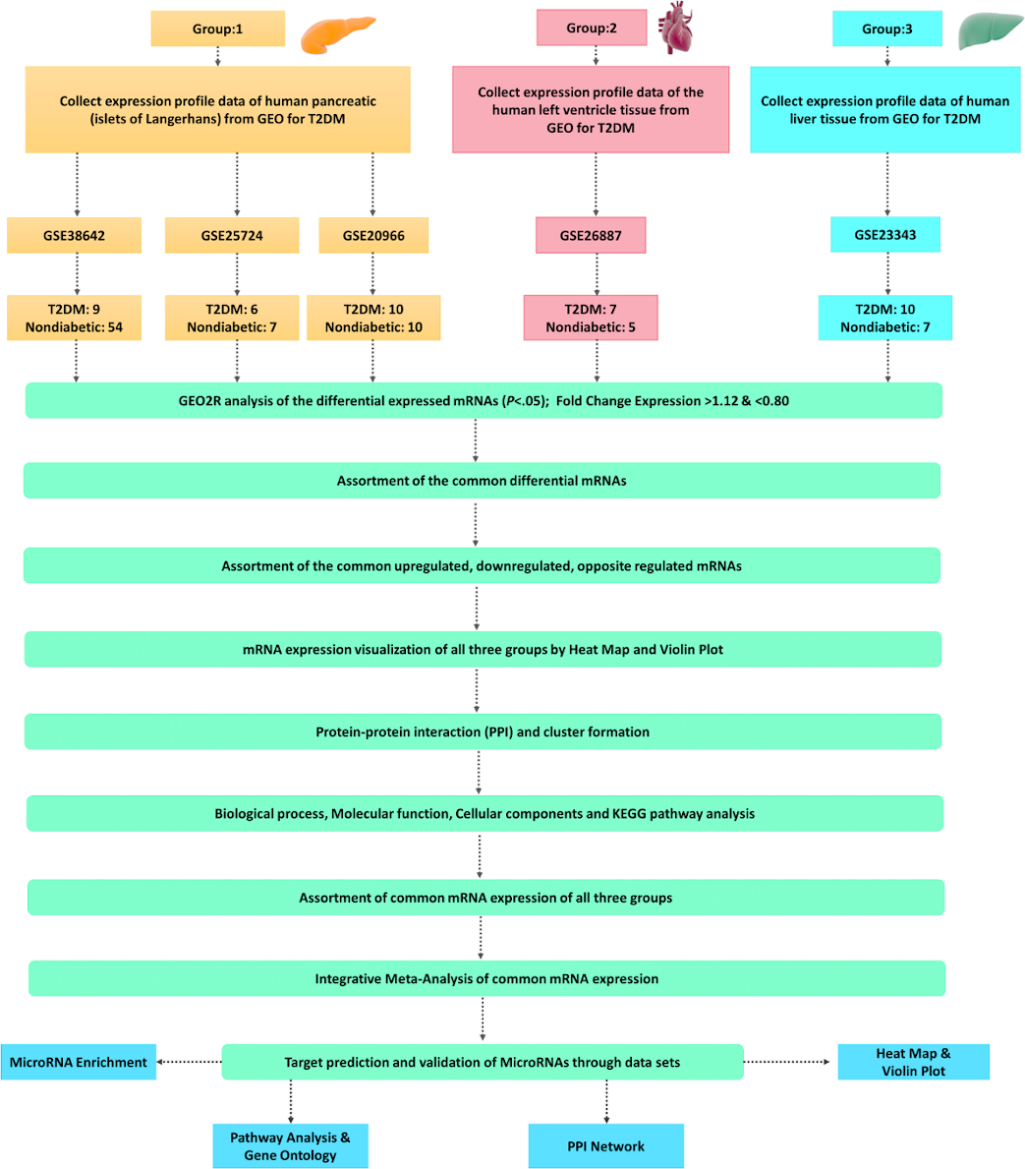
Flowchart of data processing and analysis. GEO: Gene Expression Omnibus; KEGG: Kyoto Encyclopedia of Genes and Genomes; T2DM: type 2 diabetes mellitus.

To check the quality of the data, quality control plots were assessed in the form of volcano plots, mean difference plots, and mean-variance trends. A volcano plot visualizes the DEGs by plotting the statistical significance against the magnitude of change, whereas the mean difference plot displays the log2 fold change against the average log2 expression level. The mean-variance trend, generated using the R packages plotSA and vooma, assesses the variance of the data. The workflow for the data processing and analysis is portrayed in Figure 2.

### Functional Enrichment and KEGG Pathway Analysis

The DEGs were divided into three groups according to the tissue (Figure 2). The functional enrichment of each group related to T2DM was analyzed with the Database for Annotation, Visualization and Integrated Discovery (DAVID) tool for significant MF, CC, and BP GO terms. KEGG pathway analysis was performed with piNET, a versatile tool that integrates protein signatures with transcriptomic and proteomic signatures [21-23]. DAVID includes an analysis of KEGG pathways and enrichment significance of GO terms from the three categories (MF, CC, BP). We defined *P*<.05 as significantly enriched. The nonsignificant findings were manually removed.

### PPI Network Construction and Identification of Hub Genes

The DEGs in the three groups were used to construct the PPI network using Search Tool for the Retrieval of Interacting Genes/Protein (STRING) [24]. We established the PPI network using only the overlapping DEGs with greater than 0.4 confidence score cutoffs. The “combined scores” were computed by integrating the probabilities from the various different types of evidence (by evidence channels), while correcting for the probability of randomly observing an interaction [25]. The number of interactions (by confidence level) were divided into four groups: (1) highest confidence (score≥0.90), (2) high confidence or better (score≥0.70), (3) medium confidence or better (score≥0.40), (4) low confidence links (score≥0.15). We chose medium confidence as the default setting given in STRING.

The interaction networks for each group were constructed by Cytoscape [26,27]. The Molecular Complex Detection (MCODE) [28] plugin of Cytoscape was employed to visualize significant genes in all three groups with a degree cutoff=2, node score cutoff=0.2, k-score=2, and maximum depth=100. The criteria for selecting the top 3 clusters were set as MCODE score≥3 and number of nodes≥3.

### Integrative Gene Expression Meta-analysis

ImaGEO is a web tool for gene expression meta-analysis that was used to perform a comprehensive meta-analysis from all five data sets. For the retrieval and preprocessing of the data, the GEOquery package in R was used, followed by quality control, gene filtering expression, meta-analysis, and functional analysis. The meta-analysis was based on the functional modules with the MetaDE R package. For this study, we used the “effect size” parameter estimation with a fixed-effects model and an adjusted *P* value threshold of .05. The allowable missing values was kept at the default of 10%.

### Target Prediction, Validation, and miRNA–Hub Gene Interaction

The top 10 targeting miRNAs of the hub genes were predicted by the well-established miRNA target prediction database miRNet 2.0 [29] with *H. sapiens* (human) as the selected organism. Default values for the degree of interaction and betweenness were retained. Common miRNAs and targeted mRNAs of all groups were sorted by the Venn diagram tool [30]. The network of all targeting miRNAs and the coexpressed mRNAs was created with FunRich and Cytoscape software. To validate the targeting miRNAs, we further sorted miRNA data sets in T2DM for comparison of differentially expressed miRNAs.

### Functional Enrichment and KEGG Pathway Analysis for MiRNAs

All common miRNAs were enriched by MicroRNA Enrichment Turned Network (MIENTURNET) and KEGG pathway analysis [31]. MIENTURNET is a web tool based on the shiny package in R studio for both statistical and network-based analyses of miRNA-target enrichment. Functional enrichment was retrieved for the input list of genes, with the minimum interaction threshold set at 2 and an adjusted *P* value of .05. The input list infers possible experimental or computational evidence of miRNA-based regulation.

## Results

### Identification of DEGs in all Combined Groups

**Figure 3:**
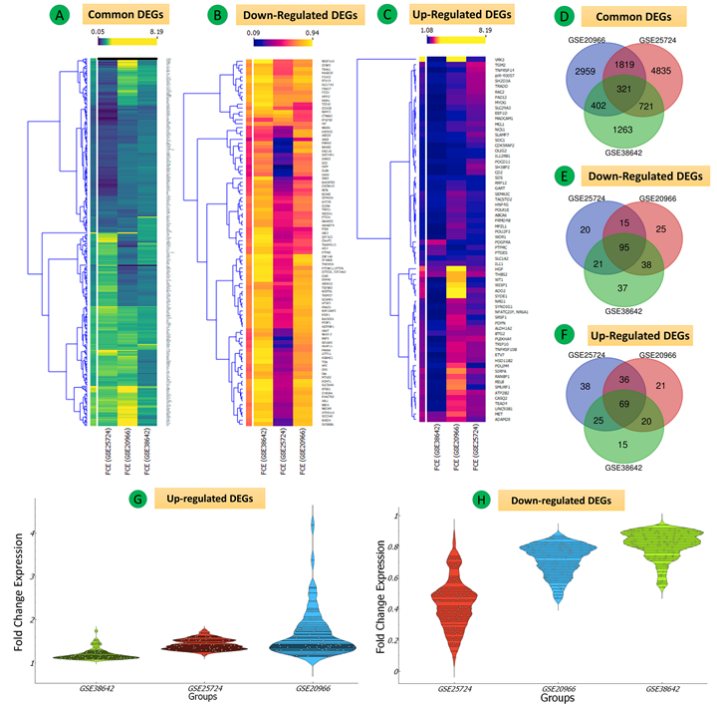
Differential mRNA expression of all three data sets (GSE38642, GSE25724, GSE20966) for Group 1 (pancreas tissues) in type 2 diabetes mellitus. (A-C) Heat maps of all, downregulated and upregulated differentially expressed genes (DEGs). Fold change expression (FCE) levels are displayed in ascending order from blue to yellow. (D-F) Venn diagrams of the total downregulated and upregulated DEGs of the three data sets. (G, H) Violin plots showing the entire FCE distribution of all three data sets for upregulated and downregulated common DEGs.

**Figure 4:**
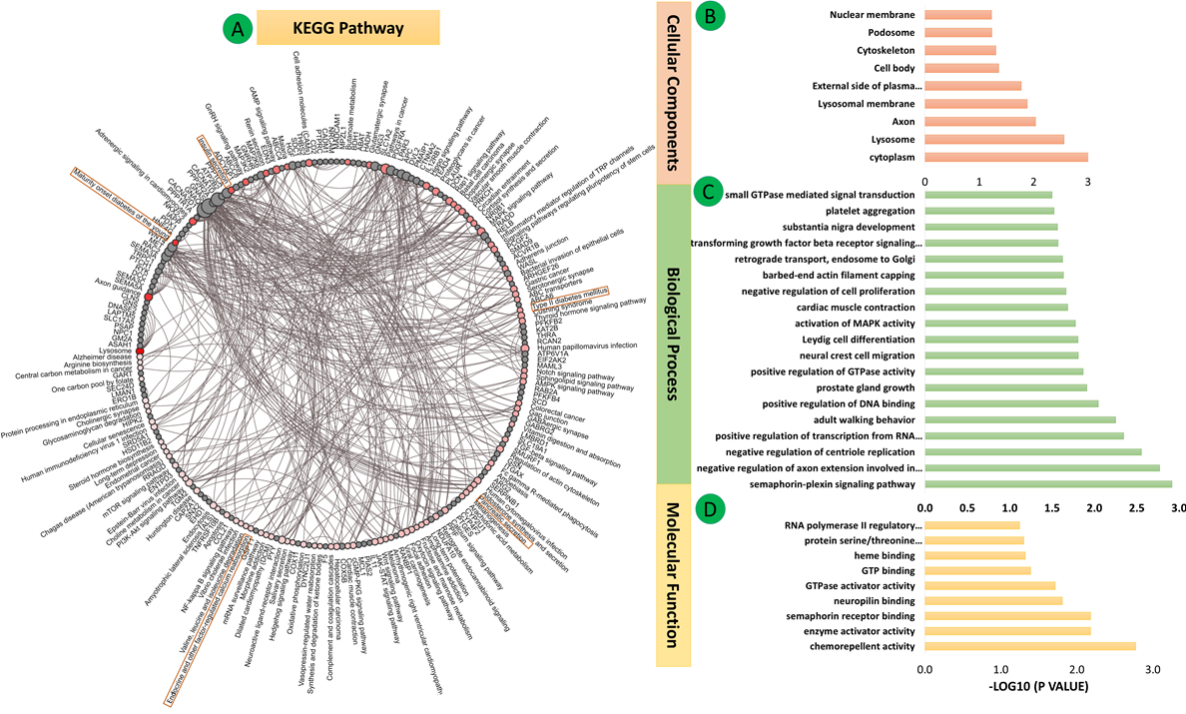
Differential mRNA expression of all three data sets (GSE38642, GSE25724, GSE20966) for Group 1 (pancreas tissues) in type 2 diabetes mellitus. (A-D) Enrichment analysis of common DEGs. (A) Kyoto Enclyclopedia of Genes and Genomes (KEGG) pathway enrichment: the connections are shown using red nodes (pathways) or brown nodes (DEGs) through the brown edges in a circle. The larger the size of the grey node, the more connected it is within the network. The density of red color indicates the number of connecting DEGs. (B) Gene Ontology cellular component terms. (C) Gene Ontology biological process terms. (D) Gene Ontology molecular function terms. Significant pathways represent adjusted *P*<.05 (false discovery rate).

**Figure 5:**
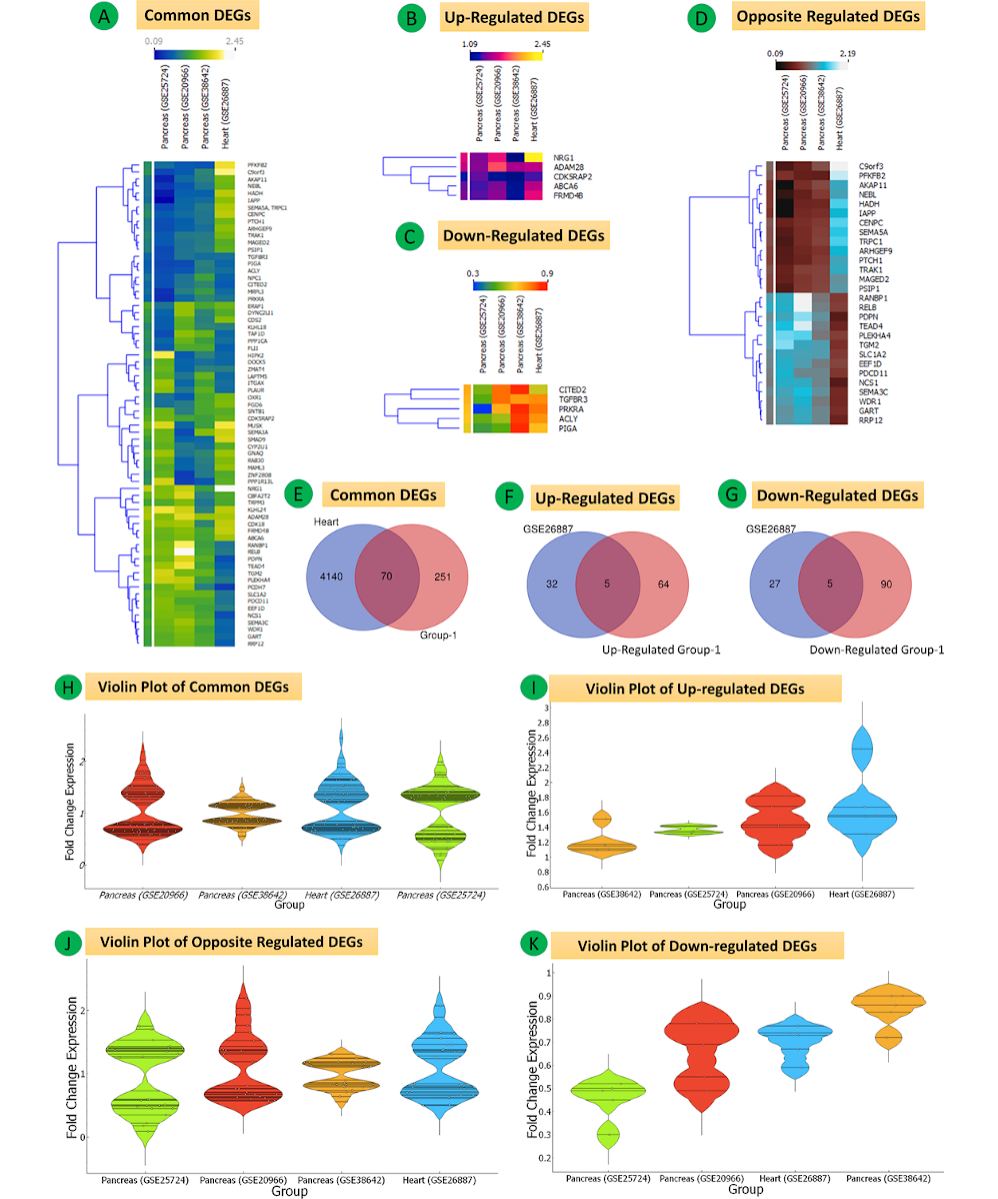
(A-D) Heat maps of mRNA expression for the three data sets (GSE38642, GSE25724, GSE20966) of Group 1 (pancreas) and the GSE26887 (heart) data set showing all differentially expressed genes (DEGs), DEGs regulated in opposite directions, upregulated DEGs, and downregulated DEGs. (E-G) Venn diagrams of complete, upregulated, and downregulated common DEGs. The upper part of the heat map shows fold change expression (FCE) values represented by varying color densities. (H-K) Violin plots showing the entire FCE distribution of all four data sets of Group 1 (pancreas) and the heart data set.

**Figure 6:**
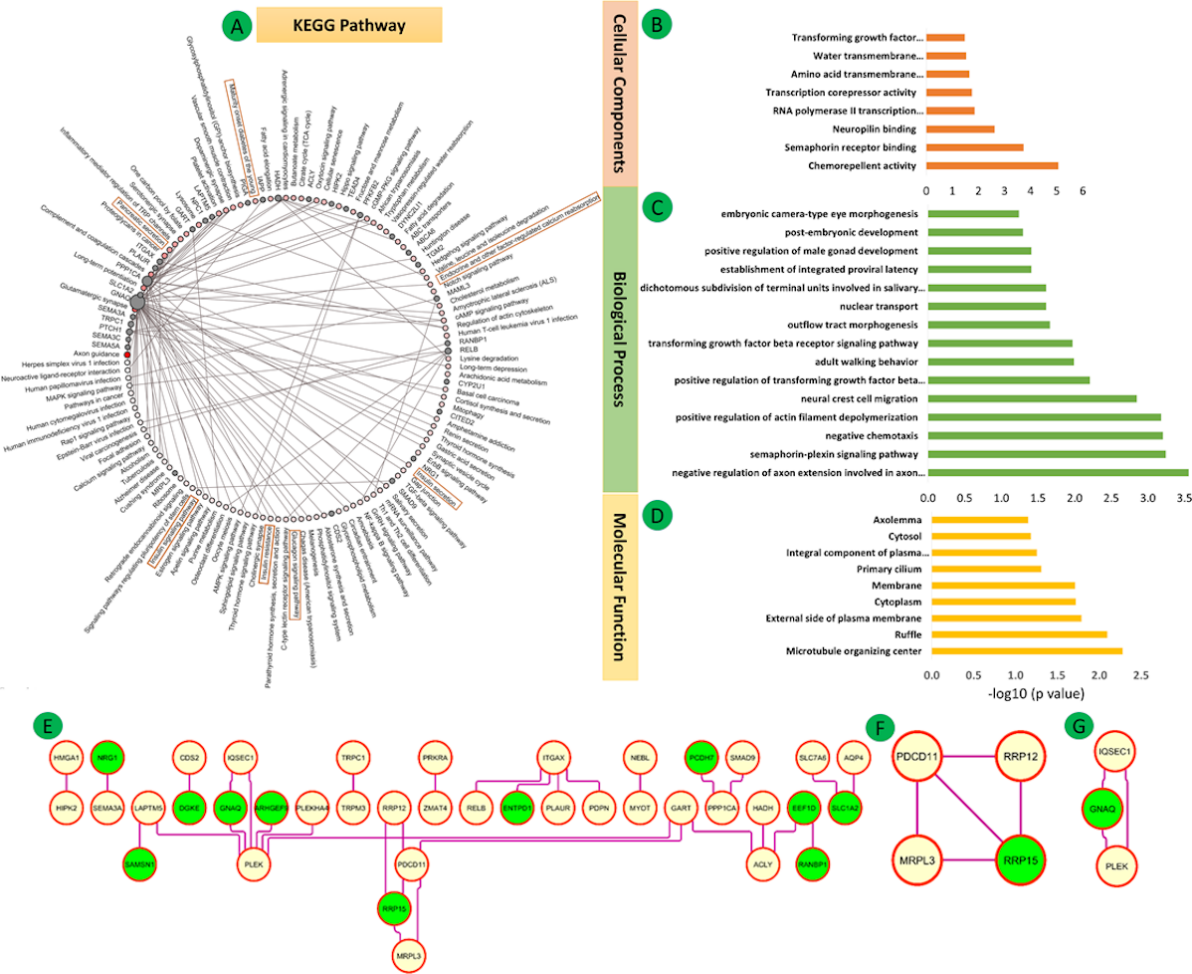
(A) Enriched Kyoto Encyclopedia of Genes and Genomes (KEGG) pathways. The connections are shown using red nodes (pathways) or brown nodes (DEGs) through the brown edges in a circle. The larger the size of the grey node, the more connected it is within the network. The density of red color indicates the number of connecting DEGs. (B-D) Gene Ontology enrichment for (B) cellular component, (C) biological process, and (D) molecular function terms. Significant pathways represent adjusted *P*<.05 (false discovery rate). (E) Top hub genes in the network. (F, G) Clusters using MCODE.

**Figure 7:**
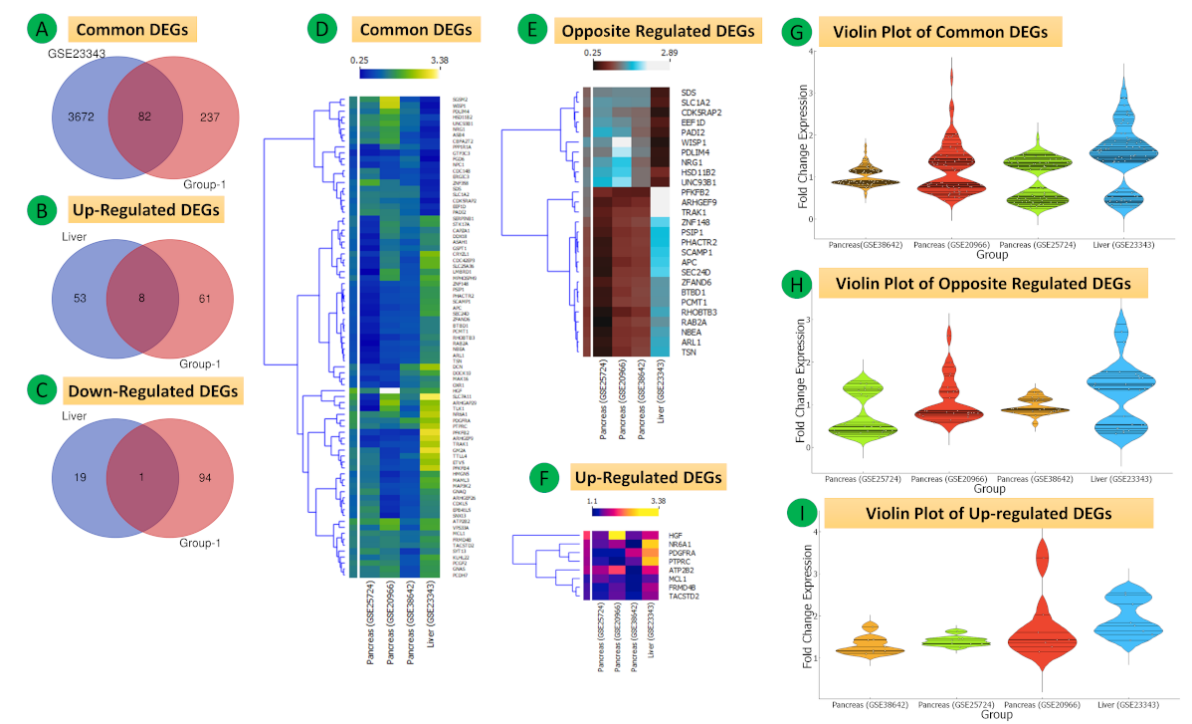
mRNA expression of three data sets (GSE38642, GSE25724, and GSE20966) of Group 1 (pancreas) and the GSE23343 data set (liver). (A-C) Venn diagrams of complete, upregulated, and downregulated common differentially expressed genes (DEGs). (D-F) Heat maps of common, oppositely regulated, and common upregulated DEGs. The upper part of the heat map shows the fold change in expression values reflected by respective color densities. (G-I) Violin plots showing the entire fold change expression (FCE) distribution of all four data sets for complete common DEGs, oppositely regulated DEGs, and common upregulated DEGs.

**Figure 8:**
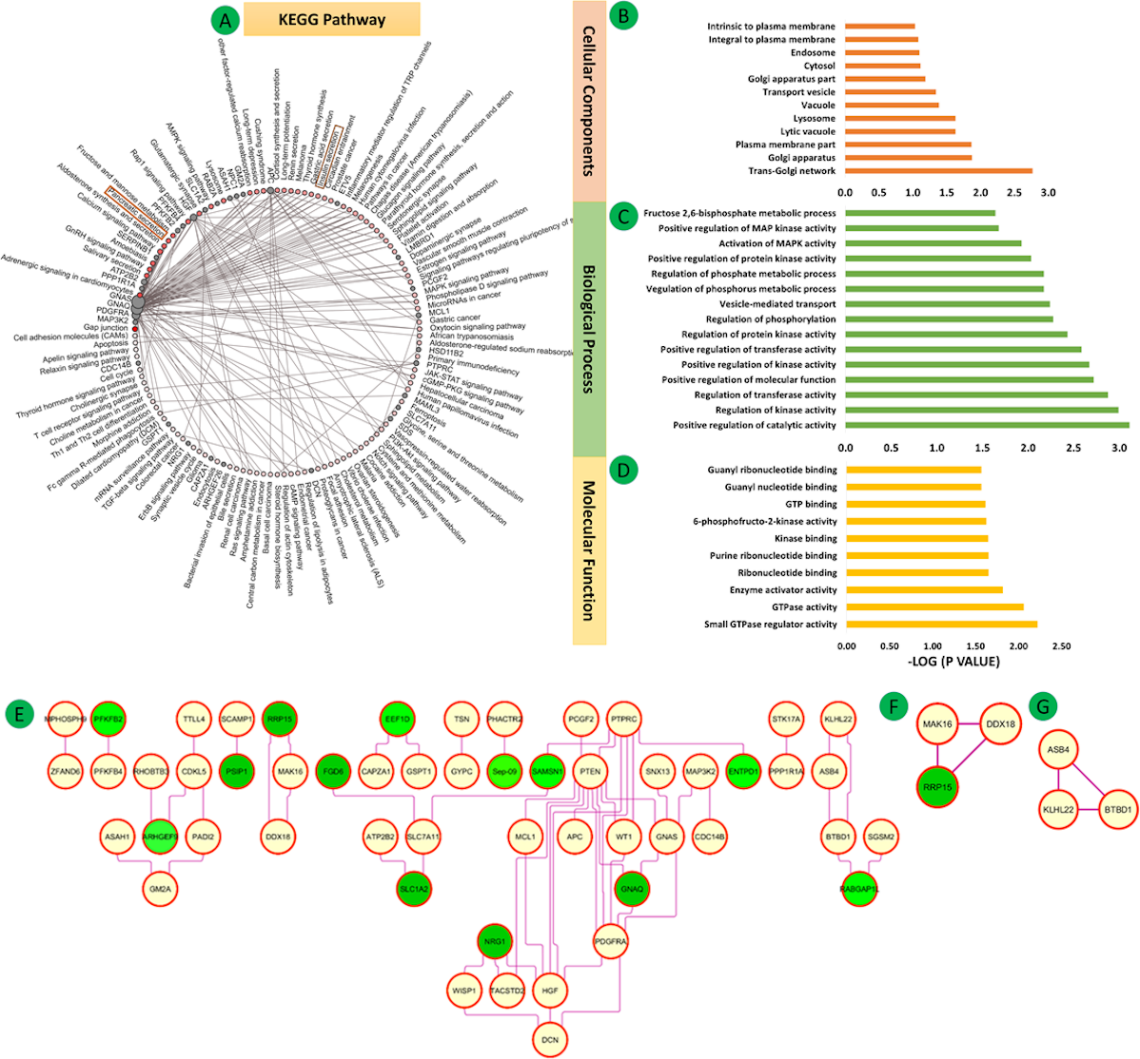
(A-D) Kyoto Encyclopedia of Genes and Genomes (KEGG) pathway and Gene Ontology functional enrichment analysis of common DEGs. The connections are shown using red nodes (pathways) or brown nodes (DEGs) through the brown edges in a circle. The larger the size of the grey node, the more connected it is within the network. The density of red color indicates the number of connecting DEGs. (E) Protein-protein interaction networks of 82 overlapping DEGs of GSE23343 and co-expressed genes of Group 1 (321 DEGs) composed of 82 nodes and 56 edges. (F, G) Clusters from the network. Significant pathways represent adjusted *P*<.05 (false discovery rate).

**Figure 9:**
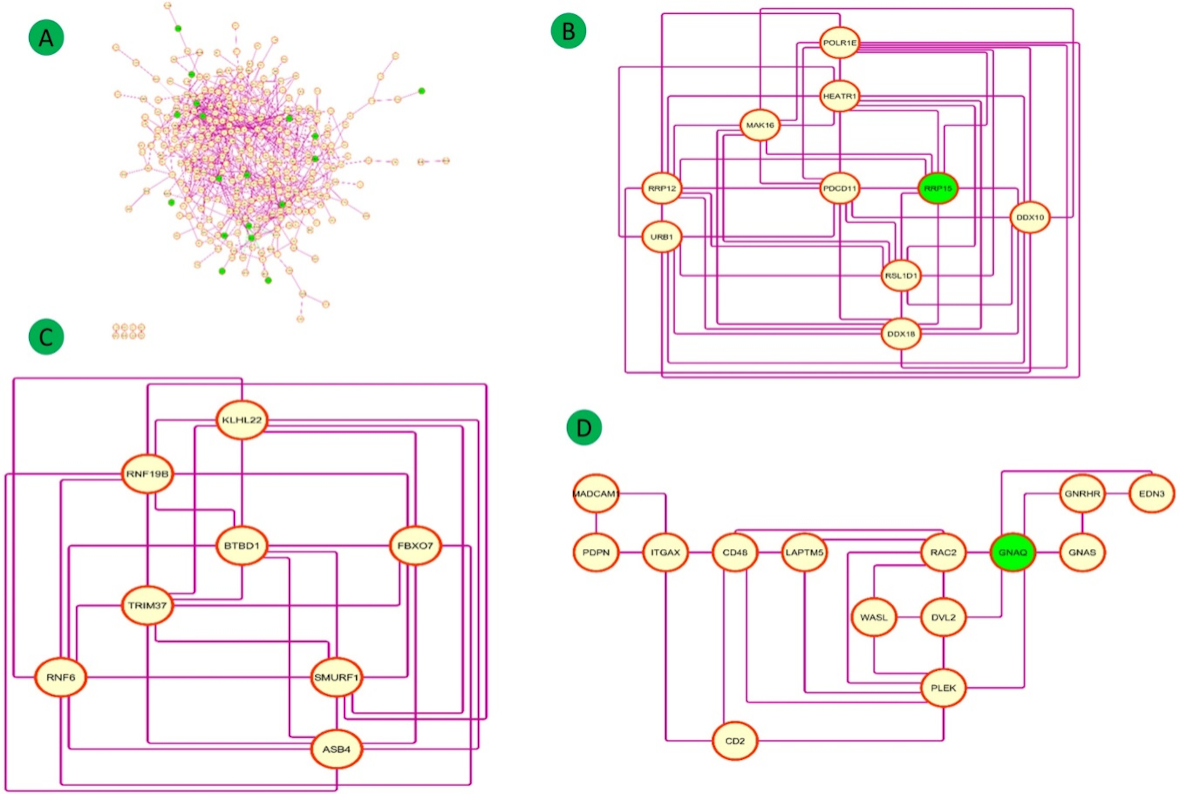
(A) Common and the top hub genes (green) in the protein-protein interaction network. (B-D) Clusters of the network.

**Figure 10:**
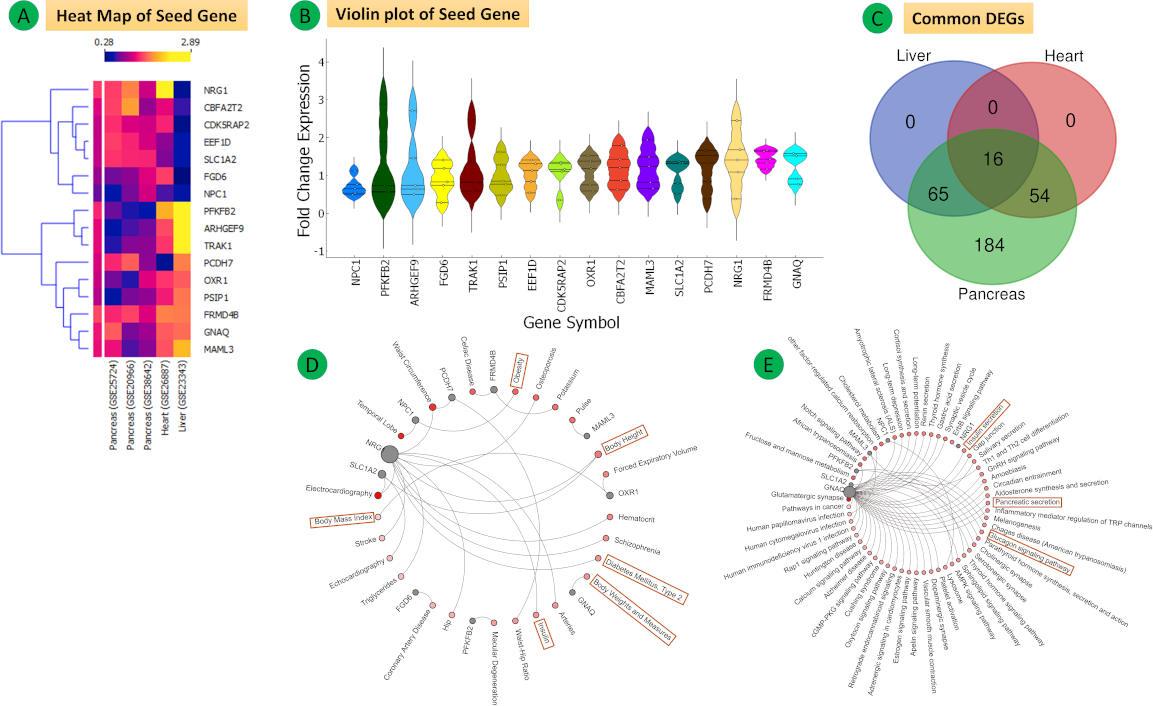
(A) Heat map of 16 common seed genes from the five data sets (pancreas, heart, and liver). The fold change expression levels are displayed in ascending order from blue to yellow. (B) Violin plot showing the entire fold change expression (FCE) distribution of all 16 common seed genes. (C) Venn diagram of common differentially expressed genes (DEGs). (D) Disease-gene interaction network. (E) Kyoto Encyclopedia of Genes and Genomes (KEGG) pathways.

**Figure 11:**
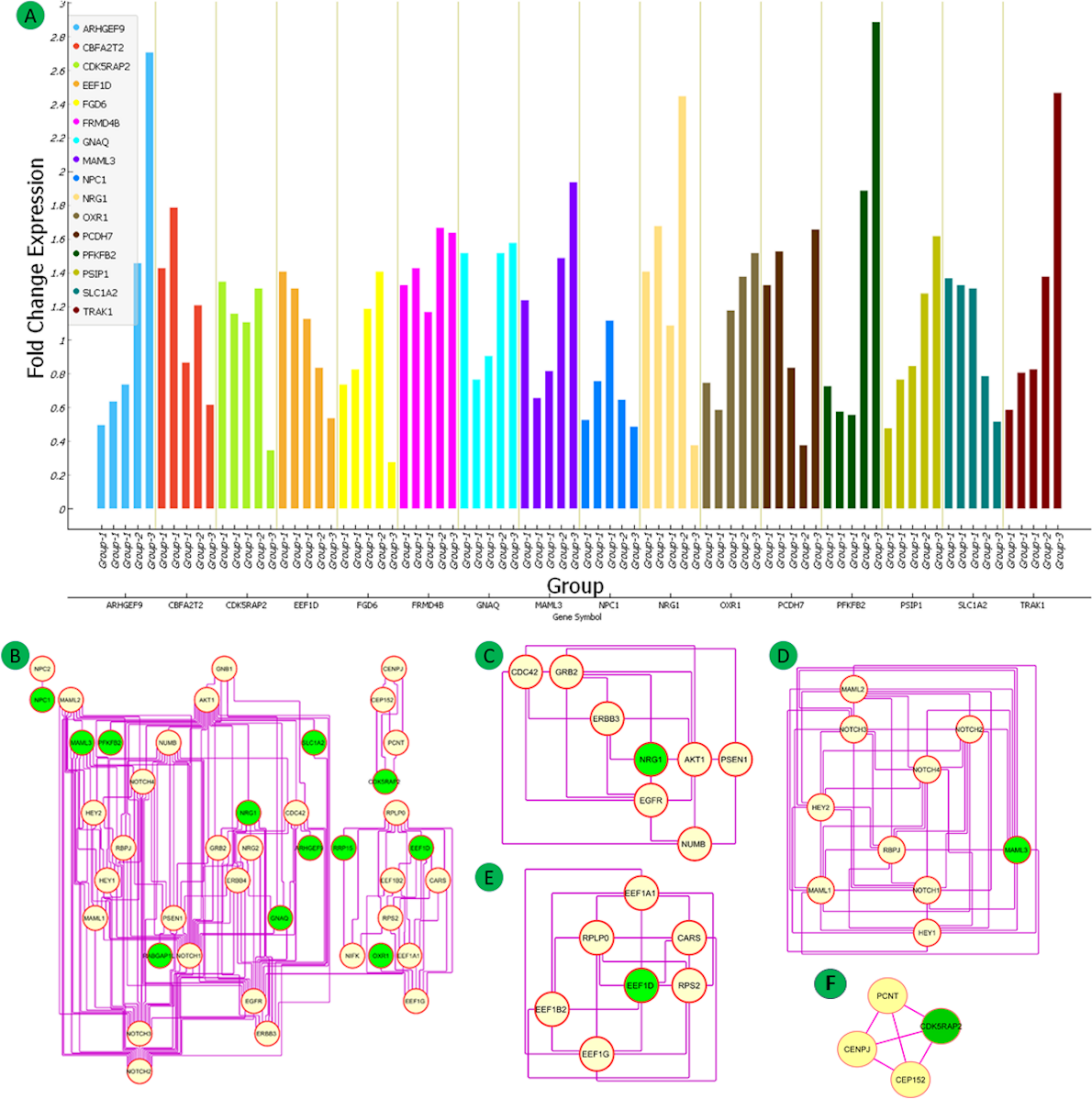
(A) Fold change expression levels of 16 common DEGs. (B) Top hub genes in the network (green) according to the criterion. (C, D, E, F) Clusters determined using MCODE.

**Figure 12:**
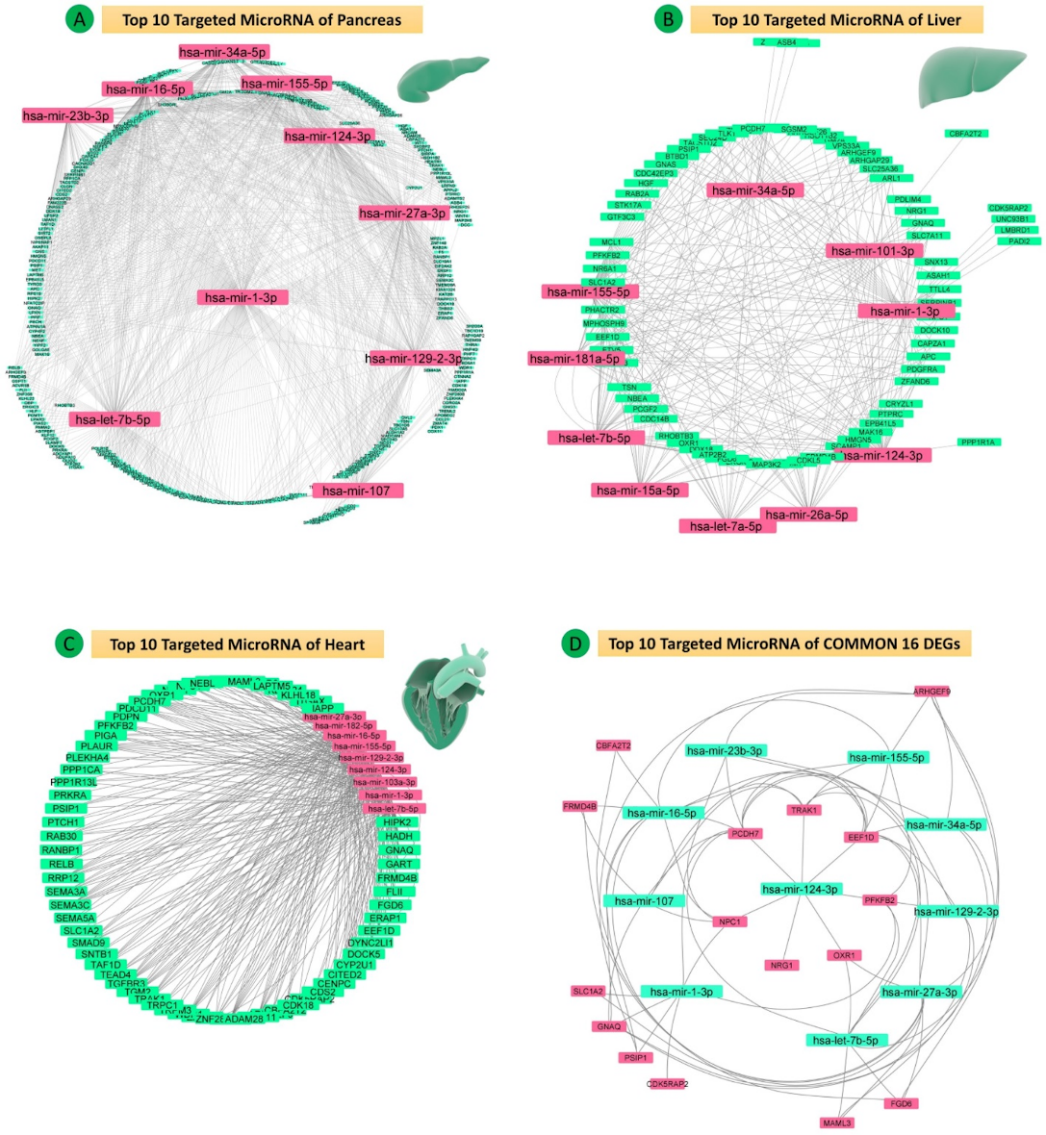
Protein-microRNAs interactions (top 10 ranked) for (A) pancreas data sets, (B) heart data set, (C) liver data set, and (D) 16 common differentially expressed genes (DEGs) of all five data sets.

**Figure 13:**
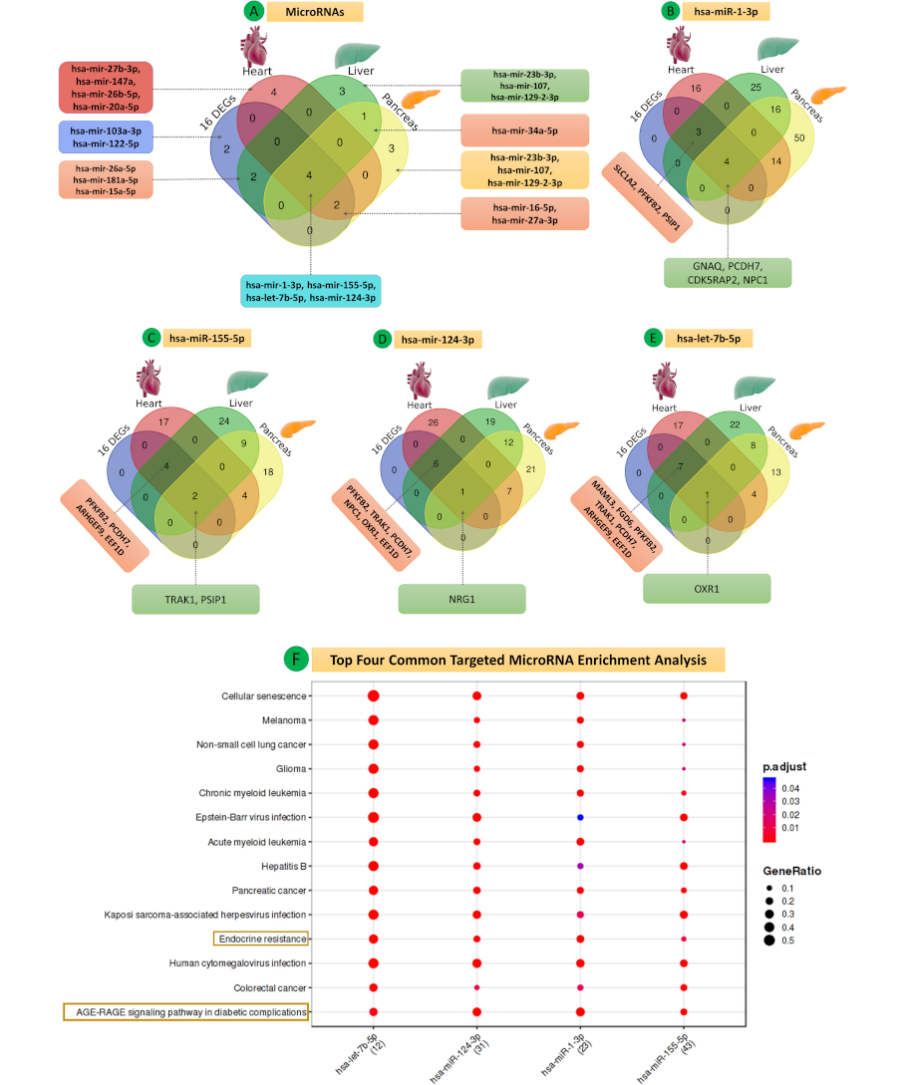
(A-E) Venn diagrams for (A) all microRNAs, (B) hsa-miR-1-3p, (C) hsa-miR-1-5p, (D) hsa-miR-155-5p, and (E) hsa-let-7b-5p with 16 common differentially expressed genes of the five data sets. (F) MicroRNA enrichment analysis of top four common targeted microRNAs.

**Figure 14:**
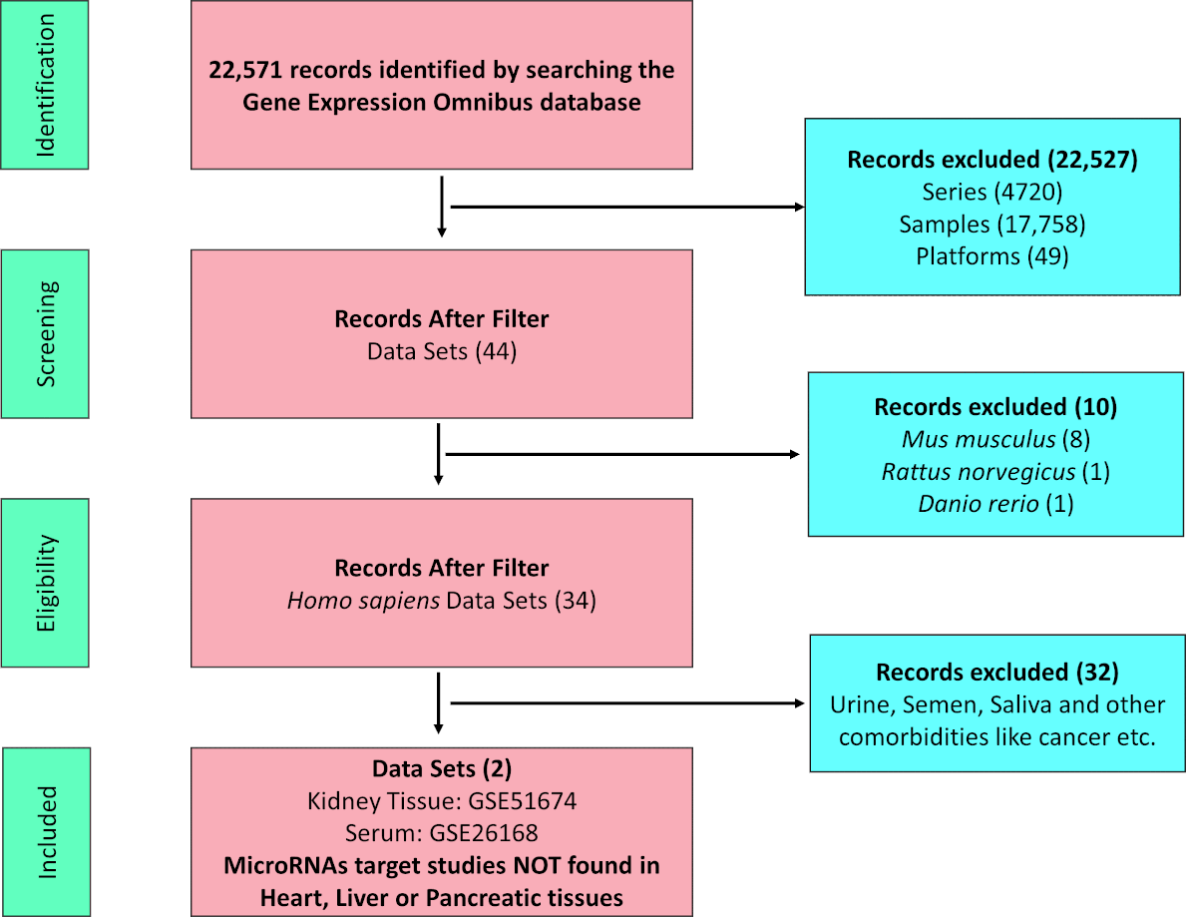
Flow diagram for the process of microRNA data collection with the number of data sets considered for inclusion.

The five mRNA expression profiles of the GSE38642, GSE25724, GSE20966, GSE26887, and GSE23343 data sets, including 125 samples of the pancreas, heart, and liver tissues of patients with T2DM and controls without diabetes, were included in this study. We extracted 2852, 8631, 5501, 4210, and 3754 DEGs, respectively. The following sections describe the analysis of the DEGs derived from the datasets, and shown in Figures 3-14. In the pancreas data sets (GSE38642, GSE25724, GSE20966), a total of 321 common mRNAs were identified, 69 of which were upregulated and 95 were downregulated (Figure 3A-H and Supplementary Tables S1-S3 in Multimedia Appendix 1). The quality control plots for the DEGs are shown in Figure S1 and Figure S2 of Multimedia Appendix 1.

These Group 1 (pancreas) DEGs were then overlapped with the heart expression profile data set GSE26887, revealing a total of 70 common differentially expressed mRNAs, 5 of which were downregulated and 5 were upregulated. A total of 28 mRNAs with regulation in the opposite direction were identified (Group 2) (Figure 5A-K, Tables S4-S7 in Multimedia Appendix 1). Further, the Group 1 DEGs were overlapped with the liver data set GSE23343, and a total of 82 common differentially expressed mRNAs were obtained, out of which 8 were upregulated, 1 was downregulated, and 27 were regulated in opposite directions (Figure 7A-I, Tables S8-S11 in Multimedia Appendix 1).

DEGs for all three groups were used to establish the PPI networks (Figure 6E, Figure 8E, Figure 9A).

### Functional Enrichment and KEGG Pathway Analysis

The enrichments for the three GO classes (BP, CC, and MF) of the 321 DEGs of Group 1 are shown in Figure 4B-D (also see Tables S12-S14 of Multimedia Appendix 1). KEGG pathway analysis showed that these genes were enriched in maturity-onset diabetes of the young, malaria, lysosome, insulin secretion, adrenergic signaling in cardiomyocytes, cell adhesion molecules, and T2DM pathways (Figure 4A, Table S15 of Multimedia Appendix 1).

The enrichments for the three GO classes of the 70 DEGs of Group 2 are shown in Figure 6B-D (also see Tables S16-S18 in Multimedia Appendix 1). The genes were mainly enriched in gap junction, melanoma, calcium signaling pathway, and GnRH signaling pathway (Figure 6A and Table S19 of Multimedia Appendix 1).

The enrichment terms for the three GO classes for the 82 DEGs in Group 3 are shown in Figure 8B-D (also see Tables S20-S22 in Multimedia Appendix 1). These genes were enriched in axon guidance (Figure 8A and Table S23 in Multimedia Appendix 1).

### PPI Network and Hub Gene Identification

#### Group 1

The 321 overlapping DEGs of the GSE38642, GSE25724, GSE20966 pancreas data sets were used to establish the PPI network, which constituted 321 nodes, 737 edges, and a PPI enrichment *P* value <.001 at medium confidence (0.4) (Figure 9A). The top three significant clusters within the PPI were selected.

Cluster 1 (MCODE Score=9.556, 10 nodes, 43 edges) included the genes *POLR1E, DDX10, URB1, HEATR1, DDX18, PDCD11, RSL1D1, RRP12, MAK16,* and *RRP15*, which are mainly associated with insulin pathway, transforming growth factor (TGF)-β receptor signaling, and the mammalian target of rapamycin (mTOR) signaling pathway (Figure 9B).

Cluster 2 (MCODE score=8.000, 8 nodes, 28 edges) included the genes *TRIM37, BTBD1, RNF19B, ASB4, KLHL22, SMURF1, FBXO7,* and *RNF6*, which are mainly associated with insulin pathway, insulin-like growth factor 1 (IGF1) pathway, class I phosphatidylinositol-3-kinase (PI3K) signaling events mediated by protein kinase B (AKT), TGF-β receptor signaling, mTOR signaling pathway, platelet-derived growth factor receptor-beta signaling pathway, and epidermal growth factor (EGF) receptor (ERBB1) signaling pathway (Figure 9C).

Cluster 3 (MCODE score=4.000, 14 nodes, 26 edges) included the genes *CD2, CD48, EDN3, GNAS, ITGAX, PDPN, GNRHR, RAC2, MADCAM1, WASL, GNAQ, PLEK, LAPTM5*, and *DVL2*, which are associated with platelet activation, signaling, and aggregation; hemostasis, cell surface interactions at the vascular wall; integrin family cell surface; and IGF1 pathway (Figure 9D).

#### Group 2

The 70 overlapping DEGs of GSE26887 and coexpressed genes with Group 1 (321 genes) were used to establish the PPI network composed of 70 nodes, 32 edges, and a PPI enrichment *P* value of .05 at medium confidence (0.4). The top two significant clusters within the PPI were selected using the MCODE plugin of Cytoscape software (Figure 6E).

Cluster 1 (MCODE score=3.333, 4 nodes, 5 edges) included the genes *RRP12, PDCD11, RRP15*, and MRPL3 (Figure 6F). Cluster 2 (MCODE score=3.000, 3 nodes, 3 edges) included the genes *PLEK, GNAQ*, and *IQSEC1*, which are mainly associated with platelet activation, signaling, and aggregation; hemostasis; class I PI3K signaling events mediated by AKT; insulin pathway; mTOR signaling pathway; IGF1 pathway; and EGF receptor (ERBB1) signaling pathway (Figure 6G).

#### Group 3

The 82 overlapping DEGs of GSE23343 and the coexpressed genes of Group 1 (321 DEGs) were used to establish the PPI network composed of 82 nodes, 56 edges, and a PPI enrichment *P* value of .02 at medium confidence (0.4). The top two significant clusters are shown in Figure 8E.

Cluster 1 (MCODE score=03, 3 nodes, 3 edges) included the genes *BTBD1, ASB4*, and *KLHL22*, which were mainly associated with PI3K/AKT signaling in cancer (Figure 8F).

Cluster 2 (MCODE score=03, 3 nodes, 3 edges) included the genes *DDX18, MAK16*, and *RRP15*, which were mainly associated with insulin pathway, mTOR signaling pathway, IGF1 pathway, and EGF receptor (ERBB1) signaling pathway (Figure 8G).

### Common Genes Among All Groups

A total of 16 overlapping DEGs were identified in all three groups. The hub genes of all data sets were *ARHGEF9, CBFA2T2, CDK5RAP2, EEF1D, FGD6, FRMD4B, GNAQ, MAML3, NPC1, NRG1, OXR1, PCDH7, PFKFB2, PSIP1, SLC1A2*, and *TRAK1* (Table S24 in Multimedia Appendix 1). All 16 hub genes belonging to the five data sets were analyzed with the help of an expression heat map, violin plot, and Venn diagram, and their fold change expression levels were compared by bar plots and analyzed by the disease-gene interaction network and KEGG pathway (Figure 10A-E and Figure 10F; Table S25 in Multimedia Appendix 1).

The PPI network of the 16 hub genes and their related genes was established by protein STRING analysis. We selected 4 clusters from the PPI network using MCODE (Figure 11B). Cluster 1 (MCODE score=10, 10 nodes, 45 edges) included the genes *MAML1, HEY2, NOTCH3, MAML3, NOTCH2, MAML2, NOTCH1, HEY1, RBPJ,* and *NOTCH4*. The analysis also showed that cluster 1 contains *MAML3* as a seed gene (Figure 11D). Cluster 2 (MCODE score=6.667, 7 nodes, 20 edges) included the genes *EEF1A1, EEF1B2, EEF1G, RPLP0, RPS2, CARS,* and *EEF1D*, with *EEF1D* as a seed gene (Figure 11E). Cluster 3 (MCODE score=5.714, 8 nodes, 20 edges) included the genes *AKT1, NUMB, EGFR, ERBB3, GRB2, CDC42, PSEN1*, and *NRG1*, with *NRG1* as a seed gene (Figure 11C). Cluster 4 (MCODE score=4, 4 nodes, 6 edges) included the genes *CDK5RAP2, CEP152, CENPJ*, and *PCNT*, with *CDK5RAP2* as the seed gene (Figure 11F).

### Integrative Gene Expression and Meta-analysis

The number of genes with an adjusted *P* value <.05 for each data set revealed 4, 0, 3533, 171, and 1 significant genes from the meta-analysis, including *ARHGEF9, SAMSN1, SLC1A2, RABGAP1L, OXR1, GNAQ, CBFA2T2*, and *RRP15*. The 16 hub genes obtained from the gene expression meta-analysis are shown in Table S26 of Multimedia Appendix 1.

### MicroRNA and Hub Gene Network

To investigate the regulatory relationship of the identified hub genes, their targeting miRNAs, and coexpressed network, the top 10 ranked DEG-targeting miRNAs were selected based on degree and betweenness values. The top 10 targeting miRNAs for the three groups were hsa-let-7b-5p, hsa-mir-107, hsa-mir-124-3p, hsa-mir-129-2-3p, hsa-mir-1-3p, hsa-mir-155-5p, hsa-mir-16-5p, hsa-mir-23b-3p, hsa-mir-27a-3p, and hsa-mir-34a-5p in Group 1 (pancreas); hsa-mir-16-5p, hsa-mir-124-3p, hsa-mir-1-3p, hsa-mir-27a-3p, hsa-let-7b-5p, hsa-mir-155-5p, hsa-mir-20a-5p, hsa-mir-26b-5p, hsa-mir-27b-3p, and hsa-mir-147a in Group 2 (heart); and hsa-mir-1-3p, hsa-mir-155-5p, hsa-mir-124-3p, hsa-let-7b-5p, hsa-mir-34a-5p, hsa-mir-101-3p, hsa-mir-15a-5p, hsa-mir-26a-5p, hsa-mir-181a-5p, and hsa-let-7a-5p in Group 3 (liver). The common hub genes were targeted by hsa-mir-16-5p, hsa-mir-27a-3p, hsa-let-7a-5p, hsa-let-7b-5p, hsa-mir-101-3p, hsa-mir-1-3p, hsa-mir-124-3p, hsa-mir-103a-3p, hsa-mir-122-5p, and hsa-mir-155-5p.

Four common miRNAs (hsa-let-7b-5p, hsa-mir-155-5p, hsa-mir-124-3p, hsa-mir-1-3p) were found in all three groups, targeting the 16 hub DEGs. The miRNAs and PPI networks representing multiple targeted nodes (DEGs) of particular miRNAs for all groups are shown in Figure 12A-D.

The common DEGs found in all three groups are targeted by hsa-miR-1-3p (*GNAQ, PCDH7, CDK5RAP2, NPC1*), hsa-let-7b-5p (*OXR1*), hsa-mir-155-5p (*TRAK1, PSIP1*), and hsa-mir-124-3p (*NRG1*). The common targeting important miRNAs (hsa-let-7b-5p, hsa-mir-155-5p, hsa-mir-124-3p, hsa-mir-1-3p) were mainly involved in the advanced glycation end products (AGE)-receptor for advanced glycation end products (RAGE) signaling pathway in diabetic complication and endocrine resistance (Figure 13A-F, Tables S27-S40 in Multimedia Appendix 1).

### Target MiRNA Validation from Available Data Sets

To validate our miRNA prediction, we searched the database again and performed a thorough review of available miRNA data sets for T2DM. Our search yielded two miRNA data sets from renal tissue (GSE51674) and serum (GSE26168) samples. The flow diagram for the miRNA data set search is shown in Figure 14. However, we were not able to find any miRNA data set pertaining to the heart, pancreas, or liver tissue. Interestingly, on analysis of the data sets obtained from the renal tissue and serum, we observed a significant alteration for our predicted miRNAs in the renal tissue, which was conspicuously absent in the serum (Table 2). We assessed the expression of our predicted miRNAs in the renal tissue and serum by comparing the adjusted *P* values for both sample types. This analysis revealed that although the expression of miRNAs was significantly altered in renal tissues from patients with T2DM, the same was not observed in serum when compared with healthy controls. Our analysis highlights a paradoxical difference in the alteration of miRNAs in tissue and serum in T2DM.

**Table 2:** Validation of the fold change in expression levels of common microRNAs in the GSE51674 (kidney) and GSE26168 (serum) data sets.

MicroRNA		Adjusted *P* value	*P* value	*t*	B	FC^a^	logFC
**GSE51674 (kidney)**							
	hsa-miR-124*^b^	<.001	<.001	–4.83	–0.85	0.50	–1.00
	hsa-miR-1	<.001	<.001	6.06	0.92	19.42	4.28
	hsa-miR-155	<.001	<.001	20.96	12.12	74.56	6.22
	hsa-let-7b	<.001	<.001	4.60	–1.20	2.55	1.35
**GSE26168 (serum)**							
	hsa-miR-124*	.46	.23	1.23	–6.22	1.01	0.02
	hsa-miR-1	.86	.82	0.23	–6.95	1.00	0.00
	hsa-miR-155	.46	.08	1.86	–5.33	1.04	0.05
	hsa-let-7b	.46	.16	1.45	–5.94	237.61	7.89

^a^FC: fold change.

^b^*indicates the star strand for miR-124.

### Functional Enrichment of MiRNAs

The functional enrichment and pathway analysis by MIENTURNET revealed the top significant pathways for hsa-let-7b-5p, hsa-miR-124-3p, hsa-miR-1-3p, and hsa-miR-155-5p, including the PI3K-AKT signaling pathway (hsa-let-7b-5p, hsa-miR-124-3p, hsa-miR-1-3p, hsa-miR-155-5p), endocrine resistance (hsa-let-7b-5p, hsa-miR-124-3p, hsa-miR-1-3p, hsa-miR-155-5p), AGE-RAGE signaling pathway in diabetic complications (hsa-let-7b-5p, hsa-miR-124-3p, hsa-miR-1-3p, hsa-miR-155-5p), lipid and atherosclerosis (hsa-let-7b-5p, hsa-miR-1-3p, hsa-miR-155-5p), insulin signaling pathway (hsa-let-7b-5p, hsa-miR-124-3p, hsa-miR-1-3p), mitogen-activated protein kinase (MAPK) signaling pathway (hsa-let-7b-5p, hsa-miR-124-3p, hsa-miR-155-5p), fluid sheer stress and atherosclerosis (hsa-miR-124-3p, hsa-miR-1-3p), adipocytokine signaling pathway (hsa-miR-124-3p), diabetic cardiomyopathy (hsa-miR-124-3p, hsa-miR-1-3p), insulin resistance (hsa-miR-124-3p), carbohydrate digestion and absorption (hsa-miR-124-3p), regulation of lipolysis in adipocytes (hsa-miR-124-3p), glucagon signaling pathway (hsa-miR-124-3p), and TGF-β signaling pathway (hsa-miR-155-5p) (see Figure 13 and Table S41 of Multimedia Appendix 1).

## Discussion

### Principal Findings

Diabetes develops because of dysregulated β-cell and adipose-tissue responses to chronic fuel excess, which result in so-called nutrient spillover, insulin resistance, and metabolic stress. The latter causes multiple organ damage. However, insulin resistance, while forcing β-cells to work harder, may also have an important defensive role against nutrient-related toxic effects in tissues such as the heart [32]. The liver, which primarily regulates glucose homeostasis in the body, has a strong association with diabetes. Liver disease in diabetes can further be classified into liver disease related to diabetes, hepatogenous diabetes, and liver disease occurring coincidentally with diabetes mellitus [33]. Recently, knowledge on the pathogenesis and management of diabetes mellitus has been expanding; however, the disease is far from being effectively managed in a large proportion of patients. In silico analysis of disease pathways and exploration of various disease-related genes and their regulatory molecules have revealed unforeseen vistas. In this study, we analyzed tissue-specific microarray gene expression data sets from publicly available repositories employing a network-based bioinformatics pipeline. We identified DEGs common to different tissues of patients with T2DM and constructed diseasome networks to provide insights into the interactions of the genes. These DEGs enabled the identification of associated dysregulated molecular pathways in tissues and related GO terms. A large number of pathways and GO categories were reduced by manual curation after filtering using a *P* value threshold of .05.

Our analysis supports that diabetes is a multifactorial disease caused by multiple complex systems, with an abundant crossover between signaling pathways. For each data set included in the study, comprehensive analysis focusing on biological function and interaction of T2DM-related genes provided valuable information to understand the pathogenic effect of DEGs in various organs, including the heart, liver, and pancreas, of patients with diabetes. In this study, five mRNA expression profile data sets (GSE38642, GSE25724, GSE20966, GSE26887, and GSE23343), including 125 samples of the pancreas, heart, and liver tissues of patients with T2DM and controls without diabetes, were analyzed. A total of 16 seed genes were obtained after the final analysis. Some of these genes have been reported to play significant roles in T2DM and its related comorbidities. In a similar study that included DEG screening from a genome-wide association study (GWAS) catalog, Gupta and Vadde [34] identified four hub gene candidates, related signaling pathways, target miRNAs, and transcription factors. However, their selection criteria of the data sets chosen for analysis were different than those adopted in this study, which possibly accounts for the difference in results.

Neuregulin 1 (NRG1) and ERBB receptors are involved in glucose homeostasis. NRG1-ERBB pathway activation affects glucose metabolism in the liver. Mice with chronic NRG1 treatment showed increased p38 phosphorylation in the liver and improved glucose tolerance [35]. Myocardial NRG1/ERBB is altered during postmyocardial infarction heart failure associated with diabetes. NRG1 can improve the antioxidative function of the mitochondria, and thereby increase the proliferation and decrease the apoptosis of cardiomyocytes via ERBB/AKT signaling. This can explain the upregulated expression of *NRG1* found in the cardiac tissue of patients with T2DM in our study. Moreover, the dysregulated insulin signaling pathway modifies titin-based cardiomyocyte tension, modulates diastolic function, impairs cyclic guanosine monophosphate (cGMP)–cGMP-dependent protein kinase signaling, and elevates protein kinase C-α activity, thereby causing titin-based cardiomyocyte stiffening in diabetic hearts. Chronic NRG1 application has shown promising results in the modulation of titin properties in T2DM-associated heart failure with a preserved ejection fraction [36]. Further, there are reports showing that hyperglycemia impairs NRG1/ERBB2 signaling by disrupting the balance between NRG1 isoforms, decreasing the expression of erbin, and correspondingly activating the MAPK pathway, ultimately aiding in the development of diabetic peripheral neuropathy [37]. Again, the downregulation of *NRG1* expression in the liver found in this study points toward dysregulated glucose homeostasis.

*PFKFB2* encodes 6-phosphofructo-2-kinase/fructose 2,6-bisphosphatase (PFK2/FBPase-2) isoform 2, a bifunctional enzyme involved in the synthesis and degradation of fructose 2,6-bisphosphate. Enhanced hepatic glycolysis in mice achieved by overexpressing PFK2/FBPase-2 in the liver resulted in reduced body weight and visceral fat content. PFK2/FBPase-2 is also a binding partner for glucokinase, which plays a pivotal role in the rate-limiting step of glucose-stimulated insulin secretion in pancreatic β-cells, and regulates obesity, insulin secretory dysfunction, and T2DM [38,39]. The loss of PFK2 content as a result of reduced insulin signaling impairs its regulatory function of glycolysis and elevates the levels of early glycolytic intermediates. Although this may be beneficial in the fasting state to conserve systemic glucose, it represents a pathological impairment in diabetes mellitus [40]. Interestingly, *PFKFB2*, among a few other genes, showed opposing expression changes in the pancreas (downregulation) and heart (upregulation). This is likely due to the impaired insulin secretion pathway in pancreatic β-cells, in which PFKFB2 plays an important role [39]. Moreover, PFKFB2 is known to alleviate myocardial injury; hence, the increased expression level in the heart is possibly a protective mechanism [41].

CDK5 regulatory subunit associated protein (*CDK5RAP*) 1, 2, and 3 were all found to be differentially upregulated in four data sets, except GSE23343 in which these genes were downregulated. These genes have been associated with neuronal development and spindle checkpoint function [42]. FRMD4B plays a vital role in cardiac activity regulation. However, the effect varies in different populations due to polymorphisms. FRMD4B has shown to be associated with ischemic heart failure in a European population but not in other populations [43]. The G-protein Gq, encoded by *GNAQ*, is a crucial key regulator of the insulin secretion pathway that is involved in glucose metabolism, and a functional *GNAQ* promoter haplotype was associated with altered Gq expression and with insulin resistance and obesity in women with polycystic ovary syndrome [44]. The Niemann-Pick type C1 (NPC1) protein regulates the transport of cholesterol and fatty acids from late endosomes/lysosomes and has a central role in maintaining lipid homeostasis. In humans, GWAS and post-GWAS highlighted the implication of common variants in *NPC1* in adult-onset obesity, body fat mass, and T2DM. Heterozygous human carriers of rare loss-of-function coding variants in *NPC1* display an increased risk of morbid adult obesity [45]. Another significant DEG pair was orexin A and B, which regulate a variety of physiological functions. The biological effects of these neuropeptides occur through OXR1, a G-protein coupled receptor. There is growing evidence that orexins regulate body weight, glucose homeostasis, and insulin sensitivity, and promote energy expenditure, thus protecting against obesity by interacting with brown adipocytes. Further, orexins control brown and white adipocytes as well as pancreatic α- and β-cell functions [46,47]. Single-cell RNA sequencing from samples of patients with gestational diabetes mellitus revealed *SLC1A2* as a novel marker for syncytiotrophoblasts [48]. Such cell-type-specific marker genes in particular disease states can open new avenues of tissue-targeted therapeutic intervention. Among the other DEGs, *EEF1D* regulates lipid synthesis via the PI3K/AKT, PPAR, and AMPK pathways [49]. CBFA2T2 is a key regulator of adipogenic differentiation through CEBPA [50]. Further, these seed genes were analyzed as possible miRNA targets in silico, which revealed the top 10 miRNAs for each of the pancreas, liver, and heart tissues, as well as for the 16 seed genes. The role of miRNAs in the regulation of the underlying pathogenic mechanisms of diabetes and diabetic complications is well established [7,51] Several of the target miRNAs for the seed genes have already been explored in T2DM, and our in silico analysis further confirms their candidature as potential biomarkers as well as therapeutic targets. In fact, miR-124-3p was interconnected to 7 of the 16 seed genes. Pan et al [52] studied mouse primary hepatocytes and observed that regulation of miR-124-3p plays an important role in turning the hepatocytes into insulin-producing cells. A recent analysis of weighted genes in diabetic retinopathy concluded miR-124-3p to be a pivotal regulatory molecule in the underlying pathogenesis [6]. Furthermore, in isolated myocardial cells, *NRG1* expression was observed to be downregulated while miR-124-3p expression was upregulated in ischemia/reperfusion injury [53], which also supports our finding of this miRNA-mRNA target interaction. The miRNA hsa-miR-124-3p affects the immune status of patients with T2DM through its interaction with the obesity-related immune cytokines [54].

Three other miRNAs, namely miR-155-5p, miR-1-3p, and let-7b-5p, were also commonly identified in all three groups. Likewise, the role of miR-155-5p in diabetes has been widely studied, especially as a marker in diabetic kidney disease (DKD) [55-57]. The expression of miR-155-5p is positively associated with urinary microalbumin and has good diagnostic and prognostic value in patients with DKD [56]. Further, dihydromyricetin attenuates renal interstitial fibrosis by regulating PTEN signaling, a critical element in the pathogenesis of DKD, through miR-155-5p [58,59]. Recently, Zhou and colleagues [60] showed that metformin can relieve inflammation and fibrosis in patients with DKD by acting through an inflammation axis involving miR-155-5p. Some recent studies have also shown that miR-155-5p interferes with immune dysregulation in COVID-19 patients with diabetes or other comorbidities [61,62]. Further, all four miRNAs were found to be involved in regulating the endocrine resistance and AGE-RAGE pathways, which is in line with recent findings [63].

The differing trend in miRNA expression observed in our comparison of miRNA data sets from serum and renal tissue in T2DM highlights the necessity to further explore the tissue-specific alterations in T2DM to better comprehend its role in various tissues.

### Limitations

The main limitation of this study is that it was based on an in silico analysis; therefore, further validation of the identified novel hub genes and miRNAs is still required based on laboratory experiments with human T2DM samples. The data sets were compiled using different arrays on the Affymetrix platform, and the patient populations belong to multiple ethnic groups, which may account for some of the variability in the results. Furthermore, the predicted miRNAs in this study could not be validated within the same tissue data sets. However, the functional enrichment for the miRNAs highlighted some significant pathways related to T2DM, its complications, and its pathogenic mechanisms.

### Conclusion

The aim of this study was to identify the tissue-specific differential expression of genes, especially pertaining to the heart, liver, and pancreas, in T2DM. From Group 1 (pancreas: 374 DEGs), Group 2 (heart: 86 DEGs), and Group 3 (liver: 97 DEGs), we identified a total of 16 common DEGS (*ARHGEF9, CBFA2T2, CDK5RAP2, EEF1D, FGD6, FRMD4B, GNAQ, MAML3, NPC1, NRG1, OXR1, PCDH7, PFKFB2, PSIP1, SLC1A2*, and *TRAK1*) in the selected data sets. Further, we identified the top four common miRNAs (hsa-let-7b-5p, hsa-miR-124-3p, hsa-miR-1-3p, has-miR-155-5p) targeting the 16 common DEGs. Although we were not able to find any miRNA data set pertaining to the heart, pancreas, or liver tissue, we observed significant alterations of our predicted miRNAs in renal tissue. Interestingly, this significant alteration was conspicuously absent in the serum. The miRNAs identified in this study are involved in regulating various pathways, including the PI3K-AKT signaling pathway, endocrine resistance, and the AGE-RAGE signaling pathway. Moreover, the differing trend in miRNA expression observed in our comparison of miRNA data sets from the serum and renal tissue in T2DM highlights the necessity to further explore the tissue-specific alteration in T2DM to better comprehend its role in various tissues.

## Appendix

Multimedia Appendix 1 Supplementary data: Figures S1-S2, Tables S1-S41.

## Abbreviations

AGE: advanced glycation end products
AKT: protein kinase B
BP: biological process
CC: cellular component
CDK5RAP: CDK5 regulatory subunit associated protein
cGMP: cyclic guanosine monophosphate
DAVID: Database for Annotation, Visualization and Integrated Discovery
DEG: differentially expressed gene
DKD: diabetic kidney disease
EGF: epidermal growth factor
ERBB1: epidermal growth factor receptor
GEO: Gene Expression Omnibus
GO: Gene Ontology
GWAS: genome-wide association study
IGF1: insulin-like growth factor 1
KEGG: Kyoto Encyclopedia of Genes and Genomes
limma: linear models for microarray data
MAPK: mitogen-activated protein kinase
MCODE: Molecular Complex Detection
MF: molecular function
MIENTURNET: MicroRNA Enrichment Turned Network
miRNA: microRNA
mTOR: mammalian target of rapamycin
NPC1: Niemann-Pick type C1
NRG1: neuregulin 1
PFK2: 6-phosphofructo-2-kinase/fructose 2,6-bisphosphatase isoform 2
PI3K: phosphoinositide 3-kinase
PPI: protein-protein interaction
RAGE: receptor of advanced glycation end products
STRING: Search Tool for the Retrieval of Interacting Genes/Proteins
T2DM: type 2 diabetes mellitus
TGF: transforming growth factor
