# Supplementary material for: Novel Molecular Networks and Regulatory MicroRNAs in Type 2 Diabetes Mellitus: Multiomics Integration and Interactomics Study
Source: JMIR Bioinform Biotechnol. 2022 Feb 23;3(1):e32437. doi: 10.2196/32437 (PMC11135235; doi:10.2196/32437)
Supplement: Multimedia Appendix 1 [file bioinform_v3i1e32437_app1.pdf]

Multimedia Appendix 1:

Novel Molecular Networks and Regulatory MicroRNAs in Type 2 Diabetes Mellitus: Multiomics Integration and Interactomics Study

Manoj Khokhar,[1] Dipayan Roy,[1] Sojit Tomo,[1] Ashita Gadwal,[1] Praveen Sharma[1], Purvi Purohit,[1]*

[1]Department of Biochemistry, All India Institute of Medical Sciences (AIIMS), Jodhpur, Rajasthan, India

Running head: Interactomics in Type 2 Diabetes

***Corresponding author**

Dr Purvi Purohit

Additional Professor

Department of Biochemistry

AIIMS, Jodhpur, Rajasthan- 342005

Email: dr.purvipurohit@gmail.com

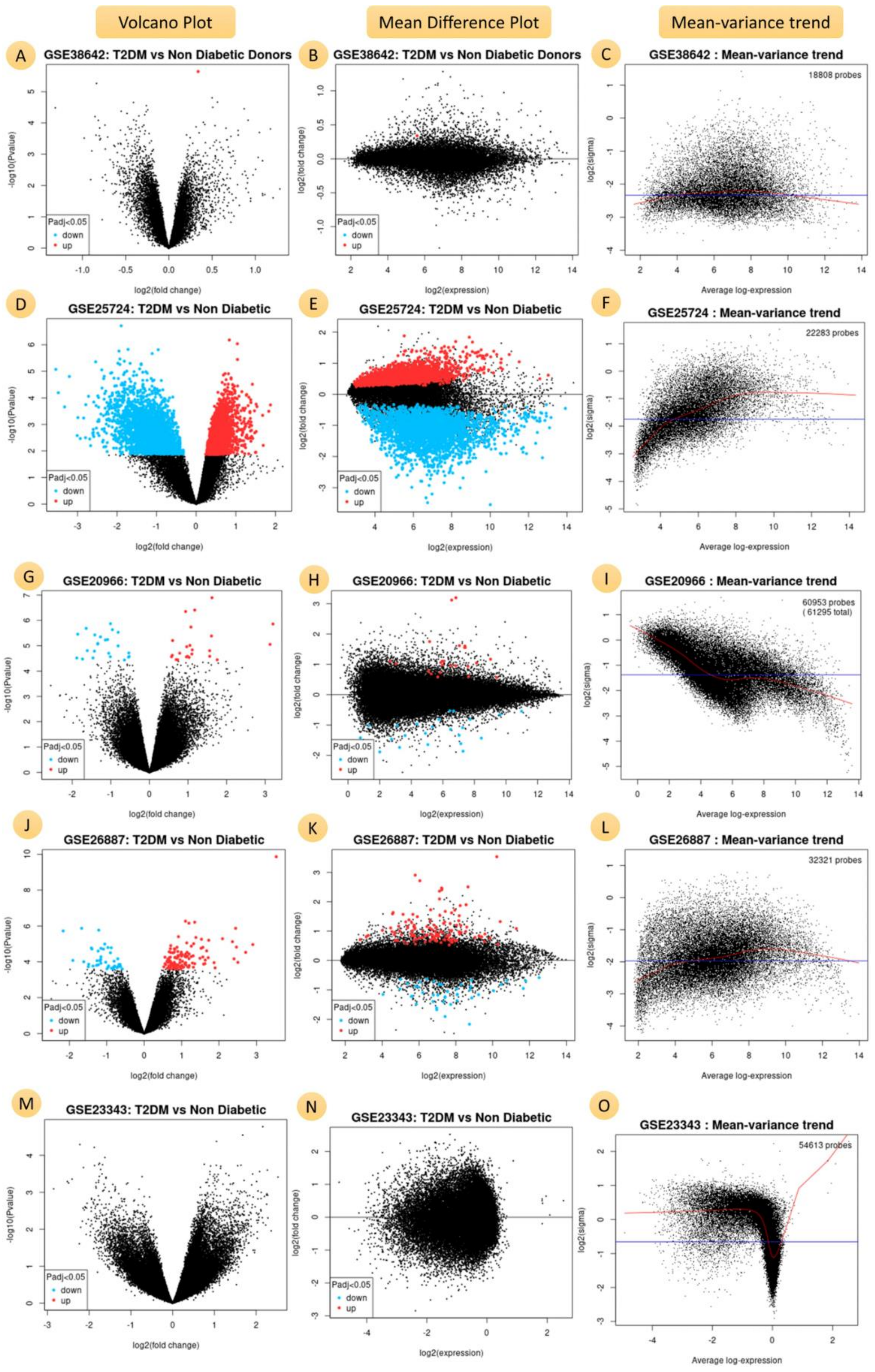

Figure S1: Volcano plot, Mean Difference plot, Mean Variance Trend show the DEGs in all five GSE38642, GSE25724, GSE20966, GSE26887, and GSE23343.

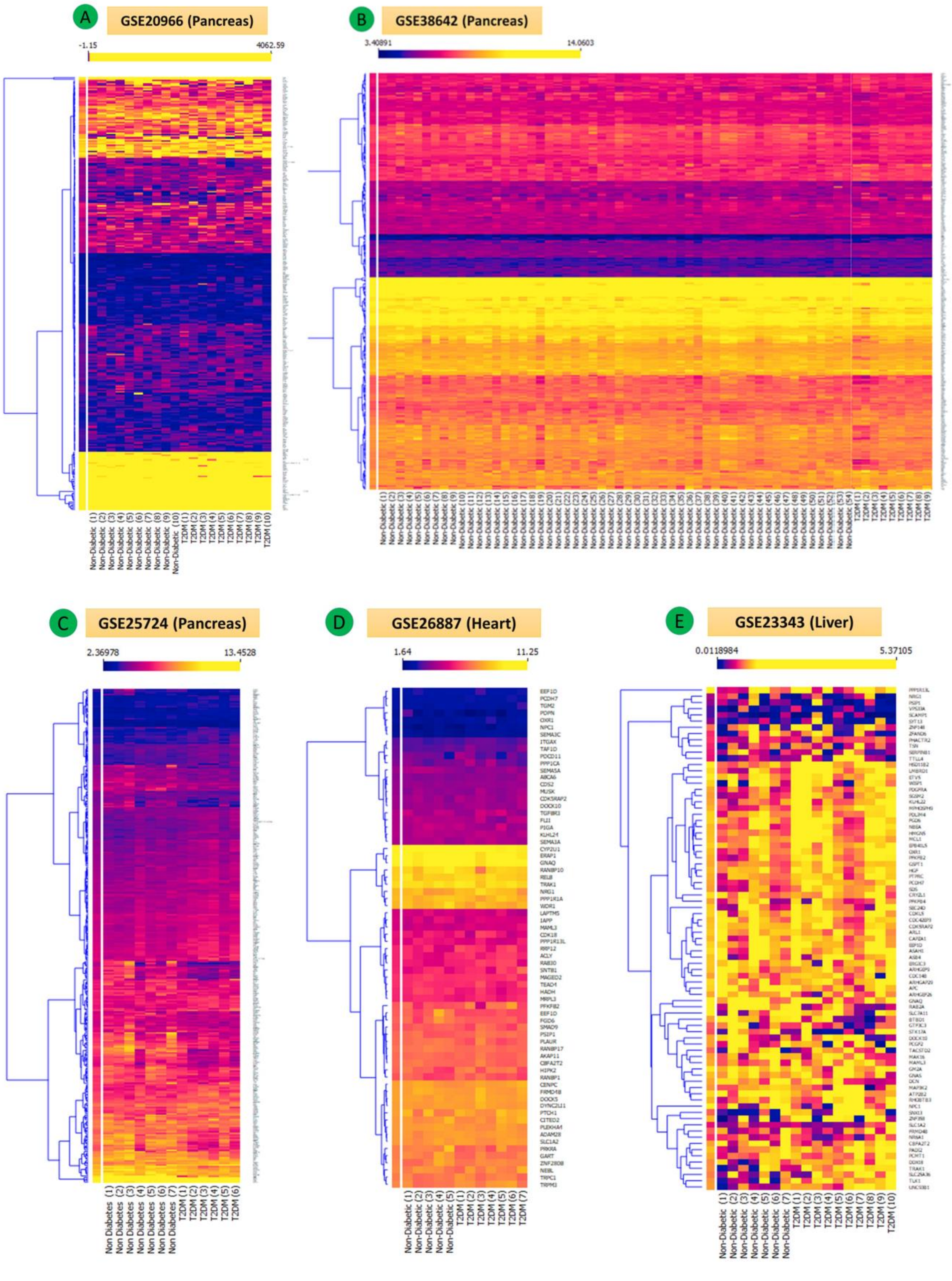

Figure S2. Heatmap of the Common Gene expression of individual data sets for all three groups, (A). GSE20966 (Pancreas) for 10 Samples of Nondiabetic and 10 Samples of T2DM samples of data sets; (B). GSE38642 (Pancreas) for 54 Samples of Nondiabetic and 9 Samples of T2DM samples; (C). GSE25724 (Pancreas) for 07 Samples of Nondiabetic and 06 Samples of T2DM samples; (D). GSE26887 (Heart) for 05 Samples of Nondiabetic and 07 Samples of Diabetic data sets; (E). GSE23343 (Liver) for 07 Samples of Nondiabetic and 10 Samples of Diabetic data sets.

Table S1. Complete Common differentially expressed genes of diabetic pancreatic tissue. (*Group: 1*)

	Pancreas GSE25724		Pancreas GSE20966		Pancreas GSE38642	
Gene Symbol	FCE	p-Value	FCE	p-Value	FCE	p-Value
ABAT	0.28	0.00	0.78	0.03	0.78	0.03
ABCA6	1.31	0.01	1.4	0.04	1.15	0.01
ABCC4	0.63	0.00	0.68	0.03	1.19	0.02
ABCC8	0.36	0.01	0.77	0.00	0.54	0.00
ABHD10	0.57	0.00	0.8	0.02	0.74	0.00
ACLY	0.5	0.00	0.55	0.03	0.83	0.04
ACVR1B	1.54	0.01	0.79	0.01	0.86	0.00
ADAM28	1.42	0.00	1.82	0.03	1.51	0.00
ADAMTS2	1.59	0.00	0.66	0.01	0.78	0.00
ADCYAP1	0.27	0.01	0.66	0.03	0.72	0.03
ADD2	1.46	0.01	2.75	0.00	1.13	0.04
AGTPBP1	0.49	0.01	0.79	0.05	0.83	0.00
AKAP11	0.22	0.00	0.7	0.01	0.86	0.04
ALDH1A2	1.51	0.00	1.66	0.06	1.28	0.02
ALS2CL	1.82	0.00	0.81	0.03	1.16	0.05
APC	0.36	0.00	0.71	0.02	0.82	0.01
APOBEC2	0.63	0.02	2	0.00	0.78	0.03
APPL2	0.73	0.01	0.74	0.04	0.89	0.01
ARG2	0.2	0.00	0.47	0.02	0.57	0.00
ARHGAP26	1.73	0.01	0.56	0.00	1.18	0.04
ARHGAP29	0.4	0.02	2.29	0.01	1.17	0.03
ARHGEF26	1.27	0.02	0.52	0.05	0.84	0.00
ARHGEF9	0.5	0.02	0.64	0.00	0.74	0.01
ARL1	0.38	0.00	0.8	0.01	0.88	0.01
ARL4D	1.24	0.05	1.43	0.06	0.82	0.03
ASAH1	0.49	0.01	1.18	0.03	0.9	0.04
ASB4	1.31	0.05	1.71	0.04	0.82	0.05
ATP2B2	1.63	0.00	2.03	0.03	1.14	0.00
ATP6V1A	0.35	0.00	0.82	0.01	0.86	0.02
BDH1	1.26	0.02	0.73	0.01	0.89	0.03
BTBD1	0.4	0.01	0.8	0.03	0.92	0.03
BTG2	1.53	0.02	1.56	0.00	1.35	0.01
C9orf3	0.45	0.00	0.6	0.02	0.9	0.02
CACNA1D	0.33	0.02	0.65	0.00	0.73	0.00
CACNA2D1	1.37	0.01	1.49	0.04	0.73	0.01
CADM1	0.41	0.01	1.23	0.02	0.82	0.03
CAPZA1	0.56	0.00	1.5	0.04	1.1	0.02
CASQ2	1.53	0.00	1.85	0.06	1.12	0.04
CBFA2T2	1.43	0.02	1.79	0.00	0.87	0.03
CCL21	1.46	0.01	0.82	0.02	1.3	0.01
CD2	1.47	0.00	1.16	0.06	1.11	0.02
CDC14B	1.35	0.00	0.77	0.01	0.84	0.00

CDC42EP3	0.43	0.00	1.39	0.03	0.77	0.01
CDK18	1.5	0.01	1.55	0.02	0.9	0.04
CDK5RAP2	1.35	0.01	1.16	0.06	1.11	0.04
CDKL5	1.37	0.01	0.59	0.01	0.79	0.00
CDS2	0.61	0.00	1.28	0.06	0.85	0.01
CENPC	0.6	0.00	0.71	0.03	0.79	0.00
CENPI	1.3	0.02	2.29	0.01	0.79	0.03
CIB2	1.46	0.01	1.49	0.03	0.87	0.01
CITED2	0.52	0.00	0.78	0.05	0.9	0.05
CLCN4	0.46	0.02	0.67	0.00	0.77	0.01
CLGN	0.16	0.00	0.68	0.01	0.68	0.02
CLN5	0.48	0.00	0.75	0.01	0.89	0.02
CORO2A	1.27	0.04	0.57	0.04	1.22	0.03
COX11	0.23	0.00	1.22	0.02	0.9	0.04
COX5B	0.6	0.00	0.51	0.00	0.84	0.00
CPD	0.32	0.00	0.69	0.02	0.83	0.01
CRLF3	0.56	0.00	1.3	0.03	1.14	0.01
CRYZL1	0.63	0.02	1.35	0.04	0.91	0.04
CTNNA2	0.79	0.05	0.57	0.00	0.74	0.02
CYB5R4	0.38	0.01	0.77	0.04	0.88	0.03
CYHR1	1.4	0.01	1.44	0.01	0.82	0.00
CYP2U1	1.28	0.04	0.81	0.03	0.73	0.00
CYP4F2	1.53	0.00	0.33	0.00	1.19	0.04
DBP	1.33	0.02	1.33	0.04	0.87	0.01
DCC	1.53	0.00	0.7	0.02	0.83	0.03
DCN	0.64	0.04	0.58	0.03	1.6	0.00
DDX10	0.78	0.05	0.82	0.06	1.13	0.05
DDX18	0.38	0.00	1.42	0.00	1.16	0.01
DHRS2	0.24	0.01	0.63	0.03	0.69	0.00
DHX40	0.45	0.01	0.78	0.01	0.9	0.02
DNASE2	1.3	0.01	1.25	0.02	0.87	0.02
DOCK10	0.73	0.02	0.77	0.01	1.39	0.01
DOCK5	1.29	0.00	0.66	0.03	0.74	0.01
DUOX1	1.65	0.00	0.82	0.04	1.11	0.03
DVL2	1.55	0.01	1.24	0.04	0.9	0.01
DYNC2LI1	0.66	0.05	1.49	0.06	0.89	0.02
EDN3	1.3	0.01	0.45	0.00	0.67	0.00
EEF1D	1.41	0.00	1.31	0.00	1.13	0.02
EHD1	1.66	0.00	0.85	0.02	1.1	0.05
EIF2AK2	0.52	0.01	0.78	0.03	0.9	0.05
ENTPD3	0.32	0.00	0.78	0.01	0.58	0.00
EPB41L5	1.53	0.00	0.55	0.01	0.89	0.01
ERAP1	0.78	0.01	1.49	0.02	1.18	0.05
ERGIC3	1.28	0.01	0.76	0.05	0.91	0.05
ERO1B	0.17	0.00	0.81	0.03	0.62	0.00
ETV5	1.31	0.05	0.74	0.01	0.87	0.02

ETV7	1.54	0.01	1.72	0.04	1.09	0.03
EXTL1	1.48	0.00	0.83	0.02	1.11	0.04
F5	1.6	0.05	0.05	0.67	0.05	0.67
FAM222B	1.18	0.05	1.35	0.01	0.93	0.05
FBXO7	0.66	0.01	0.76	0.02	0.85	0.00
FECH	0.69	0.03	0.7	0.05	0.87	0.02
FGD6	0.74	0.05	0.83	0.03	1.19	0.03
FLII	0.61	0.02	1.21	0.05	1.2	0.01
FOXJ3	0.67	0.01	0.83	0.03	0.89	0.02
FOXL1	1.5	0.01	0.81	0.06	1.19	0.00
FOXN3	1.69	0.01	1.39	0.00	0.85	0.01
FRMD4B	1.33	0.01	1.43	0.02	1.17	0.04
GABRG2	1.42	0.03	1.46	0.02	0.7	0.02
GART	1.25	0.03	1.38	0.01	1.18	0.03
GLT8D1	0.26	0.00	1.14	0.05	0.87	0.02
GM2A	1.24	0.04	0.64	0.03	0.8	0.02
GNAQ	1.52	0.00	0.77	0.01	0.91	0.05
GNAS	1.34	0.01	1.61	0.03	0.82	0.01
GNG3	1.23	0.05	0.81	0.03	1.12	0.03
GNRHR	1.22	0.04	0.66	0.02	1.13	0.02
GNS	0.43	0.00	1.17	0.02	0.82	0.01
GOLGA5	0.25	0.00	0.73	0.02	0.86	0.01
GRAMD3	0.5	0.01	0.66	0.02	0.74	0.00
GRPEL1	0.58	0.02	0.72	0.01	1.1	0.03
GSN	1.31	0.00	0.55	0.01	0.85	0.02
GSPT1	0.34	0.00	1.21	0.02	1.09	0.03
GTF3C3	0.4	0.00	0.55	0.01	0.91	0.02
HADH	0.18	0.00	0.63	0.00	0.64	0.00
HEATR1	0.6	0.02	1.29	0.05	1.23	0.00
HGF	1.46	0.01	3.38	0.00	1.44	0.01
HIPK2	1.99	0.01	0.59	0.05	0.87	0.03
HLF	0.68	0.03	0.67	0.01	0.73	0.02
HMGN5	1.21	0.03	0.5	0.01	0.75	0.00
HNF4G	1.32	0.02	1.43	0.04	1.26	0.03
HSD11B2	1.49	0.00	1.73	0.04	1.15	0.04
IAPP	0.09	0.00	0.67	0.03	0.71	0.02
IKZF4	1.34	0.05	1.24	0.01	0.87	0.01
IL11	1.36	0.03	1.41	0.01	1.39	0.04
IL12RB1	1.28	0.01	1.21	0.01	1.1	0.03
INA	0.39	0.00	0.76	0.01	0.77	0.04
INTS6	1.66	0.02	0.77	0.04	1.12	0.01
ITGAX	1.52	0.02	0.67	0.05	1.14	0.04
KAT2B	0.53	0.04	0.69	0.04	0.77	0.01
KIAA1324	0.6	0.03	1.15	0.05	0.75	0.03
KLF12	1.39	0.01	1.91	0.01	0.79	0.00
KLHL1	1.32	0.03	0.66	0.04	0.78	0.04

KLHL18	0.61	0.00	1.26	0.04	0.9	0.00
KLHL22	1.18	0.04	1.36	0.06	0.92	0.02
KLHL24	1.93	0.01	1.72	0.03	0.87	0.03
LAPTM5	1.26	0.02	0.79	0.01	1.31	0.01
LMAN1	1.41	0.01	0.74	0.01	0.9	0.05
LMBRD1	0.25	0.00	1.71	0.05	0.85	0.01
LPAR3	1.39	0.02	0.49	0.00	1.35	0.06
LPXN	0.62	0.00	1.78	0.03	1.14	0.04
LRFN3	1.54	0.01	1.43	0.02	0.89	0.02
LZTFL1	0.37	0.00	0.73	0.04	0.9	0.04
MADCAM1	1.4	0.04	1.3	0.02	1.11	0.01
MAGED2	0.58	0.00	0.76	0.04	0.83	0.02
MAK16	0.53	0.00	0.74	0.00	1.22	0.00
MAML3	1.24	0.03	0.66	0.01	0.82	0.01
MAP2K6	1.29	0.01	0.74	0.06	0.76	0.00
MAP3K2	1.33	0.02	0.82	0.03	0.87	0.04
MCL1	1.46	0.01	1.37	0.05	1.18	0.01
MDM1	0.74	0.02	0.73	0.03	0.87	0.05
MET	1.52	0.01	2.07	0.02	1.47	0.01
MIA2	1.19	0.03	0.77	0.02	0.74	0.00
MPHOSPH9	0.63	0.01	1.68	0.00	0.89	0.04
MPZL1	1.3	0.02	1.51	0.02	1.1	0.01
MREG	0.53	0.05	0.77	0.01	1.16	0.02
MRPL3	0.32	0.00	0.84	0.06	1.07	0.04
MTUS2	0.41	0.00	0.73	0.00	0.8	0.03
MUSK	1.79	0.00	0.68	0.03	1.1	0.02
MYOG	1.49	0.01	1.41	0.00	1.11	0.05
NBEA	0.33	0.01	0.78	0.00	0.82	0.04
NCS1	1.4	0.05	1.38	0.02	1.18	0.03
NDUFA10	0.61	0.01	0.73	0.03	0.94	0.04
NEBL	0.35	0.00	0.57	0.00	0.79	0.00
NENF	1.76	0.00	1.5	0.00	0.87	0.04
NFATC2IP	1.42	0.02	1.59	0.00	1.11	0.01
NIPSNAP1	1.57	0.03	0.83	0.06	0.86	0.04
NKX2-2	0.28	0.01	0.75	0.06	0.74	0.02
NPC1	0.53	0.05	0.76	0.02	1.12	0.02
NR0B1	0.38	0.01	0.65	0.04	0.61	0.01
NR6A1	1.42	0.00	1.61	0.01	1.1	0.05
NRCAM	0.35	0.00	0.83	0.06	0.82	0.03
NRDE2	1.17	0.05	0.5	0.00	0.9	0.02
NRG1	1.41	0.03	1.68	0.06	1.09	0.01
NXPE3	0.85	0.04	0.59	0.01	0.81	0.01
OLIG2	1.3	0.02	1.24	0.03	1.12	0.02
OSBPL8	0.32	0.00	1.33	0.05	0.87	0.01
OXR1	0.75	0.03	0.59	0.00	1.18	0.05
PADI2	1.51	0.01	1.41	0.05	1.08	0.05

PCDH7	1.33	0.05	1.53	0.02	0.84	0.03
PCGF2	1.57	0.01	1.47	0.01	0.92	0.02
PCMT1	0.41	0.00	0.87	0.04	0.91	0.04
PDCD11	1.43	0.00	1.19	0.03	1.19	0.02
PDGFRA	1.26	0.01	1.27	0.03	1.74	0.00
PDLIM4	1.2	0.02	1.99	0.00	1.24	0.00
PDPN	1.36	0.01	1.76	0.02	1.25	0.00
PDX1	1.51	0.01	1.58	0.00	0.82	0.01
PEBP1	0.49	0.00	0.8	0.01	0.83	0.00
PEX19	0.72	0.04	0.85	0.04	0.91	0.04
PFKFB2	0.73	0.05	0.58	0.01	0.56	0.00
PFKFB4	1.25	0.05	0.8	0.03	0.88	0.01
PGRMC1	0.34	0.00	0.73	0.00	0.89	0.02
PHACTR2	0.39	0.01	0.81	0.03	0.87	0.05
PHF7	1.33	0.01	0.74	0.02	0.84	0.02
PIAS2	1.14	0.05	0.47	0.04	0.88	0.02
PID1	0.79	0.02	1.66	0.00	1.23	0.01
PIGA	0.45	0.01	0.49	0.00	0.86	0.03
PLAUR	1.46	0.02	0.8	0.01	1.27	0.01
PLEKHA4	1.7	0.00	1.65	0.04	1.1	0.01
PMAIP1	0.59	0.02	1.5	0.03	1.25	0.04
PNMA2	0.13	0.00	0.78	0.00	0.72	0.01
POLR1E	1.32	0.05	1.47	0.02	1.19	0.03
POU2F3	1.33	0.05	1.42	0.02	1.09	0.05
PPIF	0.58	0.03	0.83	0.01	1.12	0.04
PPP1CA	0.67	0.03	1.32	0.01	1.1	0.04
PPP1R13L	1.74	0.00	0.41	0.00	0.55	0.00
PPP1R1A	0.46	0.04	1.41	0.04	1.14	0.01
PPP2R2A	0.65	0.03	0.75	0.02	1.1	0.01
PRKCH	0.45	0.00	0.8	0.02	0.84	0.03
PRKRA	0.3	0.00	0.69	0.04	0.9	0.03
PSAP	0.38	0.00	1.18	0.04	0.89	0.03
PSIP1	0.48	0.02	0.77	0.01	0.85	0.01
PTCH1	0.51	0.05	0.62	0.01	0.79	0.00
PTENP1///PTEN	0.5	0.00	0.71	0.04	0.9	0.02
PTGES	1.29	0.03	1.3	0.01	1.44	0.00
PTPN3	0.48	0.00	0.59	0.00	0.83	0.01
PTPRC	1.35	0.01	1.15	0.04	1.43	0.00
PYY	1.5	0.02	1.47	0.01	0.89	0.01
RAB2A	0.25	0.00	0.81	0.06	0.89	0.02
RAB30	1.35	0.01	0.65	0.02	0.85	0.03
RAC2	1.59	0.00	1.39	0.01	1.15	0.01
RANBP1	1.39	0.01	2.03	0.04	1.1	0.02
RANBP9	0.48	0.01	1.91	0.02	0.89	0.01
RAP1GAP2	0.52	0.04	0.77	0.02	0.8	0.03

RCAN2	0.41	0.04	0.61	0.00	0.76	0.01
RCBTB1	0.45	0.02	0.8	0.04	0.8	0.00
RELB	1.41	0.02	2.19	0.01	1.15	0.02
RHOBTB3	0.3	0.00	0.61	0.00	0.77	0.00
RNF6	0.18	0.00	0.73	0.00	0.81	0.00
RPS19	0.71	0.01	1.41	0.00	1.13	0.04
RRAGD	0.21	0.00	0.74	0.00	0.67	0.00
RRP12	1.27	0.02	1.36	0.01	1.19	0.01
RSL1D1	0.47	0.00	1.74	0.02	1.13	0.03
RWDD2A	0.48	0.00	0.81	0.01	0.83	0.01
RYK	0.59	0.01	1.38	0.03	1.16	0.01
SCAMP1	0.41	0.00	0.82	0.04	0.85	0.02
SCD	0.26	0.01	0.66	0.01	0.66	0.00
SDC1	1.41	0.02	1.29	0.02	1.21	0.01
SDS	1.25	0.03	1.29	0.04	1.29	0.01
SEC24D	0.34	0.00	0.83	0.04	0.85	0.01
SEMA3A	1.33	0.03	0.49	0.01	1.43	0.00
SEMA3C	1.35	0.02	1.52	0.03	1.24	0.02
SEMA5A	0.5	0.00	0.68	0.05	0.81	0.02
SERHL2///SERHL	1.48	0.00	0.8	0.04	1.13	0.00
SERPINB1	0.59	0.02	1.42	0.02	1.27	0.02
SGSM2	1.57	0.01	2.66	0.00	0.88	0.03
SH2D3A	1.61	0.00	1.36	0.06	1.22	0.00
SH3BGRL	0.27	0.01	0.82	0.00	0.81	0.01
SH3BP2	1.56	0.00	1.17	0.03	1.11	0.04
SIRPA	1.56	0.00	2.26	0.00	1.15	0.01
SIRT2	1.64	0.00	1.34	0.02	0.9	0.01
SLAMF7	1.51	0.01	1.3	0.02	1.21	0.03
SLC1A2	1.37	0.01	1.33	0.04	1.31	0.02
SLC25A13	0.54	0.04	0.76	0.01	1.1	0.05
SLC25A36	0.44	0.02	1.35	0.00	0.86	0.02
SLC7A11	0.52	0.00	2.02	0.05	1.34	0.01
SLC17A5	0.7	0.03	0.77	0.04	0.82	0.01
SLC19A1	1.43	0.02	0.25	0.01	1.09	0.03
SLC29A3	1.42	0.02	1.35	0.00	1.08	0.02
SLC30A9	0.42	0.00	0.85	0.02	0.91	0.05
SMAD9	1.34	0.02	0.65	0.03	0.7	0.00
SMURF1	1.3	0.03	2.23	0.01	1.14	0.04
SNTB1	1.33	0.00	0.84	0.04	1.19	0.02
SNX2	0.26	0.00	0.47	0.05	0.89	0.04
SNX13	1.45	0.00	0.43	0.02	0.89	0.01
SPTBN4	1.37	0.01	1.5	0.02	0.91	0.02
SRD5A1	0.51	0.03	0.61	0.01	0.79	0.00
SRSF1	1.29	0.02	1.79	0.02	1.12	0.05
STK17A	0.52	0.05	1.54	0.01	1.25	0.02

STMN3	1.21	0.03	1.22	0.03	0.9	0.05
SYDE1	1.38	0.00	2.73	0.00	1.09	0.05
SYNDIG1	1.36	0.03	1.58	0.01	1.15	0.05
SYT13	1.54	0.01	1.3	0.04	0.66	0.00
40057	1.61	0.00	1.37	0.02	1.19	0.01
TACSTD2	1.34	0.03	1.44	0.01	1.27	0.04
TAF1D	0.5	0.00	1.46	0.03	1.31	0.01
TBC1D19	0.7	0.05	1.33	0.01	0.9	0.05
TBC1D5	1.36	0.01	0.81	0.04	0.85	0.02
TCEA2	0.72	0.03	0.53	0.05	0.91	0.02
TEAD4	1.53	0.00	1.93	0.00	1.16	0.02
TGFBR3	0.49	0.02	0.78	0.01	0.72	0.00
TGM2	1.75	0.00	1.37	0.03	1.31	0.02
THBS2	1.62	0.01	2.43	0.04	1.54	0.01
THRA	1.45	0.00	1.18	0.04	0.84	0.01
TLK1	0.29	0.00	1.95	0.03	0.87	0.00
TMEM39A	0.72	0.04	0.73	0.01	1.12	0.05
TMEM59	0.49	0.00	0.82	0.06	0.88	0.02
TMPO	1.28	0.05	0.78	0.05	1.18	0.01
TNFRSF10B	1.54	0.01	1.79	0.01	1.23	0.02
TNFRSF14	1.68	0.00	1.29	0.04	1.09	0.04
TNS2	1.32	0.04	1.62	0.00	0.9	0.05
TRADD	1.6	0.00	1.34	0.01	1.14	0.04
TRAK1	0.59	0.01	0.81	0.04	0.83	0.02
TRAPPC13	0.54	0.01	0.6	0.05	0.87	0.02
TREML2	1.36	0.04	0.83	0.04	1.09	0.04
TRIM37	0.46	0.00	0.81	0.00	0.81	0.00
TRIP10	1.53	0.01	1.79	0.00	1.16	0.03
TRPC1	0.45	0.03	0.68	0.04	0.83	0.04
TRPM3	1.22	0.02	1.41	0.06	0.78	0.02
TSN	0.34	0.00	0.74	0.05	0.91	0.01
TTLL4	1.3	0.00	0.66	0.02	1.15	0.01
TYRO3	1.4	0.03	0.7	0.04	1.15	0.04
UBL3	0.42	0.00	0.53	0.00	0.87	0.02
UFSP2	0.45	0.01	0.82	0.01	0.84	0.00
UNC93B1	1.56	0.00	1.89	0.00	1.15	0.03
VPS33A	1.17	0.05	1.95	0.03	0.89	0.02
VRK3	1.34	0.01	8.19	0.00	1.12	0.02
WASL	1.53	0.01	0.78	0.01	0.88	0.01
WDR1	1.31	0.01	1.43	0.01	1.12	0.03
WISP1	1.33	0.04	2.62	0.01	1.18	0.01
WNT4	0.4	0.03	1.51	0.05	0.76	0.00
WT1	1.28	0.05	2.43	0.00	1.1	0.05
YIPF2	1.45	0.00	1.38	0.05	0.9	0.05
ZFAND6	0.49	0.01	0.86	0.02	0.9	0.04
ZMAT4	1.32	0.05	0.65	0.01	0.88	0.04

ZNF148	0.54	0.00	0.85	0.04	0.92	0.04
ZNF280B	1.3	0.01	0.39	0.01	0.82	0.02
ZNF358	1.9	0.00	1.29	0.01	0.9	0.01

Table S2. Up-regulated differentially expressed genes of diabetic pancreatic tissue (Group: 1).

	**Pancreas** GSE25724		**Pancreas** GSE20966		**Pancreas** GSE38642	
Gene Symbol	FCE	p-Value	FCE	p-Value	FCE	p-Value
ABCA6	1.31	0.01	1.40	0.04	1.15	0.01
ADAM28	1.42	0.00	1.82	0.03	1.51	0.00
ADD2	1.46	0.01	2.75	0.00	1.13	0.04
ALDH1A2	1.51	0.00	1.66	0.06	1.28	0.02
ATP2B2	1.63	0.00	2.03	0.03	1.14	0.00
BTG2	1.53	0.02	1.56	0.00	1.35	0.01
CASQ2	1.53	0.00	1.85	0.06	1.12	0.04
CD2	1.47	0.00	1.16	0.06	1.11	0.02
CDK5RAP2	1.35	0.01	1.16	0.06	1.11	0.04
EEF1D	1.41	0.00	1.31	0.00	1.13	0.02
ETV7	1.54	0.01	1.72	0.04	1.09	0.03
FRMD4B	1.33	0.01	1.43	0.02	1.17	0.04
GART	1.25	0.03	1.38	0.01	1.18	0.03
HGF	1.46	0.01	3.38	0.00	1.44	0.01
HNF4G	1.32	0.02	1.43	0.04	1.26	0.03
HSD11B2	1.49	0.00	1.73	0.04	1.15	0.04
IL11	1.36	0.03	1.41	0.01	1.39	0.04
IL12RB1	1.28	0.01	1.21	0.01	1.10	0.03
MADCAM1	1.40	0.04	1.30	0.02	1.11	0.01
MCL1	1.46	0.01	1.37	0.05	1.18	0.01
MET	1.52	0.01	2.07	0.02	1.47	0.01
MPZL1	1.30	0.02	1.51	0.02	1.10	0.01
MYOG	1.49	0.01	1.41	0.00	1.11	0.05
NCS1	1.40	0.05	1.38	0.02	1.18	0.03
NFATC2IP	1.42	0.02	1.59	0.00	1.11	0.01
NR6A1	1.42	0.00	1.61	0.01	1.10	0.05
NRG1	1.41	0.03	1.68	0.06	1.09	0.01
OLIG2	1.30	0.02	1.24	0.03	1.12	0.02
PADI2	1.51	0.01	1.41	0.05	1.08	0.05
PDCD11	1.43	0.00	1.19	0.03	1.19	0.02
PDGFRA	1.26	0.01	1.27	0.03	1.74	0.00
PDLIM4	1.20	0.02	1.99	0.00	1.24	0.00
PDPN	1.36	0.01	1.76	0.02	1.25	0.00
PLEKHA4	1.70	0.00	1.65	0.04	1.10	0.01
POLR1E	1.32	0.05	1.47	0.02	1.19	0.03
POU2F3	1.33	0.05	1.42	0.02	1.09	0.05
PTGES	1.29	0.03	1.30	0.01	1.44	0.00
PTPRC	1.35	0.01	1.15	0.04	1.43	0.00
RAC2	1.59	0.00	1.39	0.01	1.15	0.01
RANBP1	1.39	0.01	2.03	0.04	1.10	0.02
RELB	1.41	0.02	2.19	0.01	1.15	0.02

RRP12	1.27	0.02	1.36	0.01	1.19	0.01
SDC1	1.41	0.02	1.29	0.02	1.21	0.01
SDS	1.25	0.03	1.29	0.04	1.29	0.01
SEMA3C	1.35	0.02	1.52	0.03	1.24	0.02
SH2D3A	1.61	0.00	1.36	0.06	1.22	0.00
SH3BP2	1.56	0.00	1.17	0.03	1.11	0.04
SIRPA	1.56	0.00	2.26	0.00	1.15	0.01
SLAMF7	1.51	0.01	1.30	0.02	1.21	0.03
SLC1A2	1.37	0.01	1.33	0.04	1.31	0.02
SLC29A3	1.42	0.02	1.35	0.00	1.08	0.02
SMURF1	1.30	0.03	2.23	0.01	1.14	0.04
SRSF1	1.29	0.02	1.79	0.02	1.12	0.05
SYDE1	1.38	0.00	2.73	0.00	1.09	0.05
SYNDIG1	1.36	0.03	1.58	0.01	1.15	0.05
40057	1.61	0.00	1.37	0.02	1.19	0.01
TACSTD2	1.34	0.03	1.44	0.01	1.27	0.04
TEAD4	1.53	0.00	1.93	0.00	1.16	0.02
TGM2	1.75	0.00	1.37	0.03	1.31	0.02
THBS2	1.62	0.01	2.43	0.04	1.54	0.01
TNFRSF10B	1.54	0.01	1.79	0.01	1.23	0.02
TNFRSF14	1.68	0.00	1.29	0.04	1.09	0.04
TRADD	1.60	0.00	1.34	0.01	1.14	0.04
TRIP10	1.53	0.01	1.79	0.00	1.16	0.03
UNC93B1	1.56	0.00	1.89	0.00	1.15	0.03
VRK3	1.34	0.01	8.19	0.00	1.12	0.02
WDR1	1.31	0.01	1.43	0.01	1.12	0.03
WISP1	1.33	0.04	2.62	0.01	1.18	0.01
WT1	1.28	0.05	2.43	0.00	1.10	0.05

Table S3. Down-regulated differentially expressed genes of diabetic pancreatic tissue (Group: 1).

	**Pancreas** GSE25724		**Pancreas** GSE20966		**Pancreas** GSE38642	
Gene Symbol	FCE	p-Value	FCE	p-Value	FCE	p-Value
ABAT	0.28	0.00	0.78	0.03	0.78	0.03
ABCC8	0.36	0.01	0.77	0.00	0.54	0.00
ABHD10	0.57	0.00	0.80	0.02	0.74	0.00
ACLY	0.50	0.00	0.55	0.03	0.83	0.04
ADCYAP1	0.27	0.01	0.66	0.03	0.72	0.03
AGTPBP1	0.49	0.01	0.79	0.05	0.83	0.00
AKAP11	0.22	0.00	0.70	0.01	0.86	0.04
APC	0.36	0.00	0.71	0.02	0.82	0.01
APPL2	0.73	0.01	0.74	0.04	0.89	0.01
ARG2	0.20	0.00	0.47	0.02	0.57	0.00
ARHGEF9	0.50	0.02	0.64	0.00	0.74	0.01
ARL1	0.38	0.00	0.80	0.01	0.88	0.01
ATP6V1A	0.35	0.00	0.82	0.01	0.86	0.02
BTBD1	0.40	0.01	0.80	0.03	0.92	0.03
C9orf3	0.45	0.00	0.60	0.02	0.90	0.02
CACNA1D	0.33	0.02	0.65	0.00	0.73	0.00
CENPC	0.60	0.00	0.71	0.03	0.79	0.00
CITED2	0.52	0.00	0.78	0.05	0.90	0.05
CLCN4	0.46	0.02	0.67	0.00	0.77	0.01
CLGN	0.16	0.00	0.68	0.01	0.68	0.02
CLN5	0.48	0.00	0.75	0.01	0.89	0.02
COX11	0.23	0.00	1.22	0.02	0.90	0.04
COX5B	0.60	0.00	0.51	0.00	0.84	0.00
CPD	0.32	0.00	0.69	0.02	0.83	0.01
CTNNA2	0.79	0.05	0.57	0.00	0.74	0.02
CYB5R4	0.38	0.01	0.77	0.04	0.88	0.03
DHRS2	0.24	0.01	0.63	0.03	0.69	0.00
DHX40	0.45	0.01	0.78	0.01	0.90	0.02
EIF2AK2	0.52	0.01	0.78	0.03	0.90	0.05
ENTPD3	0.32	0.00	0.78	0.01	0.58	0.00
ERO1B	0.17	0.00	0.81	0.03	0.62	0.00
FBXO7	0.66	0.01	0.76	0.02	0.85	0.00
FECH	0.69	0.03	0.70	0.05	0.87	0.02
FOXJ3	0.67	0.01	0.83	0.03	0.89	0.02
GOLGA5	0.25	0.00	0.73	0.02	0.86	0.01
GRAMD3	0.50	0.01	0.66	0.02	0.74	0.00
GTF3C3	0.40	0.00	0.55	0.01	0.91	0.02
HADH	0.18	0.00	0.63	0.00	0.64	0.00
HLF	0.68	0.03	0.67	0.01	0.73	0.02
IAPP	0.09	0.00	0.67	0.03	0.71	0.02
INA	0.39	0.00	0.76	0.01	0.77	0.04

KAT2B	0.53	0.04	0.69	0.04	0.77	0.01
LZTFL1	0.37	0.00	0.73	0.04	0.90	0.04
MAGED2	0.58	0.00	0.76	0.04	0.83	0.02
MDM1	0.74	0.02	0.73	0.03	0.87	0.05
MTUS2	0.41	0.00	0.73	0.00	0.80	0.03
NBEA	0.33	0.01	0.78	0.00	0.82	0.04
NDUFA10	0.61	0.01	0.73	0.03	0.94	0.04
NEBL	0.35	0.00	0.57	0.00	0.79	0.00
NKX2-2	0.28	0.01	0.75	0.06	0.74	0.02
NR0B1	0.38	0.01	0.65	0.04	0.61	0.01
NRCAM	0.35	0.00	0.83	0.06	0.82	0.03
NXPE3	0.85	0.04	0.59	0.01	0.81	0.01
PCMT1	0.41	0.00	0.87	0.04	0.91	0.04
PEBP1	0.49	0.00	0.80	0.01	0.83	0.00
PEX19	0.72	0.04	0.85	0.04	0.91	0.04
PFKFB2	0.73	0.05	0.58	0.01	0.56	0.00
PGRMC1	0.34	0.00	0.73	0.00	0.89	0.02
PHACTR2	0.39	0.01	0.81	0.03	0.87	0.05
PIGA	0.45	0.01	0.49	0.00	0.86	0.03
PNMA2	0.13	0.00	0.78	0.00	0.72	0.01
PRKCH	0.45	0.00	0.80	0.02	0.84	0.03
PRKRA	0.30	0.00	0.69	0.04	0.90	0.03
PSIP1	0.48	0.02	0.77	0.01	0.85	0.01
PTCH1	0.51	0.05	0.62	0.01	0.79	0.00
PTENP1///PTEN	0.50	0.00	0.71	0.04	0.90	0.02
PTPN3	0.48	0.00	0.59	0.00	0.83	0.01
RAB2A	0.25	0.00	0.81	0.06	0.89	0.02
RAP1GAP2	0.52	0.04	0.77	0.02	0.80	0.03
RCAN2	0.41	0.04	0.61	0.00	0.76	0.01
RCBTB1	0.45	0.02	0.80	0.04	0.80	0.00
RHOBTB3	0.30	0.00	0.61	0.00	0.77	0.00
RNF6	0.18	0.00	0.73	0.00	0.81	0.00
RRAGD	0.21	0.00	0.74	0.00	0.67	0.00
RWDD2A	0.48	0.00	0.81	0.01	0.83	0.01
SCAMP1	0.41	0.00	0.82	0.04	0.85	0.02
SCD	0.26	0.01	0.66	0.01	0.66	0.00
SEC24D	0.34	0.00	0.83	0.04	0.85	0.01
SEMA5A	0.50	0.00	0.68	0.05	0.81	0.02
SH3BGRL	0.27	0.01	0.82	0.00	0.81	0.01
SLC17A5	0.70	0.03	0.77	0.04	0.82	0.01
SLC30A9	0.42	0.00	0.85	0.02	0.91	0.05
SNX2	0.26	0.00	0.47	0.05	0.89	0.04
SRD5A1	0.51	0.03	0.61	0.01	0.79	0.00
TCEA2	0.72	0.03	0.53	0.05	0.91	0.02
TGFBR3	0.49	0.02	0.78	0.01	0.72	0.00
TMEM59	0.49	0.00	0.82	0.06	0.88	0.02

TRAK1	0.59	0.01	0.81	0.04	0.83	0.02
TRAPPC13	0.54	0.01	0.60	0.05	0.87	0.02
TRIM37	0.46	0.00	0.81	0.00	0.81	0.00
TRPC1	0.45	0.03	0.68	0.04	0.83	0.04
TSN	0.34	0.00	0.74	0.05	0.91	0.01
UBL3	0.42	0.00	0.53	0.00	0.87	0.02
UFSP2	0.45	0.01	0.82	0.01	0.84	0.00
ZFAND6	0.49	0.01	0.86	0.02	0.90	0.04
ZNF148	0.54	0.00	0.85	0.04	0.92	0.04

Table S4: Complete Common differentially expressed genes of diabetic pancreatic tissue and diabetic heart tissue (Group: 2).

	Pancreas (GSE25724)		Pancreas (GSE20966)		Pancreas (GSE38642)		Heart (GSE26887)	
Gene Symbol	FCE	p-Value	FCE	p-Value	FCE	p-Value	FCE	p-Value
ABCA6	1.31	0.01	1.40	0.04	1.15	0.01	1.54	0.04
ACLY	0.50	0.00	0.55	0.03	0.83	0.04	0.73	0.03
ADAM28	1.42	0.00	1.82	0.03	1.51	0.00	1.56	0.02
AKAP11	0.22	0.00	0.70	0.01	0.86	0.04	1.37	0.01
CBFA2T2	1.43	0.02	1.79	0.00	0.87	0.03	1.21	0.02
CDK18	1.50	0.01	1.55	0.02	0.90	0.04	1.66	0.01
CDK5RAP2	1.35	0.01	1.16	0.06	1.11	0.04	1.31	0.03
CDS2	0.61	0.00	1.28	0.06	0.85	0.01	1.43	0.03
CENPC	0.60	0.00	0.71	0.03	0.79	0.00	1.65	0.00
CITED2	0.52	0.00	0.78	0.05	0.90	0.05	0.59	0.00
CYP2U1	1.28	0.04	0.81	0.03	0.73	0.00	1.24	0.02
DOCK10	0.73	0.02	0.77	0.01	1.39	0.01	0.68	0.02
DOCK5	1.29	0.00	0.66	0.03	0.74	0.01	1.23	0.04
DYNC2LI1	0.66	0.05	1.49	0.06	0.89	0.02	0.84	0.03
EEF1D	1.41	0.00	1.31	0.00	1.13	0.02	1.70	0.00
ERAP1	0.78	0.01	1.49	0.02	1.18	0.05	1.34	0.03
FGD6	0.74	0.05	0.83	0.03	1.19	0.03	1.41	0.02
FLII	0.61	0.02	1.21	0.05	1.20	0.01	0.63	0.00
FRMD4B	1.33	0.01	1.43	0.02	1.17	0.04	1.67	0.01
GART	1.25	0.03	1.38	0.01	1.18	0.03	0.70	0.01
GNAQ	1.52	0.00	0.77	0.01	0.91	0.05	1.52	0.03
HADH	0.18	0.00	0.63	0.00	0.64	0.00	1.63	0.06
HIPK2	1.99	0.01	0.59	0.05	0.87	0.03	0.69	0.04
IAPP	0.09	0.00	0.67	0.03	0.71	0.02	1.46	0.02
ITGAX	1.52	0.02	0.67	0.05	1.14	0.04	0.73	0.04
KLHL24	1.93	0.01	1.36	0.06	0.87	0.03	1.64	0.04
LAPTM5	1.26	0.02	1.72	0.03	1.31	0.01	0.69	0.05
MAGED2	0.58	0.00	0.76	0.04	0.83	0.02	1.24	0.04
MAML3	1.24	0.03	0.66	0.01	0.82	0.01	1.49	0.00
MRPL3	0.32	0.00	0.84	0.06	1.07	0.04	0.70	0.01
MUSK	1.79	0.00	0.68	0.03	1.10	0.02	1.96	0.00
NEBL	0.35	0.00	0.57	0.00	0.79	0.00	1.34	0.04
NPC1	0.53	0.05	0.76	0.02	1.12	0.02	0.65	0.04
NRG1	1.41	0.03	1.68	0.06	1.09	0.01	2.45	0.00
OXR1	0.75	0.03	0.59	0.00	1.18	0.05	1.38	0.01
PCDH7	1.33	0.05	1.53	0.02	0.84	0.03	0.38	0.00
PDCD11	1.43	0.00	1.19	0.03	1.19	0.02	0.76	0.03
PDPN	1.36	0.01	1.76	0.02	1.25	0.00	0.51	0.00
PFKFB2	0.73	0.05	0.58	0.01	0.56	0.00	1.89	0.00
PIGA	0.45	0.01	0.49	0.00	0.86	0.03	0.67	0.03

PLAUR	1.46	0.02	0.80	0.01	1.27	0.01	0.71	0.05
PLEKHA4	1.70	0.00	1.65	0.04	1.10	0.01	0.81	0.06
PPP1CA	0.67	0.03	1.32	0.01	1.10	0.04	0.77	0.04
PPP1R13L	1.74	0.00	0.41	0.00	0.55	0.00	0.73	0.01
PPP1R1A	0.46	0.04	1.41	0.04	1.14	0.01	1.27	0.02
PRKRA	0.30	0.00	0.69	0.04	0.90	0.03	0.77	0.02
PSIP1	0.48	0.02	0.77	0.01	0.85	0.01	1.30	0.02
PTCH1	0.51	0.05	0.62	0.01	0.79	0.00	1.35	0.01
RAB30	1.35	0.01	0.65	0.02	0.85	0.03	1.41	0.03
RANBP1	1.39	0.01	2.03	0.04	1.10	0.02	0.78	0.04
RELB	1.41	0.02	2.19	0.01	1.15	0.02	0.81	0.03
RRP12	1.27	0.02	1.36	0.01	1.19	0.01	0.61	0.01
SEMA3A	1.33	0.03	0.49	0.01	1.43	0.00	1.71	0.05
SEMA3C	1.35	0.02	1.52	0.03	1.24	0.02	0.67	0.01
SEMA5A	0.50	0.00	0.68	0.05	0.81	0.02	1.56	0.01
SLC1A2	1.37	0.01	1.33	0.04	1.31	0.02	0.79	0.06
SMAD9	1.34	0.02	0.65	0.03	0.70	0.00	1.76	0.00
SNTB1	1.33	0.00	0.84	0.04	1.19	0.02	1.24	0.02
TAF1D	0.50	0.00	1.46	0.03	1.31	0.01	0.68	0.05
TEAD4	1.53	0.00	1.93	0.00	1.16	0.02	0.63	0.00
TGFBR3	0.49	0.02	0.78	0.01	0.72	0.00	0.74	0.02
TGM2	1.75	0.00	1.37	0.03	1.31	0.02	0.72	0.00
TRAK1	0.59	0.01	0.81	0.04	0.83	0.02	1.38	0.02
TRPC1	0.45	0.03	0.68	0.04	0.83	0.04	1.63	0.00
TRPM3	1.22	0.02	1.41	0.06	0.78	0.02	1.19	0.06
WDR1	1.31	0.01	1.43	0.01	1.12	0.03	0.66	0.00
ZNF280B	1.30	0.01	0.39	0.01	0.82	0.02	1.22	0.01

Table S5: Common Up-regulated differentially expressed genes of diabetic pancreatic tissue and diabetic heart tissue (Group: 2).

	**Pancreas (GSE25724)**		**Pancreas (GSE20966)**		**Pancreas (GSE38642)**		**Heart (GSE26887)**	
Gene Symbol	FCE	p-Value	FCE	p-Value	FCE	p-Value	FCE	p-Value
ABCA6	1.31	0.01	1.4	0.04	1.15	0.01	1.54	0.04
ADAM28	1.42	0.00	1.82	0.03	1.51	0.00	1.56	0.02
CDK5RAP2	1.35	0.01	1.16	0.06	1.11	0.04	1.31	0.03
FRMD4B	1.33	0.01	1.43	0.02	1.17	0.04	1.67	0.01
NRG1	1.41	0.03	1.68	0.06	1.09	0.01	2.45	0.00

Table S6: Common Down-regulated differentially expressed genes of diabetic pancreatic tissue and diabetic heart tissue. (Group: 2).

	**Pancreas (GSE25724)**		**Pancreas (GSE20966)**		**Pancreas (GSE38642)**		**Heart (GSE26887)**	
Gene Symbol	FCE	p-Value	FCE	p-Value	FCE	p-Value	FCE	p-Value
ACLY	0.50	0.00	0.55	0.03	0.83	0.04	0.73	0.03
CITED2	0.52	0.00	0.78	0.05	0.90	0.05	0.59	0.00
PIGA	0.45	0.01	0.49	0.00	0.86	0.03	0.67	0.03
PRKRA	0.30	0.00	0.69	0.04	0.90	0.03	0.77	0.02
TGFBR3	0.49	0.02	0.78	0.01	0.72	0.00	0.74	0.02

Table S7: Common Opposite regulated differentially expressed genes of diabetic pancreatic tissue and diabetic heart tissue (Group: 2).

	**Pancreas (GSE25724)**		**Pancreas (GSE20966)**		**Pancreas (GSE38642)**		**Heart (GSE26887)**	
Gene Symbol	FCE	p-Value	FCE	p-Value	FCE	p-Value	FCE	p-Value
AKAP11	0.22	0.00	0.70	0.01	0.86	0.04	1.37	0.01
CENPC	0.60	0.00	0.71	0.03	0.79	0.00	1.65	0.00
EEF1D	1.41	0.00	1.31	0.00	1.13	0.02	1.70	0.00
GART	1.25	0.03	1.38	0.01	1.18	0.03	0.70	0.01
HADH	0.18	0.00	0.63	0.00	0.64	0.00	1.63	0.06
IAPP	0.09	0.00	0.67	0.03	0.71	0.02	1.46	0.02
MAGED2	0.58	0.00	0.76	0.04	0.83	0.02	1.24	0.04
NEBL	0.35	0.00	0.57	0.00	0.79	0.00	1.34	0.04
PDCD11	1.43	0.00	1.19	0.03	1.19	0.02	0.76	0.03
PDPN	1.36	0.01	1.76	0.02	1.25	0.00	0.51	0.00
PFKFB2	0.73	0.05	0.58	0.01	0.56	0.00	1.89	0.00
PLEKHA4	1.70	0.00	1.65	0.04	1.10	0.01	0.81	0.06
PSIP1	0.48	0.02	0.77	0.01	0.85	0.01	1.30	0.02
PTCH1	0.51	0.05	0.62	0.01	0.79	0.00	1.35	0.01
RANBP1	1.39	0.01	2.03	0.04	1.10	0.02	0.78	0.04
RELB	1.41	0.02	2.19	0.01	1.15	0.02	0.81	0.03
RRP12	1.27	0.02	1.36	0.01	1.19	0.01	0.61	0.01
SEMA3C	1.35	0.02	1.52	0.03	1.24	0.02	0.67	0.01
SEMA5A	0.50	0.00	0.68	0.05	0.81	0.02	1.56	0.01
SLC1A2	1.37	0.01	1.33	0.04	1.31	0.02	0.79	0.06
TEAD4	1.53	0.00	1.93	0.00	1.16	0.02	0.63	0.00
TGM2	1.75	0.00	1.37	0.03	1.31	0.02	0.72	0.00
TRAK1	0.59	0.01	0.81	0.04	0.83	0.02	1.38	0.02
TRPC1	0.45	0.03	0.68	0.04	0.83	0.04	1.63	0.00
WDR1	1.31	0.01	1.43	0.01	1.12	0.03	0.66	0.00

Table S8: Complete Common differentially expressed genes of diabetic pancreatic tissue and diabetic liver tissue (Group: 3).

	Pancreas GSE25724		Pancreas GSE20966		Pancreas GSE38642		Liver GSE23343	
**Gene Symbol**	**FCE**	**p-Value**	**FCE**	**p-Value**	**FCE**	**p-Value**	**FCE**	**p-Value**
APC	0.36	0.00	0.71	0.02	0.82	0.01	1.72	0.00
ARHGAP29	0.40	0.02	2.29	0.01	1.17	0.03	2.28	0.01
ARHGEF26	1.27	0.02	0.52	0.05	0.84	0.00	1.49	0.05
ARHGEF9	0.50	0.02	0.64	0.00	0.74	0.01	2.71	0.02
ARL1	0.38	0.00	0.80	0.01	0.88	0.01	1.48	0.04
ASAH1	0.49	0.01	1.18	0.03	0.90	0.04	1.50	0.01
ASB4	1.31	0.05	1.71	0.04	0.82	0.05	0.44	0.04
ATP2B2	1.63	0.00	2.03	0.03	1.14	0.00	1.84	0.06
BTBD1	0.40	0.01	0.80	0.03	0.92	0.03	1.38	0.03
CAPZA1	0.56	0.00	1.50	0.04	1.10	0.02	1.36	0.03
CBFA2T2	1.43	0.02	1.79	0.00	0.87	0.03	0.62	0.01
CDC14B	1.35	0.00	0.77	0.01	0.84	0.00	0.45	0.02
CDC42EP3	0.43	0.00	1.39	0.03	0.77	0.01	2.04	0.02
CDK5RAP2	1.35	0.01	1.55	0.02	0.90	0.04	0.35	0.00
CDKL5	1.37	0.01	0.59	0.01	0.79	0.00	1.45	0.04
CRYZL1	0.63	0.02	1.35	0.04	0.91	0.04	2.22	0.02
DCN	0.64	0.04	0.58	0.03	1.60	0.00	2.38	0.05
DDX18	0.38	0.00	1.42	0.00	1.16	0.01	1.50	0.02
DOCK10	0.73	0.02	0.66	0.03	0.74	0.01	1.81	0.04
EEF1D	1.41	0.00	1.31	0.00	1.13	0.02	0.54	0.05
EPB41L5	1.53	0.00	0.55	0.01	0.89	0.01	1.41	0.05
ERGIC3	1.28	0.01	0.76	0.05	0.91	0.05	0.55	0.04
ETV5	1.31	0.05	0.74	0.01	0.87	0.02	2.34	0.04
FGD6	0.74	0.05	0.83	0.03	1.19	0.03	0.28	0.00
FRMD4B	1.33	0.01	1.43	0.02	1.17	0.04	1.64	0.01
GM2A	1.24	0.04	0.64	0.03	0.80	0.02	2.88	0.02
GNAQ	1.52	0.00	0.77	0.01	0.91	0.05	1.58	0.02
GNAS	1.34	0.01	1.61	0.03	0.82	0.01	1.63	0.06
GSPT1	0.34	0.00	1.21	0.02	1.09	0.03	1.38	0.03
GTF3C3	0.40	0.00	0.55	0.01	0.91	0.02	0.35	0.03
HGF	1.46	0.01	3.38	0.00	1.44	0.01	1.77	0.01
HMGN5	1.21	0.03	0.50	0.01	0.75	0.00	1.71	0.05
HSD11B2	1.49	0.00	1.73	0.04	1.15	0.04	0.73	0.03
KLHL22	1.18	0.04	1.26	0.04	0.92	0.02	1.81	0.05
LMBRD1	0.25	0.00	0.74	0.01	0.85	0.01	1.62	0.04
MAK16	0.53	0.00	0.74	0.00	1.22	0.00	1.44	0.03
MAML3	1.24	0.03	0.66	0.01	0.82	0.01	1.94	0.04
MAP3K2	1.33	0.02	0.82	0.03	0.87	0.04	1.88	0.00
MCL1	1.46	0.01	1.37	0.05	1.18	0.01	1.42	0.03
MPHOSPH9	0.63	0.01	1.68	0.00	0.89	0.04	1.90	0.02
NBEA	0.33	0.01	0.78	0.00	0.82	0.04	1.44	0.06

NPC1	0.53	0.05	0.76	0.02	1.12	0.02	0.49	0.03
NR6A1	1.42	0.00	1.61	0.01	1.10	0.05	2.55	0.00
NRG1	1.41	0.03	1.68	0.06	1.09	0.01	0.38	0.00
OXR1	0.75	0.03	0.59	0.00	1.18	0.05	1.52	0.02
PADI2	1.51	0.01	1.41	0.05	1.08	0.05	0.44	0.00
PCDH7	1.33	0.05	1.53	0.02	0.84	0.03	1.66	0.03
PCGF2	1.57	0.01	1.47	0.01	0.92	0.02	1.66	0.05
PCMT1	0.41	0.00	0.87	0.04	0.91	0.04	1.37	0.04
PDGFRA	1.26	0.01	1.27	0.03	1.74	0.00	2.28	0.06
PDLIM4	1.20	0.02	1.99	0.00	1.24	0.00	0.39	0.01
PFKFB2	0.73	0.05	0.58	0.01	0.56	0.00	2.89	0.00
PFKFB4	1.25	0.05	0.80	0.03	0.88	0.01	2.58	0.00
PHACTR2	0.39	0.01	0.81	0.03	0.87	0.05	1.63	0.03
PPP1R13L	1.74	0.00	0.41	0.00	0.55	0.00	0.65	0.03
PSIP1	0.48	0.02	0.77	0.01	0.85	0.01	1.62	0.04
PTPRC	1.35	0.01	1.15	0.04	1.43	0.00	2.50	0.03
RAB2A	0.25	0.00	0.81	0.06	0.89	0.02	1.34	0.05
RHOBTB3	0.30	0.00	0.61	0.00	0.77	0.00	1.46	0.02
SCAMP1	0.41	0.00	0.82	0.04	0.85	0.02	1.68	0.02
SDS	1.25	0.03	1.29	0.04	1.29	0.01	0.45	0.02
SEC24D	0.34	0.00	0.83	0.04	0.85	0.01	1.77	0.01
SERPINB1	0.59	0.02	1.42	0.02	1.27	0.02	1.71	0.01
SGSM2	1.57	0.01	2.66	0.00	0.88	0.03	0.42	0.04
SLC1A2	1.37	0.01	1.33	0.04	1.31	0.02	0.52	0.04
SLC25A36	0.44	0.02	1.35	0.00	0.86	0.02	2.04	0.01
SLC7A11	0.52	0.00	2.02	0.05	1.34	0.01	3.11	0.00
SNX13	1.45	0.00	0.43	0.02	0.89	0.01	1.39	0.04
STK17A	0.52	0.05	1.54	0.01	1.25	0.02	1.68	0.01
SYT13	1.54	0.01	1.30	0.04	0.66	0.00	1.39	0.04
TACSTD2	1.34	0.03	1.44	0.01	1.27	0.04	1.56	0.03
TLK1	0.29	0.00	1.95	0.03	0.87	0.00	2.38	0.04
TRAK1	0.59	0.01	0.81	0.04	0.83	0.02	2.47	0.02
TSN	0.34	0.00	0.74	0.05	0.91	0.01	1.50	0.05
TTLL4	1.30	0.00	0.66	0.02	1.15	0.01	2.47	0.01
UNC93B1	1.56	0.00	1.89	0.00	1.15	0.03	0.53	0.05
VPS33A	1.17	0.05	1.95	0.03	0.89	0.02	1.86	0.06
WISP1	1.33	0.04	2.62	0.01	1.18	0.01	0.32	0.01
ZFAND6	0.49	0.01	0.86	0.02	0.90	0.04	1.37	0.06
ZNF148	0.54	0.00	0.85	0.04	0.92	0.04	1.78	0.00
ZNF358	1.90	0.00	1.29	0.01	0.90	0.01	0.41	0.02

Table S9: Opposite regulated differentially expressed genes of diabetic pancreatic tissue and diabetic liver tissue (Group: 3).

	**Pancreas GSE25724**		**Pancreas GSE20966**		**Pancreas GSE38642**		**Liver GSE23343**	
Gene Symbol	**FCE**	**p-Value**	**FCE**	**p-Value**	**FCE**	**p-Value**	**FCE**	**p-Value**
WISP1	1.33	0.04	2.62	0.01	1.18	0.01	0.32	0.01
CDK5RAP2	1.35	0.01	1.55	0.02	0.90	0.04	0.35	0.00
NRG1	1.41	0.03	1.68	0.06	1.09	0.01	0.38	0.00
PDLIM4	1.20	0.02	1.99	0.00	1.24	0.00	0.39	0.01
PADI2	1.51	0.01	1.41	0.05	1.08	0.05	0.44	0.00
SDS	1.25	0.03	1.29	0.04	1.29	0.01	0.45	0.02
SLC1A2	1.37	0.01	1.33	0.04	1.31	0.02	0.52	0.04
UNC93B1	1.56	0.00	1.89	0.00	1.15	0.03	0.53	0.05
EEF1D	1.41	0.00	1.31	0.00	1.13	0.02	0.54	0.05
HSD11B2	1.49	0.00	1.73	0.04	1.15	0.04	0.73	0.03
RAB2A	0.25	0.00	0.81	0.06	0.89	0.02	1.34	0.05
PCMT1	0.41	0.00	0.87	0.04	0.91	0.04	1.37	0.04
ZFAND6	0.49	0.01	0.86	0.02	0.90	0.04	1.37	0.06
BTBD1	0.40	0.01	0.80	0.03	0.92	0.03	1.38	0.03
NBEA	0.33	0.01	0.78	0.00	0.82	0.04	1.44	0.06
RHOBTB3	0.30	0.00	0.61	0.00	0.77	0.00	1.46	0.02
ARL1	0.38	0.00	0.80	0.01	0.88	0.01	1.48	0.04
TSN	0.34	0.00	0.74	0.05	0.91	0.01	1.50	0.05
PSIP1	0.48	0.02	0.77	0.01	0.85	0.01	1.62	0.04
PHACTR2	0.39	0.01	0.81	0.03	0.87	0.05	1.63	0.03
SCAMP1	0.41	0.00	0.82	0.04	0.85	0.02	1.68	0.02
APC	0.36	0.00	0.71	0.02	0.82	0.01	1.72	0.00
SEC24D	0.34	0.00	0.83	0.04	0.85	0.01	1.77	0.01
ZNF148	0.54	0.00	0.85	0.04	0.92	0.04	1.78	0.00
TRAK1	0.59	0.01	0.81	0.04	0.83	0.02	2.47	0.02
ARHGEF9	0.50	0.02	0.64	0.00	0.74	0.01	2.71	0.02
PFKFB2	0.73	0.05	0.58	0.01	0.56	0.00	2.89	0.00

Table S10: Down regulated differentially expressed genes of diabetic pancreatic tissue and diabetic liver tissue (Group: 3).

	**Pancreas GSE25724**		**Pancreas GSE20966**		**Pancreas GSE38642**		**Liver GSE23343**	
Gene Symbol	FCE	p-Value	FCE	p-Value	FCE	p-Value	FCE	p-Value
ATP2B2	1.63	0.00	2.03	0.03	1.14	0.00	1.84	0.06
FRMD4B	1.33	0.01	1.43	0.02	1.17	0.04	1.64	0.01
HGF	1.46	0.01	3.38	0.00	1.44	0.01	1.77	0.01
MCL1	1.46	0.01	1.37	0.05	1.18	0.01	1.42	0.03
NR6A1	1.42	0.00	1.61	0.01	1.1	0.05	2.55	0.00
PDGFRA	1.26	0.01	1.27	0.03	1.74	0.00	2.28	0.06
PTPRC	1.35	0.01	1.15	0.04	1.43	0.00	2.5	0.03
TACSTD2	1.34	0.03	1.44	0.01	1.27	0.04	1.56	0.03

Table S11: Common Down regulated differentially expressed genes of diabetic pancreatic tissue and diabetic liver tissue (Group: 3).

	Pancreas (GSE25724)		Pancreas (GSE20966)		Pancreas (GSE38642)		Liver (GSE23343)	
Gene Symbol	FCE	p-Value	FCE	p-Value	FCE	p-Value	FCE	p-Value
GTF3C3	0.4	0.00	0.55	0.01	0.91	0.02	0.35	0.03

Table S12: Gene-ontology (Biological Process) analysis of Group: 1 using DAVID Bioinformatics Resources 6.8.

Biological Process (Group:1)

**Term**	**Count**	**PValue**	**Negative -log10 (P Value)**	**Genes**	**Fold Enrichment**	**Bonferroni**	**Benjamini**	**FDR**
Semaphorin-plexin signaling pathway	5	0.001	2.92	SEMA5A, MET, SEMA3D, SEMA3C, SEMA3A	10.335	0.81	0.81	1.97
Negative regulation of axon extension involved in axon guidance	5	0.002	2.77	SEMA5A, RYK, SEMA3D, SEMA3C, SEMA3A	9.474	0.9	0.68	2.75
Negative regulation of centriole replication	3	0.003	2.55	TRIM37, CDK5RAP2, MDM1	34.105	0.98	0.72	4.48
Positive regulation of transcription from RNA polymerase II promoter	24	0.005	2.34	PID1, IKZF4, THRA, KLF12, CRLF3, RELB, MET, DCN, PDX1, CBFA2T2, SIRT2, ADCYAP1, CITED2, IL11, NFATC2IP, OSR2, ZNF148, DBP, PSIP1, MYOG, PIAS2, NRG1, SLC30A9, NKX2-2	1.882	1	0.78	7.16
Adult walking behavior	5	0.006	2.25	NPC1, AGTPBP1, SPTBN4, HIPK2, KLHL1	6.89	1	0.78	8.82
Positive regulation of DNA binding	4	0.009	2.05	HIPK2, TXN, SIRT2, PLAUR	9.095	1	0.87	13.8
Prostate gland growth	3	0.012	1.91	AR, PSAP, PTEN	17.053	1	0.91	18.35
Positive regulation of gtpase activity	9	0.014	1.87	DVL2, WNT4, CCL21, RANBP1, GNAS, ARHGAP29, ARHGEF9, ADCYAP1, ALS2CL	2.862	1	0.9	19.99
Neural crest cell migration	5	0.015	1.81	SEMA5A, EDN3, SEMA3D, SEMA3C, SEMA3A	5.167	1	0.9	22.52
Leydig cell differentiation	3	0.016	1.81	AR, PDGFRA, NR0B1	15.158	1	0.88	22.69
Activation of MAPK activity	5	0.017	1.78	LPAR3, HGF, NRG1, FRS2, MAP2K6	5.053	1	0.87	24.08
Cardiac muscle contraction	4	0.021	1.68	SRSF1, PPP1R13L, MAP2K6, CASQ2	6.737	1	0.9	29.01
Negative regulation of cell proliferation	11	0.022	1.66	ALDH1A2, ING4, TNS2, KAT2B, EIF2AK1, PTGES,	2.305	1	0.9	30.14

				MYOG, PDX1, HMGA1, SIRT2, APC				
Barbed-end actin filament capping	3	0.023	1.64	GSN, CAPZA1, ADD2	12.402	1	0.89	31.85
Retrograde transport, endosome to Golgi	5	0.024	1.63	ARL1, TBC1D5, SNX2, CLN5, RHOBTB3	4.547	1	0.88	32.48
Transforming growth factor beta receptor signaling pathway	5	0.027	1.57	SMAD9, HIPK2, TGFBR3, SMURF1, CITED2	4.372	1	0.9	36.05
Substantia nigra development	4	0.027	1.56	INA, ZNF148, PADI2, SIRT2	6.063	1	0.89	36.55
Platelet aggregation	4	0.03	1.53	TYRO3, PLEK, PDGFRA, SLC7A11	5.868	1	0.9	39.12
Small gtpase mediated signal transduction	10	0.032	1.5	RAB2A, ARL1, SH2D3A, RAB30, RAC2, ARHGAP35, DOCK5, ARL4D, DOCK10, RHOBTB3	2.285	1	0.9	41

Table S13: Gene-ontology (Molecular Function) analysis of Group: 1 using DAVID Bioinformatics Resources 6.8.

Term	PValue	Genes	Fold Enrichment	Bonferroni	Benjamini	FDR
Chemorepellent activity	0.00	SEMA5A, SEMA3D, SEMA3C, SEMA3A, NRG1	9.51	0.44	0.44	2.26
Enzyme activator activity	0.01	ARL1, GM2A, PSAP, PRKRA	10.14	0.9	0.68	8.63
Semaphorin receptor binding	0.01	SEMA5A, SEMA3D, SEMA3C, SEMA3A	10.14	0.9	0.68	8.63
Neuropilin binding	0.02	SEMA3D, SEMA3C, SEMA3A	15.21	1	0.83	19.15
Gtpase activator activity	0.02	SGSM2, GNAQ, TBC1D5, RANBP1, ARHGAP29, RAP1GAP2, CDC42EP3, ARHGAP26, ALS2CL	2.49	1	0.81	23.22
GTP binding	0.04	RAB2A, ARL1, DNM3, ARHGAP35, RRAGD, RAB30, RAC2, GNAQ, GSPT1, GNAS, ARL4D, EHD1, RHOBTB3, EHD4	1.85	1	0.94	43.14
Heme binding	0.05	CYP2U1, CYB5R4, EIF2AK1, PGRMC1, DUOX1, CYP4F2	2.66	1	0.94	48.74
Protein serine/threonine phosphatase activity	0.05	PPP1CA, CDC14B, PTEN, PPP2R2A	4.8	1	0.92	50.05
RNA polymerase II regulatory region sequence-specific DNA binding	0.06	HLF, KAT2B, OSR2, KLF12, DBP, RELB, ARHGAP35, ETV5	2.32	1	0.92	54.93

Table S14: Gene-ontology (Cellular Components) analysis of Group: 1 using DAVID Bioinformatics Resources 6.8.

Term	Count	PValue	Negative log10 (P Value)	Genes	Fold Enrichment	Bonferroni	Benjamini	FDR
cytoplasm	89	0.00	3.05	SRSF1, CDC14B, DYNC2LI1, NCS1, AQP4, PTEN, LZTFL1, KLHL1, IL11, RCBTB1, SH2D1A, WNT4, RANBP9, WISP1, GSN, PTGES, PPP1R1A, SLC2A2, KLHL22, RANBP1, NRG1, CIB2, FRS2, MAP2K6, ALS2CL, PID1, ARL1, AR, STMN3, RELB, PADI2, PRKCH, ARHGAP29, ARHGEF9, NR0B1, CDKL5, PPP1CA, SDC1, EIF2AK1, SGSM2, SDS, FRMD4B, EPB41L5, HIPK2, TXN, PDGFRA, SERPINB1, FLII, PEBP1, ARL4D, ASB4, CRLF3, ARHGAP35, DCN, LMAN1, ALDH1A2, LPXN, PEX19, VRK3, DGKE, MAP3K2, POU2F3, CENPC, PRKRA, PCMT1, RAP1GAP2, CDC42EP3, APC, SMAD9, KLF12, PTPN3, PLEK, RYK, PSAP, TSN, DOCK5, RCAN2, PPP1R13L, CENPI, TRADD, GART, SH3BGRL, RNF6, SYDE1, SMURF1, DHX40, WDR1, HSPD1	1.37	0.21	0.21	1.17
Lysosome	10	0.00	2.56	GNS, DNASE2, SLC17A5, TMEM59, PSAP, KIAA1324, RRAGD, LMBRD1, CLN5, ASAH1	3.41	0.51	0.3	3.52
Axon	8	0.01	2.03	NRCAM, GABRG2, RNF6, STMN3, NCS1, LPAR3, SEMA3A, LRFN3	3.4	0.91	0.55	11.43
Lysosomal membrane	10	0.01	1.88	RAB2A, ATP6V1A, SLC17A5, SLC29A3, NPC1, LAPTM5, GNAQ, TMEM59, LMBRD1, CLN5	2.67	0.97	0.58	15.82
External side of plasma membrane	9	0.02	1.77	CD48, SDC1, IL12RB1, PDPN, CD2, PDGFRA, AQP4, TGFBR3, NRG1	2.75	0.99	0.59	19.93
Cell body	4	0.04	1.36	SYNDIG1, GNAQ, GNG3, NRG1	5.05	1	0.86	44.36
Cytoskeleton	8	0.05	1.3	PTPN3, GSN, FRMD4B, EPB41L5, SLC30A9, MAP2K6, ADD2, TRADD	2.4	1	0.85	48.6
Podosome	3	0.06	1.23	LPXN, WDR1, TPM4	7.58	1	0.86	54.39
Nuclear membrane	7	0.06	1.23	INA, RNF6, OSBPL8, CDC14B, HN1, RAP1GAP2, GTF3C3	2.53	1	0.83	54.85

Table S15: Pathway Analysis (KEGG Pathway) analysis of Group: 1 using DAVID Bioinformatics Resources 6.8.

**Group :1 KEGG Pathway**							
**Term**	**PValue**	**Negative log10 (Pvalue)**	**Genes**	**Fold Enrichment**	**Bonferroni**	**Benjamini**	**FDR**
Maturity onset diabetes of the young	0.00	2.69	IAPP, SLC2A2, PDX1, HNF4G, NKX2-2	8.96	0.33	0.33	2.55
Malaria	0.01	2.28	GYPC, SDC1, MET, HGF, THBS2, SDC2	5.28	0.64	0.40	6.32
Lysosome	0.01	2.27	GNS, DNASE2, SLC17A5, NPC1, LAPTM5, GM2A, PSAP, CLN5, ASAH1	3.33	0.65	0.30	6.52
Insulin secretion	0.01	2.1	GNAQ, SLC2A2, GNAS, PDX1, ABCC8, CACNA1D, ADCYAP1	3.98	0.79	0.32	9.44
Adrenergic signaling in cardiomyocytes	0.01	2.07	ATP2B2, PPP1CA, CACNA2D1, GNAQ, PPP1R1A, GNAS, CACNA1D, TPM4, PPP2R2A	3.08	0.81	0.28	10.04
Cell adhesion molecules (CAMs)	0.02	1.77	NRCAM, PTPRC, MPZL1, SDC1, CADM1, CD2, MADCAM1, NRXN1, SDC2	2.72	0.97	0.43	19.26
Butanoate metabolism	0.02	1.68	ABAT, AACS, HADH, BDH1	6.66	0.99	0.45	23.40
Proteoglycans in cancer	0.03	1.53	SDC1, WNT4, PPP1CA, MET, PTCH1, DCN, HGF, FRS2, SDC2, PLAUR	2.30	1.00	0.52	31.07
GnRH signaling pathway	0.04	1.44	GNAQ, MAP3K2, GNAS, GNRHR, CACNA1D, MAP2K6	3.25	1.00	0.55	36.97
Pathways in cancer	0.04	1.35	DVL2, DCC, AR, MET, LPAR3, HGF, PTEN, WNT4, RAC2, GNAQ, PDGFRA, PTCH1, GNAS, GNG3, APC	1.76	1.00	0.59	43.30
Axon guidance	0.05	1.3	SEMA5A, DCC, RAC2, MET, SEMA3D, SEMA3C, SEMA3A	2.61	1.00	0.61	47.72
Type II diabetes mellitus	0.08	1.11	SLC2A2, PDX1, ABCC8, CACNA1D	3.97	1.00	0.74	63.84
Glutamatergic synapse	0.09	1.03	TRPC1, SLC1A2, GNAQ, GNAS, GNG3, CACNA1D	2.47	1.00	0.77	70.65

Table S16: Gene-ontology (Biological Process) analysis of Group: 2 using DAVID Bioinformatics Resources 6.8.

Group 2 Biological Process								
**Term**	**Count**	**P Value**	**Negative log10 p value**	**Genes**	**Fold Enrichment**	**Bonferroni**	**Benjamini**	**FDR**
Positive regulation of catalytic activity	11	0.00	3.11	PTPRC, GNAQ, DGKE, GM2A, GSPT1, MAP3K2, PFKFB2, GNAS, HGF, NRG1, FRS2	3.62	0.60	0.60	1.25
Regulation of kinase activity	9	0.00	2.99	PTPRC, DGKE, MAP3K2, PFKFB2, HGF, NRG1, FRS2, PTEN, APC	4.32	0.70	0.45	1.64
Regulation of transferase activity	9	0.00	2.88	PTPRC, DGKE, MAP3K2, PFKFB2, HGF, NRG1, FRS2, PTEN, APC	4.14	0.79	0.41	2.12
Positive regulation of molecular function	11	0.00	2.72	PTPRC, GNAQ, DGKE, GM2A, GSPT1, MAP3K2, PFKFB2, GNAS, HGF, NRG1, FRS2	3.21	0.90	0.43	3.03
Positive regulation of kinase activity	7	0.00	2.67	PTPRC, DGKE, MAP3K2, PFKFB2, HGF, NRG1, FRS2	5.19	0.92	0.40	3.40
Positive regulation of transferase activity	7	0.00	2.59	PTPRC, DGKE, MAP3K2, PFKFB2, HGF, NRG1, FRS2	4.99	0.95	0.40	4.11
Regulation of protein kinase activity	8	0.00	2.43	PTPRC, DGKE, MAP3K2, HGF, NRG1, FRS2, PTEN, APC	3.97	0.99	0.47	5.81
Regulation of phosphorylation	9	0.01	2.28	PTPRC, DGKE, MAP3K2, PFKFB2, HGF, NRG1, FRS2, PTEN, APC	3.31	1.00	0.55	8.23
Vesicle-mediated transport	10	0.01	2.24	SCAMP1, RAB2A, ARL1, NPC1, SYT13, SEC24D, VPS33A, ERGIC3, RHOBTB3, EHD4	2.97	1.00	0.53	8.90
Vegulation of phosphorus metabolic process	9	0.01	2.17	PTPRC, DGKE, MAP3K2, PFKFB2, HGF, NRG1, FRS2, PTEN, APC	3.18	1.00	0.55	10.30
Regulation of phosphate metabolic process	9	0.01	2.17	PTPRC, DGKE, MAP3K2, PFKFB2, HGF, NRG1, FRS2, PTEN, APC	3.18	1.00	0.55	10.30
Positive regulation of protein kinase activity	6	0.01	2.03	PTPRC, DGKE, MAP3K2, HGF, NRG1, FRS2	4.61	1.00	0.63	13.97
Activation of MAPK activity	4	0.01	1.93	PTPRC, MAP3K2, HGF, FRS2	8.35	1.00	0.69	17.42
Positive regulation of MAP kinase activity	4	0.02	1.68	PTPRC, MAP3K2, HGF, FRS2	6.72	1.00	0.86	29.08
Fructose 2,6-bisphosphate metabolic process	2	0.02	1.64	PFKFB4, PFKFB2	85.62	1.00	0.86	31.17

Table S17: Gene-ontology (Cellular Components) analysis of Group: 2 using DAVID Bioinformatics Resources 6.8.

**Group:2 Cellular Function**								
**Term**	**Count**	**PValue**	**Negative Log P value**	**Genes**	**Fold Enrichment**	**Bonferroni**	**Benjamini**	**FDR**
Trans-Golgi network	4	0.002	2.77	SCAMP1, ARL1, GNAS, NBEA	16.59	0.267	0.267	2.08
Golgi apparatus	11	0.013	1.87	SCAMP1, RAB2A, ARL1, MPHOSPH9, ZNF148, GNAS, NBEA, FGD6, SEC24D, ERGIC3, RHOBTB3	2.41	0.915	0.708	15.36
Plasma membrane part	20	0.014	1.87	ARL1, GYPC, PTPRC, GM2A, PCDH7, PIGR, VPS33A, ATP2B2, NPC1, SLC1A2, TACSTD2, EPB41L5, PDGFRA, SYT13, GNAS, MAP7, NRG1, ENTPD1, FRS2, APC	1.73	0.917	0.564	15.53
Lytic vacuole	5	0.023	1.63	NPC1, GM2A, LMBRD1, VPS33A, ASAH1	4.52	0.987	0.661	25.41
Lysosome	5	0.023	1.63	NPC1, GM2A, LMBRD1, VPS33A, ASAH1	4.52	0.987	0.661	25.41
Vacuole	5	0.041	1.39	NPC1, GM2A, LMBRD1, VPS33A, ASAH1	3.79	1.000	0.783	40.42
Transport vesicle	3	0.046	1.34	SYT13, PIGR, SEC24D	8.67	1.000	0.757	43.77
Golgi apparatus part	5	0.065	1.19	SCAMP1, ARL1, GNAS, NBEA, SEC24D	3.24	1.000	0.827	56.50
Cytosol	12	0.077	1.11	PFKFB4, MAP3K2, TACSTD2, PFKFB2, PSIP1, NBEA, CDK5RAP2, ARHGEF9, EEF1D, PTEN, CRYZL1, APC	1.72	1.000	0.840	62.97
Endosome	5	0.080	1.10	SCAMP1, NPC1, FRS2, VPS33A, EHD4	3.03	1.000	0.813	64.06
Integral to plasma membrane	11	0.083	1.08	GYPC, PTPRC, NPC1, GM2A, TACSTD2, PDGFRA, SYT13, PCDH7, PIGR, ENTPD1, FRS2	1.77	1.000	0.792	65.51
Intrinsic to plasma membrane	11	0.093	1.03	GYPC, PTPRC, NPC1, GM2A, TACSTD2, PDGFRA, SYT13, PCDH7, PIGR, ENTPD1, FRS2	1.73	1.000	0.800	69.93

Table S18: Gene-ontology (Molecular Function) analysis of Group: 2 using DAVID Bioinformatics Resources 6.8.

**Group:2 Cellular Function**								
**Term**	**Count**	**PValue**	**Negative Log P value**	**Genes**	**Fold Enrichment**	**Bonferroni**	**Benjamini**	**FDR**
Trans-Golgi network	4	0.00	2.77	SCAMP1, ARL1, GNAS, NBEA	16.59	0.27	0.27	2.08
Golgi apparatus	11	0.01	1.87	SCAMP1, RAB2A, ARL1, MPHOSPH9, ZNF148, GNAS, NBEA, FGD6, SEC24D, ERGIC3, RHOBTB3	2.41	0.92	0.71	15.36
Plasma membrane part	20	0.01	1.87	ARL1, GYPC, PTPRC, GM2A, PCDH7, PIGR, VPS33A, ATP2B2, NPC1, SLC1A2, TACSTD2, EPB41L5, PDGFRA, SYT13, GNAS, MAP7, NRG1, ENTPD1, FRS2, APC	1.73	0.92	0.56	15.53
Lytic vacuole	5	0.02	1.63	NPC1, GM2A, LMBRD1, VPS33A, ASAH1	4.52	0.99	0.66	25.41
Lysosome	5	0.02	1.63	NPC1, GM2A, LMBRD1, VPS33A, ASAH1	4.52	0.99	0.66	25.41
Vacuole	5	0.04	1.39	NPC1, GM2A, LMBRD1, VPS33A, ASAH1	3.79	1.00	0.78	40.42
Transport vesicle	3	0.05	1.34	SYT13, PIGR, SEC24D	8.67	1.00	0.76	43.77
Golgi apparatus part	5	0.07	1.19	SCAMP1, ARL1, GNAS, NBEA, SEC24D	3.24	1.00	0.83	56.50
Cytosol	12	0.08	1.11	PFKFB4, MAP3K2, TACSTD2, PFKFB2, PSIP1, NBEA, CDK5RAP2, ARHGEF9, EEF1D, PTEN, CRYZL1, APC	1.72	1.00	0.84	62.97
Endosome	5	0.08	1.1	SCAMP1, NPC1, FRS2, VPS33A, EHD4	3.03	1.00	0.81	64.06
Integral to plasma membrane	11	0.08	1.08	GYPC, PTPRC, NPC1, GM2A, TACSTD2, PDGFRA, SYT13, PCDH7, PIGR, ENTPD1, FRS2	1.77	1.00	0.79	65.51
Intrinsic to plasma membrane	11	0.09	1.03	GYPC, PTPRC, NPC1, GM2A, TACSTD2, PDGFRA, SYT13, PCDH7, PIGR, ENTPD1, FRS2	1.73	1.00	0.80	69.93

Table S19: Pathway Analysis (KEGG Pathway) analysis of Group: 2 using DAVID Bioinformatics Resources 6.8.

**Group:2 KEGG Pathway**								
**Term**	**Count**	**PValue**	**Negative Log P Value**	**Genes**	**Fold Enrichment**	**Bonferroni**	**Benjamini**	**FDR**
Gap junction	4	0.011	1.95	GNAQ, MAP3K2, PDGFRA, GNAS	8.16	0.462	0.462	10.48
Melanoma	3	0.054	1.27	PDGFRA, HGF, PTEN	7.67	0.953	0.782	42.02
Calcium signaling pathway	4	0.065	1.19	ATP2B2, GNAQ, PDGFRA, GNAS	4.13	0.975	0.708	48.32
GnRH signaling pathway	3	0.095	1.02	GNAQ, MAP3K2, GNAS	5.56	0.996	0.745	62.36

Table S20: Gene-ontology (Biological Process) analysis of Group: 3 using DAVID Bioinformatics Resources 6.8.

**Group: 3 Biological Process**							
**Term**	**PValue**	**Negative P value**	**Genes**	**Fold Enrichment**	**Bonferroni**	**Benjamini**	**FDR**
Negative regulation of axon extension involved in axon guidance	0.00	3.55	SEMA5A, SEMA3D, SEMA3C, SEMA3A	30.75	0.18	0.18	0.42
Semaphorin-plexin signaling pathway	0.00	3.24	SEMA5A, SEMA3D, SEMA3C, SEMA3A	24.23	0.33	0.18	0.86
Negative chemotaxis	0.00	3.20	SEMA5A, SEMA3D, SEMA3C, SEMA3A	23.52	0.35	0.13	0.93
Positive regulation of actin filament depolymerization	0.00	3.18	SEMA5A, PLEK, WDR1	74.96	0.37	0.11	0.99
Neural crest cell migration	0.00	2.85	SEMA5A, SEMA3D, SEMA3C, SEMA3A	17.77	0.63	0.18	2.12
Positive regulation of transforming growth factor beta receptor signaling pathway	0.01	2.21	HIPK2, TGFBR3, CITED2	24.99	0.99	0.51	8.95
Adult walking behavior	0.01	1.99	NPC1, HIPK2, OXR1	19.35	1.00	0.64	14.34
Transforming growth factor beta receptor signaling pathway	0.01	1.97	SMAD9, HIPK2, TGFBR3, CITED2	8.69	1.00	0.60	14.90
Outflow tract morphogenesis	0.02	1.66	TGFBR3, SEMA3C, CITED2	13.04	1.00	0.81	28.13
Nuclear transport	0.02	1.61	PSIP1, HMGA1	79.96	1.00	0.82	31.13
Dichotomous subdivision of terminal units involved in salivary gland branching	0.02	1.61	SEMA3C, SEMA3A	79.96	1.00	0.82	31.13
Establishment of integrated proviral latency	0.04	1.41	PSIP1, HMGA1	49.98	1.00	0.92	44.94
Positive regulation of male gonad development	0.04	1.41	SEMA3A, CITED2	49.98	1.00	0.92	44.94
Post-embryonic development	0.05	1.30	GNAQ, SEMA3C, PPP1R13L	8.22	1.00	0.95	54.24
Embryonic camera-type eye morphogenesis	0.06	1.24	HIPK2, CITED2	33.32	1.00	0.96	59.15

Table S21: Gene-ontology (Molecular Function) analysis of Group: 3 using DAVID Bioinformatics Resources 6.8.

**Group 3: Molecular Function**								
**Term**	**Count**	**P value**	**Negative log10 (p value)**	**Genes**	**Fold Enrichment**	**Bonferroni**	**Benjamini**	**FDR**
Chemorepellent activity	5	0.00	5.06	SEMA5A, SEMA3D, SEMA3C, SEMA3A, NRG1	37.22	0.00	0.00	0.01
Semaphorin receptor binding	4	0.00	3.72	SEMA5A, SEMA3D, SEMA3C, SEMA3A	34.95	0.04	0.02	0.24
Neuropilin binding	3	0.00	2.62	SEMA3D, SEMA3C, SEMA3A	40.19	0.39	0.15	2.99
RNA polymerase II transcription coactivator activity	3	0.01	1.85	HIPK2, PSIP1, CITED2	16.29	0.95	0.52	16.50
Transcription corepressor activity	5	0.02	1.75	RELB, HIPK2, CBFA2T2, PPP1R13L, CITED2	4.95	0.98	0.52	20.27
Amino acid transmembrane transporter activity	3	0.02	1.65	SLC1A2, PDPN, SLC7A6	12.83	0.99	0.54	24.78
Water transmembrane transporter activity	2	0.03	1.54	PDPN, AQP4	66.99	1.00	0.58	31.08
Transforming growth factor beta-activated receptor activity	2	0.03	1.47	LTBP4, TGFBR3	57.42	1.00	0.59	35.23

Table S22: Gene-ontology (Cellular Components) analysis of Group: 3 using DAVID Bioinformatics Resources 6.8.

Group 3: Cellular Component

**Term**	**PValue**	**Negative log10 Pvalue**	**Genes**	**Fold Enrichment**	**Bonferroni**	**Benjamini**	**FDR**
Microtubule organizing center	0.005	2.28	TAF1D, RELB, FLII, CDK5RAP2, AKAP11	7.09	0.55	0.546	6.09
Ruffle	0.008	2.09	PDPN, FRMD4B, SAMSN1, FGD6	9.64	0.70	0.455	9.20
External side of plasma membrane	0.016	1.79	ITGAX, PDPN, AQP4, TGFBR3, NRG1	5.09	0.91	0.560	17.78
Cytoplasm	0.019	1.72	NCS1, AQP4, CITED2, DGKE, CENPC, TRAK1, PRKRA, SNTB1, RANBP1, KLHL24, CDK5RAP2, HADH, NRG1, FGD6, IQSEC1, SMAD9, PLEK, RELB, ACLY, ARHGEF9, DOCK5, PPP1R13L, GART, PLEKHA4, PPP1CA, RAB30, GNAQ, POLDIP3, FRMD4B, HIPK2, FLII, TGFBR3, WDR1, EEF1D	1.41	0.94	0.515	20.57
Membrane	0.019	1.71	PLEK, AQP4, ACLY, MAGED2, SEMA5A, CDS2, PLEKHA4, SLC1A2, NPC1, ITGAX, DGKE, GNAQ, PRKRA, ERAP1, ENTPD1, NRG1, IQSEC1, PIGA	1.78	0.95	0.445	20.87
Primary cilium	0.049	1.31	DYNC2LI1, RABGAP1L, PTCH1	8.34	1.00	0.717	45.29
Integral component of plasma membrane	0.056	1.25	TRPC1, MUSK, NPC1, LAPTM5, PDPN, AQP4, TGFBR3, PCDH7, ENTPD1, NRG1, SLC7A6, PLAUR	1.84	1.00	0.707	49.55
Cytosol	0.066	1.18	PDCD11, SMAD9, PLEK, PFKFB2, DYNC2LI1, RELB, NCS1, ACLY, ARHGEF9, SAMSN1, HMGA1, GART, PPP1CA, POLDIP3, CENPC, PRKRA, PSIP1, TGM2, ERAP1, WDR1, CDK5RAP2, EEF1D	1.44	1.00	0.721	55.70
Axolemma	0.070	1.15	SLC1A2, NRG1	27.12	1.00	0.704	58.21

Table S23: Pathway Analysis (KEGG Pathway) analysis of Group: 3 using DAVID Bioinformatics Resources 6.8.

**Group 3: KEGG Pathway**								
**Term**	**Count**	**P-Value**	**negative log p value**	**Genes**	**Fold Enrichment**	**Bonferroni**	**Benjamini**	**FDR**
hsa04360:Axon guidance	4	0.030256899	1.52	SEMA5A, SEMA3D, SEMA3C, SEMA3A	5.70	0.944	0.944	28.61

Table S24. Common regulated differentially expressed genes of diabetic pancreatic tissue, diabetic heart tissue and diabetic liver tissue.

Gene Symbol	Pancreas (GSE25724)	Pancreas (GSE20966)	Pancreas (GSE38642)	Heart (GSE26887)	Liver (GSE23343)
ARHGEF9	0.5	0.64	0.74	1.46	2.71
CBFA2T2	1.43	1.79	0.87	1.21	0.62
CDK5RAP2	1.35	1.16	1.11	1.31	0.35
EEF1D	1.41	1.31	1.13	0.84	0.54
FGD6	0.74	0.83	1.19	1.41	0.28
FRMD4B	1.33	1.43	1.17	1.67	1.64
GNAQ	1.52	0.77	0.91	1.52	1.58
MAML3	1.24	0.66	0.82	1.49	1.94
NPC1	0.53	0.76	1.12	0.65	0.49
NRG1	1.41	1.68	1.09	2.45	0.38
OXR1	0.75	0.59	1.18	1.38	1.52
PCDH7	1.33	1.53	0.84	0.38	1.66
PFKFB2	0.73	0.58	0.56	1.89	2.89
PSIP1	0.48	0.77	0.85	1.28	1.62
SLC1A2	1.37	1.33	1.31	0.79	0.52
TRAK1	0.59	0.81	0.83	1.38	2.47

Table S25: PPI network and identification in all groups by MCODE.

**Network Scoring:**	Degree Cut-off				2
	Include Loops				False
**Cluster Finding:**	Node Score Cut-off				0.2
	Haircut				TRUE
	Fluff				False
	K-Core				2 Max
	Depth from Seed				100
**Type of Groups**	**Cluster**	**Score (Density Nodes)**	**Nodes**	**Edges**	**Node IDs**
Group-1	1	9.5	10	43	POLR1E, DDX10, URB1, HEATR1, DDX18, PDCD11, RSL1D1, RRP12, MAK16, RRP15
	2	8	8	28	TRIM37, BTBD1, RNF19B, ASB4, KLHL22, SMURF1, FBXO7, RNF6
	3	4	14	26	CD2, CD48, EDN3, GNAS, ITGAX, PDPN, GNRHR, RAC2, MADCAM1, WASL, GNAQ, PLEK, LAPTM5, DVL2
Group- 2	1	3.3	4	5	RRP12, PDCD11, RRP15, MRPL3
	2	3	3	3	PLEK, GNAQ, IQSEC1
Group-3	1	3	3	3	BTBD1, ASB4, KLHL22
	2	3	3	3	DDX18, MAK16, RRP15
16 common genes involved in all groups	1	10	10	45	MAML3, NOTCH4, MAML2, HEY2, NOTCH1, HEY1, MAML1, NOTCH2, NOTCH3, RBPJ
	2	6.6	7	20	EEF1G, EEF1A1, EEF1D, EEF1B2, RPLP0, CARS, RPS2
	3	5.7	8	20	NUMB, CDC42, ERBB3, EGFR, GRB2, PSEN1, NRG1, AKT1
	4	4	4	6	CDK5RAP2, CEP152, PCNT, CENPJ

Table S26: Meta-analysis of top 16 hub genes in GSE38642, GSE25724, GSE20966, GSE26887, and GSE23343.

ID	p-Value	FDR -Value	Fold Change mean	FC_GSE20966	FC_GSE23343	FC_GSE25724	FC_GSE26887	FC_GSE38642
ARHGEF9	0.00	0.00	-0.45	-0.11	-1.90	-0.26	0.10	-0.08
CBFA2T2	0.03	0.27	0.44	0.11	1.94	0.13	0.06	-0.04
CDK5RAP2	0.07	0.38	0.20	0.17	0.66	0.11	0.08	-0.03
EEF1D	0.96	1.00	-0.09	-0.02	-0.65	0.21	0.00	0.02
FGD6	0.11	0.45	-0.11	-0.32	-0.25	-0.13	0.10	0.05
FRMD4B	0.16	0.50	0.13	-0.06	0.37	0.18	0.13	0.05
GNAQ	0.02	0.21	-0.16	-0.12	-0.69	-0.12	0.13	-0.02
MAML3	0.94	0.99	-0.12	0.00	-0.76	0.10	0.13	-0.06
NPC1	0.32	0.67	-0.14	-0.01	-0.42	-0.18	-0.12	0.03
NRG1	0.41	0.73	-0.06	0.07	-0.86	0.14	0.34	0.04
OXR1	0.01	0.17	-0.04	-0.90	0.75	-0.17	0.10	0.05
PCDH7	0.87	0.97	-0.03	-0.02	0.15	0.06	-0.30	-0.06
PFKFB2	0.13	0.47	0.03	-0.16	0.42	-0.13	0.18	-0.15
PSIP1	0.18	0.53	-0.20	0.02	-0.77	-0.27	0.09	-0.05
SLC1A2	0.00	0.08	-0.06	0.26	-0.70	0.14	-0.08	0.11
TRAK1	0.32	0.67	-0.09	-0.09	-0.24	-0.14	0.08	-0.05

Table S27. Common regulated MicroRNAs of differentially expressed genes of diabetic pancreatic tissue, diabetic heart tissue, diabetic liver tissue and common DEGs of All three groups.

**Group:1 (Pancreas)**	**Group:2 (Heart)**	**Group:3 (Liver)**	**Common DEGs of All Three Groups (Seed-16 DEGs)**
hsa-let-7b-5p	hsa-mir-16-5p	hsa-mir-1-3p	hsa-mir-16-5p
hsa-mir-107	hsa-mir-124-3p	hsa-mir-155-5p	hsa-mir-27a-3p
hsa-mir-124-3p	hsa-mir-1-3p	hsa-mir-124-3p	hsa-let-7a-5p
hsa-mir-129-2-3p	hsa-mir-27a-3p	hsa-let-7b-5p	hsa-let-7b-5p
hsa-mir-1-3p	hsa-let-7b-5p	hsa-mir-34a-5p	hsa-mir-101-3p
hsa-mir-155-5p	hsa-mir-155-5p	hsa-mir-101-3p	hsa-mir-1-3p
hsa-mir-16-5p	hsa-mir-20a-5p	hsa-mir-15a-5p	hsa-mir-124-3p
hsa-mir-23b-3p	hsa-mir-26b-5p	hsa-mir-26a-5p	hsa-mir-103a-3p
hsa-mir-27a-3p	hsa-mir-27b-3p	hsa-mir-181a-5p	hsa-mir-122-5p
hsa-mir-34a-5p	hsa-mir-147a	hsa-let-7a-5p	hsa-mir-155-5p

Table S28. Top 10 ranked microRNAs selected based on degree and betweenness that regulate the transcriptional expression of common regulated differentially expressed genes of diabetic pancreatic tissue.

Rank	MicroRNA	Degree	Betweenness
1	hsa-mir-1-3p	176	20777.03
2	hsa-mir-16-5p	162	18530.44
3	hsa-mir-124-3p	159	20051.88
4	hsa-mir-155-5p	134	12414.14
5	hsa-mir-34a-5p	118	11163.31
6	hsa-mir-27a-3p	117	16762
7	hsa-let-7b-5p	112	7338.884
8	hsa-mir-129-2-3p	98	9290.168
9	hsa-mir-23b-3p	92	5974.766
10	hsa-mir-107	91	5667.996

Table S29. Top 10 ranked microRNAs selected based on degree and betweenness that regulate the transcriptional expression of common regulated differentially expressed genes of diabetic pancreatic tissue and diabetic liver tissue.

Rank	MicroRNA	Degree	Betweenness
1	hsa-mir-1-3p	49	2581.888
2	hsa-mir-155-5p	39	2110.262
3	hsa-mir-124-3p	38	1898.029
4	hsa-let-7b-5p	36	1376.633
5	hsa-mir-34a-5p	28	1440.665
6	hsa-mir-101-3p	26	758.1382
7	hsa-mir-15a-5p	22	495.6916
8	hsa-mir-26a-5p	22	589.1572
9	hsa-mir-181a-5p	22	516.9619
10	hsa-let-7a-5p	21	336.7557

Table S30. Top 10 ranked microRNAs selected based on degree and betweenness that regulate the transcriptional expression of common regulated differentially expressed genes of diabetic pancreatic tissue and diabetic heart tissue.

Rank	MicroRNA	Degree	Betweenness
1	hsa-mir-16-5p	40	33233.68
2	hsa-mir-124-3p	40	31805.89
3	hsa-mir-1-3p	37	35759.34
4	hsa-mir-27a-3p	34	35602.03
5	hsa-let-7b-5p	29	12711.08
6	hsa-mir-155-5p	27	17536.51
7	hsa-mir-20a-5p	18	6799.166
8	hsa-mir-26b-5p	18	11494.65
9	hsa-mir-27b-3p	18	7877.477
10	hsa-mir-147a	18	8191.25

Table S31. Top 10 ranked microRNAs regulate the transcriptional expression of 16-DEGs for common diabetic pancreatic, diabetic heart and diabetic liver tissues.

Rank	MicroRNA	Degree	Betweenness
1	hsa-mir-16-5p	10	7580.99
2	hsa-mir-27a-3p	9	7682.71
3	hsa-let-7a-5p	8	2811.14
4	hsa-let-7b-5p	8	3967.46
5	hsa-mir-101-3p	7	4824.28
6	hsa-mir-1-3p	7	1945.09
7	hsa-mir-124-3p	7	3717.21
8	hsa-mir-103a-3p	7	2400.84
9	hsa-mir-122-5p	6	1791.97
10	hsa-mir-155-5p	6	1804.80

Table S32. Top 10 ranked microRNAs regulate the transcriptional expression of DEGs expressed genes of diabetic pancreatic tissue.

Rank	MicroRNA	Gene
1	hsa-mir-1-3p	ABCC4, ABHD10, AKAP11, ALDH1A2, APC, ARG2, ARHGAP29, ARL1, ARL4D, ASAH1, ATP6V1A, BTBD1, BTG2, CACNA2D1, CADM1, CAPZA1, CDC42EP3, CDK5RAP2, CDS2, CENPC, CENPI, CITED2, CLGN, CLN5, CPD, CRLF3, CRYZL1, CYB5R4, CYP2U1, CYP4F2, DDX10, DDX18, DHX40, DNASE2, DOCK10, DVL2, DYNC2LI1, EHD1, EIF2AK2, EPB41L5, ERAP1, ETV5, ETV7, F5, FAM222B, FBXO7, FECH, FOXJ3, GLT8D1, GM2A, GNAQ, GNAS, GNRHR, GNS, GOLGA5, GRPEL1, GSN, HADH, HIPK2, HMGN5, IL11, INTS6, KAT2B, KIAA1324, KLHL18, KLHL24, LAPTM5, LMAN1, LMBRD1, LPXN, LZTFL1, MADCAM1, MAGED2, MAK16, MAP3K2, MCL1, MDM1, MET, MIA2, MPHOSPH9, MPZL1, MRPL3, NBEA, NCS1, NENF, NFATC2IP, NIPSNAP1, NPC1, NR6A1, NXPE3, OSBPL8, PADI2, PCDH7, PDCD11, PDGFRA, PDLIM4, PDPN, PEBP1, PEX19, PFKFB2, PGRMC1, PHACTR2, PID1, PIGA, PLAUR, PMAIP1, POLR1E, PPIF, PPP1CA, PPP2R2A, PSIP1, PTPN3, PYY, RAB2A, RAB30, RAC2, RANBP1, RANBP9, RHOBTB3, RNF6, RPS19, RRAGD, RRP12, RSL1D1, SCAMP1, SCD, SDC1, SEC24D, SEMA3A, SEMA3C, SERPINB1, SH3BGRL, SIRT2, SLC17A5, SLC19A1, SLC1A2, SLC25A13, SLC25A36, SLC29A3, SLC30A9, SLC7A11, SMAD9, SMURF1, SNX13, SNX2, SRSF1, STK17A, STMN3, SYDE1, TACSTD2, TAF1D, TBC1D5, TCEA2, TEAD4, TGFBR3, TGM2, THBS2, TLK1, TMEM39A, TMPO, TNFRSF10B, TNFRSF14, TRAPPC13, TRIM37, TRIP10, TSN, TTLL4, TYRO3, UBL3, UFSP2, UNC93B1, VRK3, WASL, YIPF2, ZFAND6, ZNF148
2	hsa-mir-16-5p	ABAT, ABCC4, ABHD10, ACLY, ACVR1B, ADCYAP1, ADD2, AGTPBP1, AKAP11, APC, APPL2, ARG2, ARHGEF9, ASAH1, ATP6V1A, BDH1, BTBD1, BTG2, CACNA2D1, CADM1, CAPZA1, CBFA2T2, CDC14B, CDC42EP3, CDS2, CENPC, CENPI, CITED2, CLCN4, CLGN, CPD, CYP2U1, DBP, DDX10, DDX18, DHRS2, DOCK5, DVL2, EEF1D, EIF2AK2, FECH, FGD6, FLII, FRMD4B, GART, GLT8D1, GM2A, GNAQ, GNS, GOLGA5, GSN, GSPT1, GTF3C3, HADH, HEATR1, HGF, HIPK2, IKZF4, IL11, INA, KLHL1, KLHL22, KLHL24, LAPTM5, LMAN1, LMBRD1, LPAR3, LPXN, LRFN3, MAGED2, MAK16, MAP3K2, MCL1, MET, MREG, MRPL3, MTUS2, NBEA, NEBL, NFATC2IP, NIPSNAP1, NPC1, NR6A1, NXPE3, OSBPL8, PCDH7, PCMT1, PEX19, PFKFB4, PGRMC1, PHACTR2, PIAS2, PIGA, PLAUR, PNMA2, POLR1E, PPIF, PRKCH, PRKRA, PSAP, PSIP1, PTCH1, PTGES, PTPN3, RAB30, RAC2, RANBP9, RCAN2, RELB, RHOBTB3, RPS19, RRAGD, RRP12, RSL1D1, RYK, SCD, SDC1, SEC24D, SEMA3A, SEMA3C, SERPINB1, SGSM2, SH3BGRL, SH3BP2, SLC17A5, SLC19A1, SLC25A13, SLC25A36, SMAD9, SMURF1, SNX2, SNX13, SRD5A1, SRSF1, SYDE1, TACSTD2, TAF1D, TBC1D5, TBC1D19, TCEA2, TGFBR3, TGM2, THRA, TLK1, TMEM59, TMPO, TNFRSF10B, TNFRSF14, TRAK1, TRIM37, TSN, TYRO3, UBL3, UFSP2, VPS33A, VRK3, WDR1, WNT4, WT1, YIPF2, ZFAND6, ZNF148
3	hsa-mir-124-3p	ABCC4, ACLY, ACVR1B, AKAP11, APPL2, ARHGAP26, ARHGAP29, ARHGEF26, ATP6V1A, BTG2, CACNA2D1, CAPZA1, CDC14B, CDC42EP3, CENPC, CENPI, CITED2, CLGN, CLN5, CORO2A, CPD, CRYZL1, CTNNA2, CYB5R4, CYP2U1, CYP4F2, DCC, DHX40, DNASE2, DVL2, DYNC2LI1, EEF1D, EIF2AK2, EPB41L5, F5, FAM222B, FECH, FLII, GABRG2, GART, GLT8D1, GM2A, GNRHR, GNS, GSN, HADH, HEATR1, HIPK2, HLF, HMGN5, HSD11B2, IAPP, IKZF4, IL11, INA, KAT2B, KLHL1, KLHL24, LAPTM5, LMAN1, LRFN3, LZTFL1, MADCAM1, MAK16, MAP2K6, MAP3K2, MCL1, MDM1, MET, MIA2, MPHOSPH9, MPZL1, MREG, MRPL3, MTUS2, NBEA, NEBL, NFATC2IP, NIPSNAP1, NPC1, NRCAM, NRG1, NXPE3, OSBPL8, OXR1, PCDH7, PCMT1, PDCD11, PEBP1, PFKFB2, PHACTR2, PHF7, PID1, PIGA, PLAUR, PLEKHA4, PMAIP1, PPIF, PPP1R13L, PPP1R1A, PPP2R2A, PTGES, PTPN3, PTPRC, RAB2A, RAB30, RAC2, RANBP1, RANBP9, RAP1GAP2, RHOBTB3, RNF6, RRAGD, RRP12, RYK, SCAMP1, SCD, SDC1, SEC24D, SEMA3A, SEMA3C, SH2D3A, SH3BGRL, SH3BP2, SIRPA, SLC17A5, SLC19A1, SLC25A13, SLC25A36, SLC30A9, SLC7A11, SMAD9, SNX13, SRD5A1, SRSF1, STK17A, TAF1D, TBC1D19, TBC1D5, TEAD4, TGFBR3, TGM2, THRA, TMEM39A, TMPO, TNFRSF14, TRAK1, TRAPPC13, TRIM37, TRPC1, TSN, UBL3, VPS33A, VRK3, WDR1, WNT4, ZFAND6, ZNF148, ZNF280B
4	hsa-mir-155-5p	ABAT, ABCC4, ACLY, AKAP11, ALDH1A2, APC, APPL2, ARHGEF9, ARL1, BDH1, BTBD1, BTG2, CDC14B, CDC42EP3, CDKL5, CITED2, CLCN4, CLGN, CLN5, CPD, CRLF3, CYP2U1, DDX10, DDX18, DHX40, DOCK10, DYNC2LI1, EEF1D, EHD1, EIF2AK2, ETV5, F5, FECH, FLII, FOXJ3, GART, GLT8D1, GNAS,

		GNS, GRPEL1, GSN, GSPT1, GTF3C3, HEATR1, HGF, HIPK2, HMGN5, IL11, INA, INTS6, KAT2B, KIAA1324, KLHL1, LAPTM5, LMAN1, LPAR3, LRFN3, MAGED2, MAP3K2, MCL1, MET, MIA2, MPZL1, MTUS2, NBEA, NCS1, NENF, NFATC2IP, NIPSNAP1, NRDE2, NXPE3, OSBPL8, PCDH7, PDCD11, PDGFRA, PEBP1, PFKFB2, PHACTR2, PIAS2, PLAUR, PMAIP1, PNMA2, POLR1E, PPP2R2A, PSAP, PSIP1, PTPRC, RAB2A, RAB30, RAC2, RANBP9, RELB, RHOBTB3, RPS19, RRP12, RSL1D1, RYK, SCAMP1, SCD, SDC1, SEC24D, SEMA3C, SERPINB1, SGSM2, SH3BGRL, SLC17A5, SLC25A13, SLC7A11, SMURF1, SRD5A1, SRSF1, STK17A, TACSTD2, TAF1D, TBC1D19, TBC1D5, TCEA2, TGM2, THRA, TLK1, TMPO, TNFRSF10B, TRAK1, TRAPPC13, TRIM37, TRPC1, TSN, TYRO3, UBL3, UFSP2, WASL, WDR1, ZFAND6, ZNF148
5	hsa-mir-34a-5p	ACLY, ACVR1B, ADAMTS2, ADD2, AGTPBP1, ARG2, ARHGAP26, ARHGAP29, ARHGEF26, ARHGEF9, ARL1, ASB4, BTG2, CDK18, CDS2, CENPC, CENPI, CIB2, CITED2, CLCN4, CLGN, CYP4F2, DDX10, DHRS2, DHX40, DNASE2, DYNC2LI1, EEF1D, EHD1, ETV5, FAM222B, FECH, FGD6, FOXJ3, FOXN3, GM2A, GNAQ, GNAS, GOLGA5, GSN, GTF3C3, HEATR1, HIPK2, HNF4G, HSD11B2, IKZF4, INA, KAT2B, KIAA1324, KLF12, KLHL18, LAPTM5, LMAN1, LPXN, LZTFL1, MCL1, MET, MPHOSPH9, NENF, NFATC2IP, NIPSNAP1, NR6A1, NRDE2, NXPE3, PCGF2, PDGFRA, PEX19, PHF7, PLAUR, PMAIP1, PPIF, PPP1CA, PPP1R13L, PRKCH, PSAP, PTCH1, RAC2, RANBP9, RELB, RHOBTB3, RRAGD, RRP12, RWDD2A, SCD, SDC1, SEMA3C, SERPINB1, SGSM2, SH3BGRL, SH3BP2, SIRPA, SIRT2, SLC17A5, SLC25A13, SLC25A36, SLC29A3, SRSF1, STMN3, SYT13, TACSTD2, TCEA2, TGM2, THRA, TMEM39A, TMPO, TNFRSF10B, TRAK1, TRAPPC13, TRIM37, TRPC1, TSN, TYRO3, UBL3, UFSP2, VPS33A, YIPF2, ZNF280B, ZNF358
6	hsa-mir-27a-3p	ABAT, ACLY, ADAMTS2, ADD2, AKAP11, ALDH1A2, APC, APOBEC2, ARHGEF26, ARHGEF9, ASB4, ATP6V1A, BTBD1, BTG2, CAPZA1, CASQ2, CBFA2T2, CCL21, CD2, CDC42EP3, CDK18, CLCN4, COX11, CPD, CTNNA2, CYP2U1, DCN, DDX18, DHRS2, DOCK10, DYNC2LI1, EDN3, EEF1D, EHD1, ENTPD3, ERAP1, ERGIC3, ETV7, FAM222B, FGD6, FOXJ3, FOXN3, FRMD4B, GABRG2, GM2A, GNAS, GNG3, GNRHR, GNS, GSPT1, GTF3C3, HADH, HGF, HIPK2, IAPP, KLHL1, MAGED2, MAML3, MCL1, MET, MPHOSPH9, NCS1, NEBL, NR6A1, NRCAM, NXPE3, OSBPL8, OXR1, PCDH7, PDX1, PHACTR2, PMAIP1, PPIF, PPP1CA, PPP1R13L, PPP1R1A, PRKCH, PSAP, RAB30, RAP1GAP2, RCAN2, RCBTB1, RELB, RRAGD, RRP12, SEC24D, SEMA3C, SEMA5A, SGSM2, SH3BP2, SLC19A1, SLC1A2, SLC30A9, SLC7A11, SMAD9, SMURF1, SNX2, SRSF1, TBC1D19, TCEA2, TGFBR3, TGM2, THBS2, THRA, TMEM39A, TMEM59, TREML2, TRIM37, TRIP10, TRPM3, TTLL4, UBL3, WASL, WDR1, WT1, ZMAT4, ZNF148
7	hsa-let-7b-5p	ABCC4, ABCC8, ABHD10, ACLY, ACVR1B, ADCYAP1, ADD2, AGTPBP1, AKAP11, APC, ARG2, ARHGAP26, ARHGAP29, ARHGEF9, ARL1, ASAH1, ATP2B2, ATP6V1A, BTBD1, BTG2, CACNA2D1, CADM1, CASQ2, CDC14B, CDC42EP3, CENPC, CENPI, CITED2, CLCN4, CLGN, CPD, CRLF3, CYP4F2, DDX10, DDX18, DOCK5, EDN3, EEF1D, ERGIC3, ETV5, FAM222B, FECH, FGD6, FLII, FOXJ3, FOXN3, FRMD4B, GABRG2, GNAQ, GNAS, GNS, GRPEL1, GSN, GSPT1, HADH, HIPK2, HLF, HMGN5, INA, INTS6, ITGAX, KLF12, KLHL1, KLHL18, KLHL24, LPAR3, LPXN, MAGED2, MAML3, MAP3K2, MCL1, MDM1, MET, MPHOSPH9, MREG, MRPL3, MTUS2, NBEA, NDUFA10, NFATC2IP, NPC1, NR6A1, NXPE3, OLIG2, OSBPL8, OXR1, PADI2, PCDH7, PCGF2, PCMT1, PDCD11, PDGFRA, PDLIM4, PEX19, PFKFB2, PFKFB4, PGRMC1, PHACTR2, PIAS2, PLAUR, PMAIP1, PNMA2, POLR1E, PPIF, PPP1CA, PPP1R13L, PPP2R2A, PRKRA, PSAP, PTPN3, RAB30, RAC2, RAP1GAP2, RCAN2, RELB, RHOBTB3, RNF6, RRAGD, RRP12, RSL1D1, RWDD2A, SCAMP1, SCD, SEC24D, SEMA3C, SEMA5A, SLC17A5, SLC19A1, SLC25A13, SLC25A36, SLC29A3, SLC7A11, SMAD9, SMURF1, SNTB1, SNX2, SPTBN4, SRD5A1, SRSF1, STK17A, SYDE1, TBC1D19, TBC1D5, TEAD4, TGFBR3, TGM2, THRA, TLK1, TMPO, TNFRSF10B, TNFRSF14, TRAK1, TRIP10, TRPC1, TSN, TTLL4, UBL3, VPS33A, VRK3, WASL, WDR1, ZFAND6, ZNF148, ZNF280B
8	hsa-mir-129-2-3p	ABCC8, ACLY, ADAM28, ADD2, APPL2, ARHGAP26, ARHGAP29, BTG2, CACNA1D, CACNA2D1, CD2, CDC42EP3, CDK18, CDS2, CENPI, CITED2, CLGN, CLN5, CORO2A, CPD, CYHR1, DCN, DHRS2, DOCK10, DVL2, EEF1D, EHD1, EIF2AK2, ENTPD3, ERAP1, ETV7, EXTL1, FBXO7, FOXJ3, GNAQ, GNAS, HADH, HEATR1, HNF4G, KAT2B, KIAA1324, LPXN, LRFN3, MAML3, MCL1, MREG, NXPE3, PCDH7, PEBP1, PFKFB2, PFKFB4, PHACTR2, PHF7, PID1, PLAUR, PLEKHA4, PMAIP1, POLR1E, PPP1CA, PPP1R13L, PPP2R2A, PTCH1, PTGES, RAC2, RANBP1, RAP1GAP2, RCAN2, RSL1D1, SCAMP1,

		SEMA3A, SEMA5A, SERPINB1, SH3BGRL, SLC17A5, SLC25A36, SLC7A11, SNTB1, SNX2, SRD5A1, SRSF1, STMN3, TEAD4, TGFBR3, THBS2, THRA, TMEM59, TMPO, TNFRSF10B, TNFRSF14, TRAPPC13, TRIP10, TRPM3, TSN, UNC93B1, WASL, WDR1, WNT4, ZNF280B
9	hsa-mir-23b-3p	ABCC4, ACLY, ADAM28, AKAP11, ARHGAP29, ARHGEF9, ATP6V1A, BTBD1, BTG2, CAPZA1, CDS2, CITED2, CLGN, CPD, CRLF3, DHRS2, DHX40, DNASE2, DOCK10, DYNC2LI1, EEF1D, EHD1, EPB41L5, ERAP1, ERGIC3, ETV5, FAM222B, FBXO7, FLII, FOXJ3, GART, GNS, GRPEL1, GSN, GSPT1, GTF3C3, HIPK2, KLHL24, LAPTM5, LMAN1, LZTFL1, MAGED2, MAK16, MAP3K2, MCL1, MET, MRPL3, NENF, NIPSNAP1, NPC1, NR6A1, NRCAM, NXPE3, OSBPL8, PCDH7, PCGF2, PCMT1, PEBP1, PGRMC1, PMAIP1, PNMA2, POLR1E, PPIF, PPP1CA, PSAP, PTGES, RANBP1, RCBTB1, RELB, RHOBTB3, RRAGD, RYK, SCD, SERPINB1, SH3BGRL, SLAMF7, SLC19A1, SLC25A36, SNX2, SRSF1, SYDE1, TAF1D, TGFBR3, TGM2, THBS2, TLK1, TMPO, TNFRSF10B, UBL3, VRK3, ZNF148, ZNF358
10	hsa-mir-107	RNF6, RRAGD, RRP12, RSL1D1, RWDD2A, SCAMP1, SCD, SEC24D, SEMA3C, SEMA5A, SLC17A5, SLC19A1, SLC25A13, SLC25A36, SLC29A3, SLC7A11, SMAD9, SMURF1, SNTB1, SNX2, SPTBN4, SRD5A1, SRSF1, STK17A, SYDE1, TBC1D19, TBC1D5, TEAD4, TGFBR3, TGM2, THRA, TLK1, TMPO, TNFRSF10B, TNFRSF14, TRAK1, TRIP10, TRPC1, TSN, TTLL4, UBL3, VPS33A, VRK3, WASL, WDR1, ZFAND6, ZNF148, ZNF280B

Table S33. Top 10 ranked microRNAs regulate the transcriptional expression of DEGs for diabetic pancreatic tissue and diabetic liver tissue.

Rank	MicroRNA	Gene
1	hsa-mir-1-3p	APC, ARHGAP29, ARL1, ASAH1, BTBD1, CAPZA1, CDC42EP3, CDK5RAP2, CRYZL1, DDX18, DOCK10, EPB41L5, ETV5, GM2A, GNAQ, GNAS, HMGN5, LMBRD1, MAK16, MAP3K2, MCL1, MPHOSPH9, NBEA, NPC1, NR6A1, PADI2, PCDH7, PDGFRA, PDLIM4, PFKFB2, PHACTR2, PSIP1, RAB2A, RHOBTB3, SCAMP1, SEC24D, SERPINB1, SLC1A2, SLC25A36, SLC7A11, SNX13, STK17A, TACSTD2, TLK1, TSN, TTLL4, UNC93B1, ZFAND6, ZNF148
2	hsa-mir-155-5p	APC, ARHGEF9, ARL1, BTBD1, CDC14B, CDC42EP3, CDKL5, DDX18, DOCK10, EEF1D, ETV5, GNAS, GSPT1, GTF3C3, HGF, HMGN5, MAP3K2, MCL1, NBEA, PCDH7, PDGFRA, PFKFB2, PHACTR2, PSIP1, PTPRC, RAB2A, RHOBTB3, SCAMP1, SEC24D, SERPINB1, SGSM2, SLC7A11, STK17A, TACSTD2, TLK1, TRAK1, TSN, ZFAND6, ZNF148
3	hsa-mir-124-3p	NRG1, RAB2A, TSN, CDC14B, ARHGAP29, PHACTR2, SEC24D, MPHOSPH9, CDC42EP3, SNX13, ARHGEF26, SLC25A36, EPB41L5, PTPRC, PTPRC, SCAMP1, ZFAND6, EEF1D, EEF1D, CAPZA1, PCMT1, PFKFB2, PPP1R1A, VPS33A, NPC1, MCL1, SLC7A11, ZNF148, RHOBTB3, STK17A, OXR1, PCDH7, MAP3K2, NBEA, HSD11B2, TRAK1, GM2A, MAK16, HMGN5, CRYZL1
4	hsa-let-7b-5p	APC, ARHGAP29, ARHGEF9, ARL1, ATP2B2, CDC42EP3, DDX18, EEF1D, ERGIC3, ETV5, FGD6, GNAS, GSPT1, HMGN5, MAML3, MAP3K2, MCL1, MPHOSPH9, NBEA, NR6A1, OXR1, PCDH7, PCGF2, PDGFRA, PFKFB2, PHACTR2, RHOBTB3, SCAMP1, SEC24D, SLC25A36, SLC7A11, TRAK1, TSN, TTLL4, VPS33A, ZNF148
5	hsa-mir-34a-5p	SERPINB1, MCL1, PDGFRA, TSN, GTF3C3, ASB4, SYT13, MPHOSPH9, GNAS, EEF1D, SLC25A36, ARHGEF26, ARL1, ARHGEF9, ARHGAP29, VPS33A, SGSM2, NR6A1, GNAQ, RHOBTB3, HSD11B2, FGD6, TRAK1, TACSTD2, GM2A, ZNF358, ETV5, PCGF2
6	hsa-mir-101-3p	ARL1, ASAH1, ATP2B2, BTBD1, CBFA2T2, CDC14B, EPB41L5, ETV5, GNAQ, MAK16, MAML3, MAP3K2, MCL1, MPHOSPH9, NRG1, OXR1, PCDH7, PDGFRA, PDLIM4, PHACTR2, SCAMP1, SLC1A2, SLC7A11, TLK1, TSN, ZFAND6
7	hsa-mir-15a-5p	APC, CAPZA1, CDC14B, EEF1D, FRMD4B, GNAQ, GSPT1, HGF, MCL1, NBEA, NR6A1, OXR1, PCGF2, PCMT1, PHACTR2, RHOBTB3, SLC25A36, TACSTD2, TLK1, TRAK1, TSN, ZFAND6
8	hsa-mir-26a-5p	ARHGAP29, ARL1, CAPZA1, CDKL5, FRMD4B, GNAQ, GSPT1, HGF, MAP3K2, MCL1, PCDH7, PCMT1, PFKFB2, PSIP1, RHOBTB3, SCAMP1, SEC24D, SLC25A36, SLC7A11, TLK1, ZFAND6, ZNF148
9	hsa-mir-181a-5p	APC, ARHGAP29, ARL1, DOCK10, GNAQ, GSPT1, MAP3K2, MCL1, NR6A1, PCGF2, PDGFRA, PFKFB2, PHACTR2, PSIP1, SCAMP1, SLC1A2, SLC25A36, SLC7A11, TACSTD2, TRAK1, ZFAND6, ZNF148
10	hsa-let-7a-5p	APC, ARHGEF9, ATP2B2, BTBD1, DDX18, ERGIC3, FGD6, GSPT1, MAML3, MAP3K2, NPC1, NR6A1, OXR1, PCDH7, PFKFB2, PHACTR2, RHOBTB3, TRAK1, TSN, TTLL4, VPS33A

Table S34. Top 10 ranked microRNAs regulate the transcriptional expression of DEGs for diabetic pancreatic tissue and diabetic heart tissue.

Rank	MicroRNA	Gene
1	hsa-mir-16-5p	ACLY, AKAP11, ARHGEF9, CBFA2T2, CDS2, CENPC, CITED2, CYP2U1, DOCK5, EEF1D, FGD6, FLII, FRMD4B, GART, GNAQ, HADH, HIPK2, KLHL24, LAPTM5, MAGED2, MRPL3, NEBL, NPC1, PCDH7, PIGA, PLAUR, PRKRA, PSIP1, PTCH1, RAB30, RELB, RRP12, SEMA3A, SEMA3C, SMAD9, TAF1D, TGFBR3, TGM2, TRAK1, WDR1
2	hsa-mir-124-3p	ACLY, AKAP11, CENPC, CITED2, CYP2U1, DYNC2LI1, EEF1D, FLII, GART, HADH, HIPK2, IAPP, KLHL24, LAPTM5, MRPL3, NEBL, NPC1, NRG1, OXR1, PCDH7, PDCD11, PFKFB2, PIGA, PLAUR, PLEKHA4, PPP1R13L, RAB30, RANBP1, RRP12, SEMA3A, SEMA3C, SMAD9, TAF1D, TEAD4, TGFBR3, TGM2, TRAK1, TRPC1, WDR1, ZNF280B
3	hsa-mir-1-3p	AKAP11, CDK5RAP2, CDS2, CENPC, CITED2, CYP2U1, DYNC2LI1, ERAP1, GNAQ, HADH, HIPK2, KLHL18, KLHL24, LAPTM5, MAGED2, MRPL3, NCS1, NPC1, PCDH7, PDCD11, PDPN, PFKFB2, PIGA, PLAUR, PPP1CA, PSIP1, RAB30, RANBP1, RRP12, SEMA3A, SEMA3C, SLC1A2, SMAD9, TAF1D, TEAD4, TGFBR3, TGM2
4	hsa-mir-27a-3p	ACLY, AKAP11, ARHGEF9, CBFA2T2, CDK18, CYP2U1, DYNC2LI1, EEF1D, ERAP1, FGD6, FRMD4B, HADH, HIPK2, IAPP, MAGED2, MAML3, NCS1, NEBL, OXR1, PCDH7, PPP1CA, PPP1R13L, RAB30, RELB, RRP12, SEMA3C, SEMA5A, SLC1A2, SMAD9, TGFBR3, TGM2, TRPM3, WDR1, ZMAT4
5	hsa-let-7b-5p	ACLY, ARHGEF9, CENPC, CITED2, DOCK5, EEF1D, FGD6, FLII, HIPK2, KLHL24, MAML3, OXR1, PCDH7, PDCD11, PFKFB2, PPP1CA, PPP1R13L, PRKRA, RAB30, RELB, SEMA3C, SMAD9, TEAD4, TGFBR3, TGM2, TRAK1, TRPC1, WDR1, ZNF280B
6	hsa-mir-155-5p	ACLY, AKAP11, ARHGEF9, CITED2, CYP2U1, DYNC2LI1, EEF1D, FLII, GART, HIPK2, LAPTM5, MAGED2, NCS1, PCDH7, PDCD11, PFKFB2, PLAUR, PSIP1, RAB30, RELB, RRP12, SEMA3C, TAF1D, TGM2, TRAK1, TRPC1, WDR1
7	hsa-mir-20a-5p	ACLY, AKAP11, CITED2, CYP2U1, ERAP1, FLII, KLHL18, MAGED2, MRPL3, NEBL, NRG1, OXR1, PFKFB2, RAB30, SEMA3A, TGFBR3, WDR1, ZNF280B
8	hsa-mir-26b-5p	ABCA6, AKAP11, CDK18, DYNC2LI1, ERAP1, FRMD4B, HADH, HIPK2, IAPP, ITGAX, KLHL24, NEBL, PCDH7, PFKFB2, PIGA, RAB30, RELB, ZMAT4
9	hsa-mir-27b-3p	ACLY, AKAP11, ARHGEF9, CDK18, DYNC2LI1, EEF1D, FGD6, HADH, HIPK2, MAML3, NEBL, OXR1, PPP1CA, RAB30, SLC1A2, SMAD9, TGFBR3, WDR1
10	hsa-mir-147a	CDK5RAP2, CITED2, DYNC2LI1, ERAP1, FGD6, GNAQ, HIPK2, KLHL24, MAGED2, NCS1, PCDH7, PFKFB2, PLAUR, PSIP1, PTCH1, RAB30, SEMA5A, TGM2, TRPC1

Table S35. Top 10 ranked microRNAs regulate the transcriptional expression of 16-DEGs for common diabetic pancreatic, diabetic heart and diabetic liver tissues.

Rank	MicroRNA	Gene
1	hsa-mir-16-5p	TRAK1, CBFA2T2, EEF1D, FRMD4B, ARHGEF9, NPC1, GNAQ, PSIP1, PCDH7, FGD6
2	hsa-mir-27a-3p	ARHGEF9, CBFA2T2, EEF1D, FGD6, FRMD4B, MAML3, OXR1, PCDH7, SLC1A2
3	hsa-let-7a-5p	ARHGEF9, FGD6, MAML3, NPC1, OXR1, PCDH7, PFKFB2, TRAK1
4	hsa-let-7b-5p	EEF1D, PFKFB2, ARHGEF9, PCDH7, FGD6, TRAK1, MAML3
5	hsa-mir-101-3p	CBFA2T2, MAML3, SLC1A2, GNAQ, NRG1, OXR1, PCDH7
6	hsa-mir-1-3p	PCDH7, PSIP1, SLC1A2, PFKFB2, CDK5RAP2, NPC1, GNAQ
7	hsa-mir-124-3p	NRG1, EEF1D, PFKFB2, NPC1, OXR1, PCDH7, TRAK1
8	hsa-mir-103a-3p	EEF1D, FRMD4B, CDK5RAP2, NPC1, GNAQ, PCDH7, TRAK1
9	hsa-mir-122-5p	PFKFB2, CBFA2T2, CDK5RAP2, GNAQ, PCDH7, TRAK1
10	hsa-mir-155-5p	PSIP1, TRAK1, EEF1D, PFKFB2, ARHGEF9, PCDH7

Table S36: MicroRNA-hub gene interaction

Sr. No.	Gene		Target Micro-RNA
1.	ARHGEF9	12	hsa-mir-567, hsa-mir-297, hsa-mir-223-5p, hsa-mir-574-5p, hsa-mir-3177-5p, hsa-mir-4711-3p, hsa-mir-511-3p, hsa-mir-6817-3p, hsa-mir-6867-5p, hsa-mir-6873-3p, hsa-mir-7110-3p
2.	CBFA2T2	30	hsa-mir-92a-3p, hsa-mir-101-3p, hsa-mir-106b-5p, hsa-mir-490-3p, hsa-mir-512-5p, hsa-mir-510-5p, hsa-mir-619-3p, hsa-mir-377-5p, hsa-mir-455-3p, hsa-mir-3202, hsa-mir-4257, hsa-mir-4252, hsa-mir-3714, hsa-mir-3929, hsa-mir-4478, hsa-mir-4419b, hsa-mir-4533, hsa-mir-2467-3p, hsa-mir-5690 hsa-mir-5694, hsa-mir-1273g-3p, hsa-mir-6086, hsa-mir-6499-3p, hsa-mir-6511a-5p, hsa-mir-605-3p, hsa-mir-1910-3p, hsa-mir-6807-5p, hsa-mir-7160-5p, hsa-mir-6516-5p, hsa-mir-7977
3.	CDKRAP2	3	hsa-miR-1301-3p, hsa-miR-149-5p, has-miR-324-3p
4.	EEF1D	4	hsa-miR-218-5p, hsa-miR-149-5p, hsa-miR-186-5p, hsa-miR-93-3p
5.	FGD6	41	hsa-mir-98-5p, hsa-mir-129-5p, hsa-mir-143-3p, hsa-mir-150-5p, hsa-mir-335-5p, hsa-mir-524-5p, hsa-mir-520d-5p, hsa-mir-508-3p, hsa-mir-802, hsa-mir-186-3p, hsa-mir-135b-3p, hsa-mir-1289, hsa-mir-1292-5p, hsa-mir-548u, hsa-mir-3198, hsa-mir-4294, hsa-mir-4309, hsa-mir-3692-3p, hsa-mir-3918, hsa-mir-4471, hsa-mir-4534, hsa-mir-4663, hsa-mir-4685-5p, hsa-mir-4698, hsa-mir-4708-5p, hsa-mir-4770, hsa-mir-1273f, hsa-mir-5186, hsa-mir-6088, hsa-mir-6514-5p, hsa-mir-6753-5p, hsa-mir-6807-5p, hsa-mir-6837-5p, hsa-mir-7160-5p, hsa-mir-7161-5p, hsa-mir-8055, hsa-mir-8059, hsa-mir-8077, hsa-mir-8082, hsa-mir-8083, hsa-mir-8087
6.	FRMD4B	8	hsa-mir-98-5p, hsa-mir-9-5p, hsa-mir-335-5p, hsa-mir-526b-5p, hsa-mir-578, hsa-mir-3672, hsa-mir-4524a-3p, hsa-mir-6864-3p
7.	GNAQ	26	hsa-mir-25-3p, hsa-mir-32-5p, hsa-mir-92a-3p, hsa-mir-129-5p, hsa-mir-128-3p, hsa-mir-142-3p, hsa-mir-363-3p, hsa-mir-367-3p, hsa-mir-501-5p, hsa-mir-92b-3p, hsa-mir-548b-3p, hsa-mir-653-5p, hsa-mir-767-5p, hsa-mir-500a-5p, hsa-mir-590-3p, hsa-mir-3130-3p, hsa-mir-3613-3p, hsa-mir-3653-3p, hsa-mir-3658, hsa-mir-4511, hsa-mir-4668-3p, hsa-mir-4775, hsa-mir-5579-3p, hsa-mir-5589-3p, hsa-mir-6885-3p
8.	MAML3	22	hsa-mir-101-3p, hsa-mir-144-3p, hsa-mir-452-3p, hsa-mir-485-3p, hsa-mir-505-3p, hsa-mir-579-3p, hsa-mir-548c-3p, hsa-mir-499a-3p, hsa-mir-576-3p, hsa-mir-1272, hsa-mir-3682-3p, hsa-mir-4424, hsa-mir-4477b, hsa-mir-4679, hsa-mir-499b-3p, hsa-mir-664b-3p, hsa-mir-5684, hsa-mir-5696, hsa-mir-539-3p, hsa-mir-1255b-2-3p, hsa-mir-1273g-3p, hsa-mir-651-3p
9.	NPC1	03	hsa-let-7a-5p, hsa-mir-33a-5p, hsa-mir-335-5p
10.	NRG1	02	hsa-mir-124-3p,hsa-mir-125a-3p
11.	OXR1	22	hsa-let-7b-5p, hsa-mir-17-5p, hsa-mir-20a-5p, hsa-mir-93-5p, hsa-mir-200b-3p, hsa-mir-144-3p, hsa-mir-106b-5p, hsa-mir-20b-5p, hsa-mir-519d-3p, hsa-mir-567, hsa-mir-586, hsa-mir-875-5p, hsa-mir-1224-5p, hsa-mir-3144-3p, hsa-mir-3915, hsa-mir-3928-3p, hsa-mir-642b-3p, hsa-mir-4689, hsa-mir-4781-3p, hsa-mir-642a-3p, hsa-mir-6730-3p, hsa-mir-6858-5p
12.	PCDH7	20	hsa-mir-25-3p, hsa-mir-192-5p, hsa-mir-1-3p, hsa-mir-338-3p, hsa-mir-329-3p, hsa-mir-603, hsa-mir-362-3p, hsa-mir-4261, hsa-mir-3688-3p, hsa-mir-3941, hsa-mir-4530, hsa-mir-4789-5p, hsa-mir-4789-3p, hsa-mir-5011-5p, hsa-mir-1277-5p, hsa-mir-6512-3p, hsa-mir-190a-3p, hsa-mir-6720-5p, hsa-mir-8076, hsa-mir-8485
13.	PFKFB2	31	hsa-mir-17-5p, hsa-mir-20a-5p, hsa-mir-21-5p, hsa-mir-93-5p, hsa-mir-106a-5p, hsa-mir-181a-5p, hsa-mir-122-5p, hsa-mir-106b-5p, hsa-mir-376a-3p, hsa-mir-377-3p, hsa-mir-382-5p, hsa-mir-20b-5p, hsa-mir-491-5p, hsa-mir-526b-3p, hsa-mir-519d-3p, hsa-mir-552-3p, hsa-mir-125a-3p, hsa-mir-548n, hsa-mir-2110, hsa-mir-548t-5p, hsa-mir-4271, hsa-mir-4481, hsa-mir-4693-3p, hsa-mir-4725-3p, hsa-mir-4745-5p, hsa-mir-1185-2-3p, hsa-mir-1185-1-3p, hsa-mir-548az-5p, hsa-mir-585-5p, hsa-mir-1296-3p, hsa-mir-6780b-5p
14.	PSIP1	4	hsa-mir-1-3p, hsa-mir-155-5p, hsa-mir-375, hsa-mir-193b-3p

15.	SLC1A2		hsa-mir-24-3p, hsa-mir-30a-3p, hsa-mir-31-5p, hsa-mir-200c-3p, hsa-mir-30e-3p, hsa-mir-501-5p, hsa-mir-564, hsa-mir-663a, hsa-mir-15a-3p, hsa-mir-30c-2-3p, hsa-mir-30d-3p, hsa-mir-30b-3p, hsa-mir-149-3p, hsa-mir-30c-1-3p, hsa-mir-497-3p, hsa-mir-500a-5p, hsa-mir-455-3p, hsa-mir-1324, hsa-mir-1908-5p, hsa-mir-1910-5p, hsa-mir-3122, hsa-mir-3176, hsa-mir-3190-5p, hsa-mir-4284, hsa-mir-3689a-3p, hsa-mir-3689b-3p, hsa-mir-3913-5p, hsa-mir-3922-3p, hsa-mir-3689c, hsa-mir-4728-5p, hsa-mir-4743-5p, hsa-mir-548as-3p, hsa-mir-450a-1-3p, hsa-mir-6513-5p, hsa-mir-383-3p, hsa-mir-887-5p, hsa-mir-6776-3p, hsa-mir-6779-5p, hsa-mir-6780a-5p, hsa-mir-6785-5p, hsa-mir-6787-5p, hsa-mir-6788-5p, hsa-mir-6799-5p, hsa-mir-6883-5p, hsa-mir-7106-5p, hsa-mir-1273h-5p, hsa-mir-7977
16.	TAK1	47	hsa-mir-15a-5p, hsa-mir-16-5p, hsa-mir-93-5p, hsa-mir-15b-5p, hsa-mir-195-5p, hsa-mir-155-5p, hsa-mir-324-3p, hsa-mir-424-5p, hsa-mir-497-5p, hsa-mir-503-5p, hsa-mir-646, hsa-mir-374b-3p, hsa-mir-711, hsa-mir-4524a-5p, hsa-mir-4524b-5p, hsa-mir-6838-5p

Table S37. DEGs of all four groups [Group: 1, (Pancreas), Group:2 (Heart), Group:3 (Liver), Group:4 (Common DEGs of four groups) targeted by hsa-miR-1-3p.

Sample Organ	Total DEGs	DEGs
16 DEGs, Heart, Liver, Pancreas	4	GNAQ, PCDH7, CDK5RAP2, NPC1
16 DEGs, Heart, Liver	3	SLC1A2 PFKFB2, PSIP1
Heart Pancreas	14	HADH, CITED2, PDCD11, TGFBR3, AKAP11, MAGED2, KLHL18, PLAUR, MRPL3, HIPK2, TGM2, SEMA3C, PIGA, RRP12
Liver Pancreas	4	FLII, DOCK5, PDCD11, TGFBR3
Heart	16	TAF1D, SEMA3A, RAB30, PDPN, CENPC, CDS2, DYNC2LI1, SMAD9, NCS1, LAPTM5, ERAP1, CYP2U1, RANBP1, TEAD4, KLHL24, PPP1CA
Liver	25	SERPINB1 RAB2A, LMBRD1, PDLIM4, DOCK10, ARHGAP29, TACSTD2, CDC42EP3, MPHOSPH9, TTLL4, SNX13, EPB41L5, CRYZL1, PDGFRA, GM2A, MCL1, SCAMP1, UNC93B1, MAK16, TSN, GNAS, ASAH1, NBEA, STK17A, MAP3K2
Pancreas	50	TMPO, BTG2, PCGF2, SYDE1, SRSF1, CACNA2D1, RSL1D1, GSN, PPIF, SPTBN4, MREG, FOXJ3, EDN3, PIAS2, PGRMC1, GABRG2, PMAIP1, RCAN2, SCD, ADCYAP1, GSPT1, PTPN3, TRAK1, TNFRSF10B, CENPI, DHX40, LPXN, SLC17A5, ADD2, HLF, GNS, SLC29A3, TNFRSF14, RNF6, UBL3, VRK3, ITGAX, YIPF2, EEF1D, ABCC4, CYP4F2, FRMD4B, GTF3C3, PCMT1, CADM1, WDR1, IL11, SNTB1, ACLY, THRA

Table S38. DEGs of all four groups [Group: 1, (Pancreas), Group: 2 (Heart), Group: 3 (Liver), Group: 4 (Common DEGs of three groups) targeted by hsa-let-7b-5p.

Sample Organ	Total DEGs	DEGs
16 DEGs, Heart, Liver, Pancreas	1	OXR1
16 DEGs, Heart, Liver	7	MAML3, FGD6, PFKFB2, TRAK1, PCDH7, ARHGEF9, EEF1D
Heart Pancreas	4	FLII, DOCK5, PDCD11, TGFBR3
Liver Pancreas	8	GSPT1, PDGFRA, SCAMP1, ERGIC3, GNAS, DDX18, ZNF148, NR6A1
Heart	17	RAB30, HIPK2, TGM2, PPP1R13L, PRKRA, CITED2, SEMA3C, CENPC, ZNF280B, RELB, TEAD4, KLHL24, SMAD9, PPP1CA, WDR1, TRPC1, ACLY
Liver	22	RHOBTB3, VPS33A, BTBD1, PCGF2, MCL1, SEC24D, NPC1, TSN, HMGN5, APC, PHACTR2, ARHGAP29, SLC7A11, NBEA, ATP2B2, CDC42EP3, ARL1, SLC25A36, MPHOSPH9, MAP3K2, TTLL4, ETV5
Pancreas	13	TBC1D19, PMAIP1, FAM222B, SCD, BTG2, TNFRSF10B, WASL, ARHGAP26, NDUFA10, SLC25A13, PPP2R2A, DDX10, PGRMC1

Table S39. DEGs of all four groups [Group: 1, (Pancreas), Group: 2 (Heart), Group: 3 (Liver), Group: 4 (Common DEGs of four groups) targeted by *hsa-mir-155-5p*.

Sample Organ	Total DEGs	DEGs
16 DEGs, Heart, Liver, Pancreas	2	TRAK1, PSIP1
16 DEGs, Heart, Liver	4	PFKFB2, PCDH7, ARHGEF9, EEF1D
Heart Pancreas	4	RAB30, TGM2, CYP2U1, PLAUR
Liver Pancreas	9	RAB2A, BTBD1, SCAMP1, APC, PHACTR2, GNAS, SLC7A11, TACSTD2, ZNF148
Heart	17	LAPTM5, FLII, TAF1D, HIPK2, CITED2, PDCD11, SEMA3C, GART, RELB, MAGED2, AKAP11, DYNC2LI1, RRP12, WDR1, TRPC1, ACLY, NCS1
Liver	24	SERPINB1, ZFAND6, RHOBTB3, GSPT1, PDGFRA, SGSM2, MCL1, SEC24D, DOCK10, HGF, TSN, HMGN5, NBEA, DDX18, STK17A, CDC42EP3, ARL1, TLK1, CDKL5, GTF3C3, MAP3K2, CDC14B, ETV5, PTPRC
Pancreas	18	SCD, PEBP1, GRPEL1, MIA2, DHX40, MPZL1, INTS6, INA, SRSF1, UBL3, ALDH1A2, PPP2R2A, NFATC2IP, EHD1, ABCC4, DDX10, CPD, F5

Table S40. DEGs of all four groups [Group: 1, (Pancreas), Group: 2 (Heart), Group: 3 (Liver), Group: 4 (Common DEGs of four groups) targeted by *hsa-mir-124-3p*.

Sample Organ	Total DEGs	DEGs
16 DEGs, Heart, Liver, Pancreas	1	NRG1
16 DEGs, Heart, Liver	6	PFKFB2, TRAK1, PCDH7, NPC1, OXR1, EEF1D
Heart Pancreas	7	FLII, PLEKHA4, CYP2U1, PPP1R13L, HADH, KLHL24, TRPC1
Liver Pancreas	12	EPB41L5, RAB2A, SEC24D, TSN, PHACTR2, ARHGAP29, CDC42EP3, ARHGEF26, SLC25A36, MPHOSPH9, CDC14B, SNX13
Heart	26	LAPTM5, TAF1D, MRPL3, SEMA3A, RAB30, HIPK2, TGM2, NEBL, IAPP, CITED2, RANBP1, PDCD11, SEMA3C, CENPC, GART, ZNF280B, PIGA, TGFBR3, AKAP11, DYNC2LI1, TEAD4, SMAD9, RRP12, WDR1, ACLY, PLAUR
Liver	19	ZFAND6, HSD11B2, VPS33A, RHOBTB3, CRYZL1, GM2A, MCL1, PPP1R1A, SCAMP1, MAK16, HMGN5, SLC7A11, NBEA, STK17A, ZNF148, MAP3K2, CAPZA1, PCMT1, PTPRC
Pancreas	21	PTGES, FECH, MTUS2, DCC, RYK, MIA2, EIF2AK2, SLC17A5, INA, CLN5, GSN, PPIF, DVL2, TRIM37, APPL2, SH2D3A, PHF7, GNRHR, CORO2A, WNT4, IL11

Table S41. MicroRNAs enrichment (KEGG Pathway) analysis of top four common targeted MicroRNAs (hsa-let-7b-5p, hsa-miR-124-3p, hsa-miR-1-3p, hsa-miR-155-5p).

microRNA	Description	p-value	FDR	Genes	Count
hsa-let-7b-5p	**Cellular senescence**	0.00	0.00	CDK6/CCND1/CCND2/NRAS/CCNA1/AKT2/E2F2	7
hsa-let-7b-5p	**Melanoma**	0.00	0.00	CDK6/CCND1/NRAS/AKT2/E2F2	5
hsa-let-7b-5p	**Non-small cell lung cancer**	0.00	0.00	CDK6/CCND1/NRAS/AKT2/E2F2	5
hsa-let-7b-5p	**Glioma**	0.00	0.00	CDK6/CCND1/NRAS/AKT2/E2F2	5
hsa-let-7b-5p	**Chronic myeloid leukemia**	0.00	0.00	CDK6/CCND1/NRAS/AKT2/E2F2	5
hsa-let-7b-5p	**Epstein-Barr virus infection**	0.00	0.00	CDK6/CCND1/CCND2/CCNA1/AKT2/E2F2	6
hsa-let-7b-5p	**Human T-cell leukemia virus 1 infection**	0.00	0.00	CCND1/CCND2/NRAS/CCNA1/AKT2/E2F2	6
hsa-let-7b-5p	**Cell cycle**	0.00	0.00	CDK6/CCND1/CCND2/CCNA1/E2F2	5
hsa-let-7b-5p	**Measles**	0.00	0.00	CDK6/CCND1/CCND2/AKT2/TLR4	5
hsa-let-7b-5p	**Breast cancer**	0.00	0.00	CDK6/CCND1/NRAS/AKT2/E2F2	5
hsa-let-7b-5p	**Hepatitis C**	0.00	0.00	CDK6/CCND1/NRAS/AKT2/E2F2	5
hsa-let-7b-5p	**Acute myeloid leukemia**	0.00	0.00	CCND1/NRAS/CCNA1/AKT2	4
hsa-let-7b-5p	**Hepatitis B**	0.00	0.00	NRAS/CCNA1/AKT2/TLR4/E2F2	5
hsa-let-7b-5p	**MicroRNAs in cancer**	0.00	0.00	CDK6/CCND1/CCND2/NRAS/EZH2/E2F2	6
hsa-let-7b-5p	**Prolactin signaling pathway**	0.00	0.00	CCND1/CCND2/NRAS/AKT2	4
hsa-let-7b-5p	**Hepatocellular carcinoma**	0.00	0.00	CDK6/CCND1/NRAS/AKT2/E2F2	5
hsa-let-7b-5p	**Human papillomavirus infection**	0.00	0.00	CDK6/CCND1/CCND2/NRAS/CCNA1/AKT2	6
hsa-let-7b-5p	**Pancreatic cancer**	0.00	0.00	CDK6/CCND1/AKT2/E2F2	4
hsa-let-7b-5p	**PI3K-Akt signaling pathway**	0.00	0.00	CDK6/CCND1/CCND2/NRAS/AKT2/TLR4	6
hsa-let-7b-5p	**Kaposi sarcoma-associated herpesvirus infection**	0.00	0.00	CDK6/CCND1/NRAS/AKT2/E2F2	5
hsa-let-7b-5p	**Viral carcinogenesis**	0.00	0.00	CDK6/CCND1/CCND2/NRAS/CCNA1	5
hsa-let-7b-5p	**Small cell lung cancer**	0.00	0.00	CDK6/CCND1/AKT2/E2F2	4
hsa-let-7b-5p	**Prostate cancer**	0.00	0.00	CCND1/NRAS/AKT2/E2F2	4
hsa-let-7b-5p	**Endocrine resistance**	0.00	0.00	CCND1/NRAS/AKT2/E2F2	4
hsa-let-7b-5p	**Human cytomegalovirus infection**	0.00	0.00	CDK6/CCND1/NRAS/AKT2/E2F2	5
hsa-let-7b-5p	**Bladder cancer**	0.00	0.00	CCND1/NRAS/E2F2	3
hsa-let-7b-5p	**FoxO signaling pathway**	0.00	0.00	CCND1/CCND2/NRAS/AKT2	4
hsa-let-7b-5p	**Gastric cancer**	0.00	0.00	CCND1/NRAS/AKT2/E2F2	4
hsa-let-7b-5p	**Endometrial cancer**	0.00	0.00	CCND1/NRAS/AKT2	3
hsa-let-7b-5p	**p53 signaling pathway**	0.00	0.00	CDK6/CCND1/CCND2	3
hsa-let-7b-5p	**Proteoglycans in cancer**	0.00	0.00	CCND1/NRAS/AKT2/TLR4	4

hsa-let-7b-5p	**Colorectal cancer**	0.00	0.00	CCND1/NRAS/AKT2	3
hsa-let-7b-5p	**PD-L1 expression and PD-1 checkpoint pathway in cancer**	0.00	0.00	NRAS/AKT2/TLR4	3
hsa-let-7b-5p	**AGE-RAGE signaling pathway in diabetic complications**	0.00	0.00	CCND1/NRAS/AKT2	3
hsa-let-7b-5p	**Progesterone-mediated oocyte maturation**	0.00	0.00	CCNA1/CPEB4/AKT2	3
hsa-let-7b-5p	**AMPK signaling pathway**	0.00	0.00	CCND1/CCNA1/AKT2	3
hsa-let-7b-5p	**Thyroid hormone signaling pathway**	0.00	0.00	CCND1/NRAS/AKT2	3
hsa-let-7b-5p	**Apelin signaling pathway**	0.00	0.00	CCND1/NRAS/AKT2	3
hsa-let-7b-5p	**Alcoholic liver disease**	0.00	0.00	CCND1/AKT2/TLR4	3
hsa-let-7b-5p	**Thyroid cancer**	0.00	0.00	CCND1/NRAS	2
hsa-let-7b-5p	**Cushing syndrome**	0.00	0.00	CDK6/CCND1/E2F2	3
hsa-let-7b-5p	**JAK-STAT signaling pathway**	0.00	0.00	CCND1/CCND2/AKT2	3
hsa-let-7b-5p	**Influenza A**	0.00	0.00	CDK6/AKT2/TLR4	3
hsa-let-7b-5p	**Focal adhesion**	0.00	0.00	CCND1/CCND2/AKT2	3
hsa-let-7b-5p	**Hedgehog signaling pathway**	0.00	0.00	CCND1/CCND2	2
hsa-let-7b-5p	**Human immunodeficiency virus 1 infection**	0.00	0.00	NRAS/AKT2/TLR4	3
hsa-let-7b-5p	**Chemical carcinogenesis - receptor activation**	0.00	0.00	CCND1/NRAS/AKT2	3
hsa-let-7b-5p	**VEGF signaling pathway**	0.00	0.00	NRAS/AKT2	2
hsa-let-7b-5p	**Lipid and atherosclerosis**	0.00	0.00	NRAS/AKT2/TLR4	3
hsa-let-7b-5p	**Longevity regulating pathway - multiple species**	0.00	0.00	NRAS/AKT2	2
hsa-let-7b-5p	**GnRH secretion**	0.00	0.00	NRAS/AKT2	2
hsa-let-7b-5p	**Fc epsilon RI signaling pathway**	0.00	0.00	NRAS/AKT2	2
hsa-let-7b-5p	**Renal cell carcinoma**	0.00	0.00	NRAS/AKT2	2
hsa-let-7b-5p	**Central carbon metabolism in cancer**	0.00	0.00	NRAS/AKT2	2
hsa-let-7b-5p	**EGFR tyrosine kinase inhibitor resistance**	0.01	0.01	NRAS/AKT2	2
hsa-let-7b-5p	**B cell receptor signaling pathway**	0.01	0.01	NRAS/AKT2	2
hsa-let-7b-5p	**ErbB signaling pathway**	0.01	0.01	NRAS/AKT2	2
hsa-let-7b-5p	**Longevity regulating pathway**	0.01	0.01	NRAS/AKT2	2
hsa-let-7b-5p	**Choline metabolism in cancer**	0.01	0.01	NRAS/AKT2	2
hsa-let-7b-5p	**Chagas disease**	0.01	0.01	AKT2/TLR4	2
hsa-let-7b-5p	**Toll-like receptor signaling pathway**	0.01	0.01	AKT2/TLR4	2
hsa-let-7b-5p	**C-type lectin receptor signaling pathway**	0.01	0.01	NRAS/AKT2	2
hsa-let-7b-5p	**T cell receptor signaling pathway**	0.01	0.01	NRAS/AKT2	2
hsa-let-7b-5p	**HIF-1 signaling pathway**	0.01	0.01	AKT2/TLR4	2
hsa-let-7b-5p	**Toxoplasmosis**	0.01	0.01	AKT2/TLR4	2

hsa-let-7b-5p	**Cholinergic synapse**	0.01	0.01	NRAS/AKT2	2
hsa-let-7b-5p	**Sphingolipid signaling pathway**	0.01	0.01	NRAS/AKT2	2
hsa-let-7b-5p	**Neurotrophin signaling pathway**	0.01	0.01	NRAS/AKT2	2
hsa-let-7b-5p	**Growth hormone synthesis, secretion and action**	0.01	0.01	NRAS/AKT2	2
hsa-let-7b-5p	**Relaxin signaling pathway**	0.01	0.01	NRAS/AKT2	2
hsa-let-7b-5p	**Apoptosis**	0.02	0.01	NRAS/AKT2	2
hsa-let-7b-5p	**Insulin signaling pathway**	0.02	0.01	NRAS/AKT2	2
hsa-let-7b-5p	**Yersinia infection**	0.02	0.01	AKT2/TLR4	2
hsa-let-7b-5p	**Estrogen signaling pathway**	0.02	0.01	NRAS/AKT2	2
hsa-let-7b-5p	**Autophagy - animal**	0.02	0.01	NRAS/AKT2	2
hsa-let-7b-5p	**Signaling pathways regulating pluripotency of stem cells**	0.02	0.01	NRAS/AKT2	2
hsa-let-7b-5p	**Phospholipase D signaling pathway**	0.02	0.01	NRAS/AKT2	2
hsa-let-7b-5p	**Oxytocin signaling pathway**	0.02	0.02	CCND1/NRAS	2
hsa-let-7b-5p	**mTOR signaling pathway**	0.02	0.02	NRAS/AKT2	2
hsa-let-7b-5p	**Hippo signaling pathway**	0.02	0.02	CCND1/CCND2	2
hsa-let-7b-5p	**Wnt signaling pathway**	0.02	0.02	CCND1/CCND2	2
hsa-let-7b-5p	**Tuberculosis**	0.03	0.02	AKT2/TLR4	2
hsa-let-7b-5p	**Neutrophil extracellular trap formation**	0.03	0.02	AKT2/TLR4	2
hsa-let-7b-5p	**Chemokine signaling pathway**	0.03	0.02	NRAS/AKT2	2
hsa-let-7b-5p	**Transcriptional misregulation in cancer**	0.03	0.02	CCND2/CCNA1	2
hsa-let-7b-5p	**Rap1 signaling pathway**	0.04	0.03	NRAS/AKT2	2
hsa-let-7b-5p	**Chemical carcinogenesis - reactive oxygen species**	0.04	0.03	NRAS/AKT2	2
hsa-let-7b-5p	**Pentose phosphate pathway**	0.04	0.03	RPIA	1
hsa-let-7b-5p	**Ras signaling pathway**	0.04	0.03	NRAS/AKT2	2
hsa-let-7b-5p	**Shigellosis**	0.05	0.03	AKT2/TLR4	2
hsa-let-7b-5p	**Salmonella infection**	0.05	0.03	AKT2/TLR4	2
hsa-let-7b-5p	**Carbohydrate digestion and absorption**	0.07	0.04	AKT2	1
hsa-let-7b-5p	**MAPK signaling pathway**	0.07	0.04	NRAS/AKT2	2
hsa-let-7b-5p	**Malaria**	0.07	0.04	TLR4	1
hsa-let-7b-5p	**Regulation of lipolysis in adipocytes**	0.08	0.05	AKT2	1
hsa-let-7b-5p	**Legionellosis**	0.08	0.05	TLR4	1
hsa-let-7b-5p	**Long-term depression**	0.09	0.05	NRAS	1
hsa-let-7b-5p	**Viral myocarditis**	0.09	0.05	CCND1	1
hsa-let-7b-5p	**Lysine degradation**	0.09	0.05	EZH2	1

hsa-let-7b-5p	**Inflammatory bowel disease**	0.09	0.05	TLR4	1
hsa-let-7b-5p	**Long-term potentiation**	0.09	0.05	NRAS	1
hsa-let-7b-5p	**Retinol metabolism**	0.10	0.05	RDH10	1
hsa-let-7b-5p	**Adipocytokine signaling pathway**	0.10	0.05	AKT2	1
hsa-miR-124-3p	**AGE-RAGE signaling pathway in diabetic complications**	0.00	0.00	MAPK14/CDK4/NFATC1/CCL2/PIK3CA/AKT2/STAT3/RAC1/PIM1	9
hsa-miR-124-3p	**Cellular senescence**	0.00	0.00	MAPK14/CDK4/CDK6/NFATC1/CDK2/PIK3CA/AKT2/SIRT1/CCND2	9
hsa-miR-124-3p	**Human cytomegalovirus infection**	0.00	0.00	MAPK14/CDK4/CDK6/NFATC1/CCL2/IL6R/PIK3CA/AKT2/STAT3/RAC1	10
hsa-miR-124-3p	**Epstein-Barr virus infection**	0.00	0.00	MAPK14/CDK4/CDK6/CDK2/PIK3CA/AKT2/STAT3/RAC1/CCND2	9
hsa-miR-124-3p	**Pancreatic cancer**	0.00	0.00	CDK4/CDK6/PIK3CA/AKT2/STAT3/RAC1	6
hsa-miR-124-3p	**Kaposi sarcoma-associated herpesvirus infection**	0.00	0.00	MAPK14/CDK4/CDK6/NFATC1/PIK3CA/AKT2/STAT3/RAC1	8
hsa-miR-124-3p	**FoxO signaling pathway**	0.00	0.00	MAPK14/CDK2/PIK3CA/AKT2/SIRT1/STAT3/CCND2	7
hsa-miR-124-3p	**Yersinia infection**	0.00	0.00	MAPK14/NFATC1/RHOG/CCL2/PIK3CA/AKT2/RAC1	7
hsa-miR-124-3p	**Measles**	0.00	0.00	CDK4/CDK6/CDK2/PIK3CA/AKT2/STAT3/CCND2	7
hsa-miR-124-3p	**Non-alcoholic fatty liver disease**	0.00	0.00	ADIPOR2/CEBPA/MAPK14/IL6R/PIK3CA/AKT2/RAC1	7
hsa-miR-124-3p	**PI3K-Akt signaling pathway**	0.00	0.00	BDNF/CDK4/CDK6/CDK2/IL6R/PIK3CA/AKT2/RAC1/CCND2	9
hsa-miR-124-3p	**Acute myeloid leukemia**	0.00	0.00	CEBPA/PIK3CA/AKT2/STAT3/PIM1	5
hsa-miR-124-3p	**Prolactin signaling pathway**	0.00	0.00	MAPK14/PIK3CA/AKT2/STAT3/CCND2	5
hsa-miR-124-3p	**Non-small cell lung cancer**	0.00	0.00	CDK4/CDK6/PIK3CA/AKT2/STAT3	5
hsa-miR-124-3p	**Chronic myeloid leukemia**	0.00	0.00	CDK4/CDK6/PIK3CA/AKT2/CBL	5
hsa-miR-124-3p	**Viral carcinogenesis**	0.00	0.00	CDK4/CDK6/CDK2/PIK3CA/STAT3/RAC1/CCND2	7
hsa-miR-124-3p	**Lipid and atherosclerosis**	0.00	0.00	MAPK14/NFATC1/CCL2/PIK3CA/AKT2/STAT3/RAC1	7
hsa-miR-124-3p	**PD-L1 expression and PD-1 checkpoint pathway in cancer**	0.00	0.00	MAPK14/NFATC1/PIK3CA/AKT2/STAT3	5
hsa-miR-124-3p	**Small cell lung cancer**	0.00	0.00	CDK4/CDK6/CDK2/PIK3CA/AKT2	5
hsa-miR-124-3p	**Hepatitis C**	0.00	0.00	CDK4/CDK6/CDK2/PIK3CA/AKT2/STAT3	6
hsa-miR-124-3p	**JAK-STAT signaling pathway**	0.00	0.00	IL6R/PIK3CA/AKT2/STAT3/CCND2/PIM1	6
hsa-miR-124-3p	**Hepatitis B**	0.00	0.00	MAPK14/NFATC1/CDK2/PIK3CA/AKT2/STAT3	6
hsa-miR-124-3p	**Endocrine resistance**	0.00	0.00	MAPK14/CDK4/PIK3CA/AKT2/JAG1	5
hsa-miR-124-3p	**T cell receptor signaling pathway**	0.00	0.00	MAPK14/CDK4/NFATC1/PIK3CA/AKT2	5
hsa-miR-124-3p	**Th17 cell differentiation**	0.00	0.00	MAPK14/AHR/NFATC1/IL6R/STAT3	5
hsa-miR-124-3p	**TNF signaling pathway**	0.00	0.00	MAPK14/CCL2/PIK3CA/AKT2/JAG1	5
hsa-miR-124-3p	**VEGF signaling pathway**	0.00	0.00	MAPK14/PIK3CA/AKT2/RAC1	4
hsa-miR-124-3p	**Sphingolipid signaling pathway**	0.00	0.00	MAPK14/PIK3CA/AKT2/SGPL1/RAC1	5

hsa-miR-124-3p	**Neurotrophin signaling pathway**	0.00	0.00	BDNF/MAPK14/PIK3CA/AKT2/RAC1	5
hsa-miR-124-3p	**Proteoglycans in cancer**	0.00	0.00	MAPK14/PIK3CA/AKT2/STAT3/RAC1/CBL	6
hsa-miR-124-3p	**Osteoclast differentiation**	0.00	0.00	MAPK14/NFATC1/PIK3CA/AKT2/RAC1	5
hsa-miR-124-3p	**Fc epsilon RI signaling pathway**	0.00	0.00	MAPK14/PIK3CA/AKT2/RAC1	4
hsa-miR-124-3p	**Chemical carcinogenesis - receptor activation**	0.00	0.00	AHR/AR/PIK3CA/AKT2/STAT3/JAG1	6
hsa-miR-124-3p	**MicroRNAs in cancer**	0.00	0.00	CDK6/EZH2/PIK3CA/SIRT1/STAT3/CCND2/PIM1	7
hsa-miR-124-3p	**Melanoma**	0.00	0.00	CDK4/CDK6/PIK3CA/AKT2	4
hsa-miR-124-3p	**p53 signaling pathway**	0.00	0.00	CDK4/CDK6/CDK2/CCND2	4
hsa-miR-124-3p	**Human T-cell leukemia virus 1 infection**	0.00	0.00	CDK4/NFATC1/CDK2/PIK3CA/AKT2/CCND2	6
hsa-miR-124-3p	**Fluid shear stress and atherosclerosis**	0.00	0.00	MAPK14/CCL2/PIK3CA/AKT2/RAC1	5
hsa-miR-124-3p	**Glioma**	0.00	0.00	CDK4/CDK6/PIK3CA/AKT2	4
hsa-miR-124-3p	**Bacterial invasion of epithelial cells**	0.00	0.00	RHOG/PIK3CA/RAC1/CBL	4
hsa-miR-124-3p	**Human papillomavirus infection**	0.00	0.00	CDK4/CDK6/CDK2/PIK3CA/AKT2/JAG1/CCND2	7
hsa-miR-124-3p	**Breast cancer**	0.00	0.00	CDK4/CDK6/PIK3CA/AKT2/JAG1	5
hsa-miR-124-3p	**EGFR tyrosine kinase inhibitor resistance**	0.00	0.00	IL6R/PIK3CA/AKT2/STAT3	4
hsa-miR-124-3p	**B cell receptor signaling pathway**	0.00	0.00	NFATC1/PIK3CA/AKT2/RAC1	4
hsa-miR-124-3p	**Longevity regulating pathway**	0.00	0.00	ADIPOR2/PIK3CA/AKT2/SIRT1	4
hsa-miR-124-3p	**Influenza A**	0.00	0.00	CDK4/CDK6/CCL2/PIK3CA/AKT2	5
hsa-miR-124-3p	**Fc gamma R-mediated phagocytosis**	0.00	0.00	MYO10/PIK3CA/AKT2/RAC1	4
hsa-miR-124-3p	**Prostate cancer**	0.00	0.00	CDK2/AR/PIK3CA/AKT2	4
hsa-miR-124-3p	**Progesterone-mediated oocyte maturation**	0.00	0.00	MAPK14/CDK2/PIK3CA/AKT2	4
hsa-miR-124-3p	**Chagas disease**	0.00	0.00	MAPK14/CCL2/PIK3CA/AKT2	4
hsa-miR-124-3p	**Toll-like receptor signaling pathway**	0.00	0.00	MAPK14/PIK3CA/AKT2/RAC1	4
hsa-miR-124-3p	**C-type lectin receptor signaling pathway**	0.00	0.00	MAPK14/NFATC1/PIK3CA/AKT2	4
hsa-miR-124-3p	**Chemokine signaling pathway**	0.00	0.00	CCL2/PIK3CA/AKT2/STAT3/RAC1	5
hsa-miR-124-3p	**HIF-1 signaling pathway**	0.00	0.00	IL6R/PIK3CA/AKT2/STAT3	4
hsa-miR-124-3p	**Growth hormone synthesis, secretion and action**	0.00	0.00	MAPK14/PIK3CA/AKT2/STAT3	4
hsa-miR-124-3p	**AMPK signaling pathway**	0.00	0.00	ADIPOR2/PIK3CA/AKT2/SIRT1	4
hsa-miR-124-3p	**Human immunodeficiency virus 1 infection**	0.00	0.00	MAPK14/NFATC1/PIK3CA/AKT2/RAC1	5
hsa-miR-124-3p	**Cell cycle**	0.00	0.00	CDK4/CDK6/CDK2/CCND2	4
hsa-miR-124-3p	**cAMP signaling pathway**	0.00	0.00	BDNF/NFATC1/PIK3CA/AKT2/RAC1	5
hsa-miR-124-3p	**Chemical carcinogenesis - reactive oxygen species**	0.00	0.00	MAPK14/AHR/PIK3CA/AKT2/RAC1	5
hsa-miR-124-3p	**Longevity regulating pathway - multiple species**	0.00	0.00	PIK3CA/AKT2/SIRT1	3
hsa-miR-124-3p	**Coronavirus disease - COVID-19**	0.00	0.00	MAPK14/CCL2/IL6R/PIK3CA/STAT3	5
hsa-miR-124-3p	**Alcoholic liver disease**	0.00	0.00	ADIPOR2/MAPK14/AKT2/SIRT1	4

hsa-miR-124-3p	**Signaling pathways regulating pluripotency of stem cells**	0.00	0.00	MAPK14/PIK3CA/AKT2/STAT3	4
hsa-miR-124-3p	**Adipocytokine signaling pathway**	0.00	0.00	ADIPOR2/AKT2/STAT3	3
hsa-miR-124-3p	**Renal cell carcinoma**	0.00	0.00	PIK3CA/AKT2/RAC1	3
hsa-miR-124-3p	**Salmonella infection**	0.00	0.00	MAPK14/RHOG/PIK3CA/AKT2/RAC1	5
hsa-miR-124-3p	**Cushing syndrome**	0.00	0.00	CDK4/CDK6/AHR/CDK2	4
hsa-miR-124-3p	**Hepatocellular carcinoma**	0.00	0.00	CDK4/CDK6/PIK3CA/AKT2	4
hsa-miR-124-3p	**ErbB signaling pathway**	0.00	0.00	PIK3CA/AKT2/CBL	3
hsa-miR-124-3p	**Colorectal cancer**	0.00	0.00	PIK3CA/AKT2/RAC1	3
hsa-miR-124-3p	**MAPK signaling pathway**	0.00	0.00	BDNF/MAPK14/NFATC1/AKT2/RAC1	5
hsa-miR-124-3p	**Th1 and Th2 cell differentiation**	0.01	0.01	MAPK14/NFATC1/JAG1	3
hsa-miR-124-3p	**Neutrophil extracellular trap formation**	0.01	0.01	MAPK14/PIK3CA/AKT2/RAC1	4
hsa-miR-124-3p	**Choline metabolism in cancer**	0.01	0.01	PIK3CA/AKT2/RAC1	3
hsa-miR-124-3p	**Focal adhesion**	0.01	0.01	PIK3CA/AKT2/RAC1/CCND2	4
hsa-miR-124-3p	**Diabetic cardiomyopathy**	0.01	0.01	MAPK14/PIK3CA/AKT2/RAC1	4
hsa-miR-124-3p	**Insulin resistance**	0.01	0.01	PIK3CA/AKT2/STAT3	3
hsa-miR-124-3p	**Rap1 signaling pathway**	0.01	0.01	MAPK14/PIK3CA/AKT2/RAC1	4
hsa-miR-124-3p	**Toxoplasmosis**	0.01	0.01	MAPK14/AKT2/STAT3	3
hsa-miR-124-3p	**Leukocyte transendothelial migration**	0.01	0.01	MAPK14/PIK3CA/RAC1	3
hsa-miR-124-3p	**Ras signaling pathway**	0.01	0.01	BDNF/PIK3CA/AKT2/RAC1	4
hsa-miR-124-3p	**Platelet activation**	0.01	0.01	MAPK14/PIK3CA/AKT2	3
hsa-miR-124-3p	**Relaxin signaling pathway**	0.01	0.01	MAPK14/PIK3CA/AKT2	3
hsa-miR-124-3p	**Oocyte meiosis**	0.01	0.01	MAPK14/CDK2/AR	3
hsa-miR-124-3p	**Natural killer cell mediated cytotoxicity**	0.01	0.01	NFATC1/PIK3CA/RAC1	3
hsa-miR-124-3p	**Carbohydrate digestion and absorption**	0.01	0.01	PIK3CA/AKT2	2
hsa-miR-124-3p	**Shigellosis**	0.01	0.01	MAPK14/PIK3CA/AKT2/RAC1	4
hsa-miR-124-3p	**Insulin signaling pathway**	0.02	0.01	PIK3CA/AKT2/CBL	3
hsa-miR-124-3p	**Gastric cancer**	0.02	0.02	CDK2/PIK3CA/AKT2	3
hsa-miR-124-3p	**Regulation of lipolysis in adipocytes**	0.02	0.02	PIK3CA/AKT2	2
hsa-miR-124-3p	**Endometrial cancer**	0.02	0.02	PIK3CA/AKT2	2
hsa-miR-124-3p	**GnRH secretion**	0.02	0.02	PIK3CA/AKT2	2
hsa-miR-124-3p	**Wnt signaling pathway**	0.02	0.02	NFATC1/RAC1/CCND2	3
hsa-miR-124-3p	**Inflammatory bowel disease**	0.03	0.02	NFATC1/STAT3	2
hsa-miR-124-3p	**Epithelial cell signaling in Helicobacter pylori infection**	0.03	0.02	MAPK14/RAC1	2
hsa-miR-124-3p	**Central carbon metabolism in cancer**	0.03	0.02	PIK3CA/AKT2	2

hsa-miR-124-3p	**Adherens junction**	0.03	0.02	SNAI2/RAC1	2
hsa-miR-124-3p	**Platinum drug resistance**	0.03	0.02	PIK3CA/AKT2	2
hsa-miR-124-3p	**Pathogenic Escherichia coli infection**	0.04	0.03	MAPK14/MYO10/RAC1	3
hsa-miR-124-3p	**IL-17 signaling pathway**	0.05	0.04	MAPK14/CCL2	2
hsa-miR-124-3p	**Glycosaminoglycan biosynthesis - keratan sulfate**	0.05	0.04	B4GALT1	1
hsa-miR-124-3p	**Inflammatory mediator regulation of TRP channels**	0.05	0.04	MAPK14/PIK3CA	2
hsa-miR-124-3p	**Viral protein interaction with cytokine and cytokine receptor**	0.06	0.04	CCL2/IL6R	2
hsa-miR-124-3p	**Glucagon signaling pathway**	0.06	0.04	AKT2/SIRT1	2
hsa-miR-124-3p	**Cholinergic synapse**	0.07	0.05	PIK3CA/AKT2	2
hsa-miR-124-3p	**Thyroid hormone signaling pathway**	0.08	0.05	PIK3CA/AKT2	2
hsa-miR-1-3p	**AGE-RAGE signaling pathway in diabetic complications**	0.00	0.00	PIM1/EDN1/CCND1/CCL2/CDK4/PIK3CA/KRAS	7
hsa-miR-1-3p	**Acute myeloid leukemia**	0.00	0.00	CEBPA/PIM1/CCND1/PIK3CA/KRAS	5
hsa-miR-1-3p	**Endocrine resistance**	0.00	0.00	CCND1/CDK4/SP1/PIK3CA/KRAS	5
hsa-miR-1-3p	**Human cytomegalovirus infection**	0.00	0.00	CCND1/CCL2/CDK4/SP1/PIK3CA/KRAS	6
hsa-miR-1-3p	**Melanoma**	0.00	0.00	CCND1/CDK4/PIK3CA/KRAS	4
hsa-miR-1-3p	**Non-small cell lung cancer**	0.00	0.00	CCND1/CDK4/PIK3CA/KRAS	4
hsa-miR-1-3p	**Breast cancer**	0.00	0.00	CCND1/CDK4/SP1/PIK3CA/KRAS	5
hsa-miR-1-3p	**Glioma**	0.00	0.00	CCND1/CDK4/PIK3CA/KRAS	4
hsa-miR-1-3p	**Pancreatic cancer**	0.00	0.00	CCND1/CDK4/PIK3CA/KRAS	4
hsa-miR-1-3p	**Chronic myeloid leukemia**	0.00	0.00	CCND1/CDK4/PIK3CA/KRAS	4
hsa-miR-1-3p	**Cellular senescence**	0.00	0.00	CCND1/CDK4/ETS1/PIK3CA/KRAS	5
hsa-miR-1-3p	**Hepatitis C**	0.00	0.00	YWHAZ/CCND1/CDK4/PIK3CA/KRAS	5
hsa-miR-1-3p	**Bladder cancer**	0.00	0.00	CCND1/CDK4/KRAS	3
hsa-miR-1-3p	**Viral carcinogenesis**	0.00	0.00	YWHAZ/CCND1/CDK4/PIK3CA/KRAS	5
hsa-miR-1-3p	**cAMP signaling pathway**	0.00	0.00	HCN2/BDNF/EDN1/SOX9/PIK3CA	5
hsa-miR-1-3p	**Human T-cell leukemia virus 1 infection**	0.00	0.00	CCND1/CDK4/ETS1/PIK3CA/KRAS	5
hsa-miR-1-3p	**PI3K-Akt signaling pathway**	0.00	0.00	BDNF/YWHAZ/CCND1/CDK4/PIK3CA/KRAS	6
hsa-miR-1-3p	**Endometrial cancer**	0.00	0.00	CCND1/PIK3CA/KRAS	3
hsa-miR-1-3p	**Fluid shear stress and atherosclerosis**	0.00	0.00	MEF2A/EDN1/CCL2/PIK3CA	4
hsa-miR-1-3p	**GnRH secretion**	0.00	0.00	HCN2/PIK3CA/KRAS	3
hsa-miR-1-3p	**Renal cell carcinoma**	0.00	0.00	ETS1/PIK3CA/KRAS	3
hsa-miR-1-3p	**Prolactin signaling pathway**	0.00	0.00	CCND1/PIK3CA/KRAS	3
hsa-miR-1-3p	**JAK-STAT signaling pathway**	0.00	0.00	PIM1/IL11/CCND1/PIK3CA	4

hsa-miR-1-3p	**Hepatocellular carcinoma**	0.00	0.00	CCND1/CDK4/PIK3CA/KRAS	4
hsa-miR-1-3p	**Colorectal cancer**	0.00	0.01	CCND1/PIK3CA/KRAS	3
hsa-miR-1-3p	**Kaposi sarcoma-associated herpesvirus infection**	0.00	0.01	CCND1/CDK4/PIK3CA/KRAS	4
hsa-miR-1-3p	**Small cell lung cancer**	0.00	0.01	CCND1/CDK4/PIK3CA	3
hsa-miR-1-3p	**Diabetic cardiomyopathy**	0.00	0.01	SP1/PIK3CA/ND1/COX1	4
hsa-miR-1-3p	**Prostate cancer**	0.00	0.01	CCND1/PIK3CA/KRAS	3
hsa-miR-1-3p	**Choline metabolism in cancer**	0.00	0.01	SP1/PIK3CA/KRAS	3
hsa-miR-1-3p	**T cell receptor signaling pathway**	0.00	0.01	CDK4/PIK3CA/KRAS	3
hsa-miR-1-3p	**Chemical carcinogenesis - reactive oxygen species**	0.00	0.01	PIK3CA/ND1/COX1/KRAS	4
hsa-miR-1-3p	**TNF signaling pathway**	0.00	0.01	EDN1/CCL2/PIK3CA	3
hsa-miR-1-3p	**Ras signaling pathway**	0.00	0.01	BDNF/ETS1/PIK3CA/KRAS	4
hsa-miR-1-3p	**Neurotrophin signaling pathway**	0.00	0.01	BDNF/PIK3CA/KRAS	3
hsa-miR-1-3p	**Thyroid hormone signaling pathway**	0.00	0.01	CCND1/PIK3CA/KRAS	3
hsa-miR-1-3p	**Aldosterone-regulated sodium reabsorption**	0.00	0.01	PIK3CA/KRAS	2
hsa-miR-1-3p	**Thyroid cancer**	0.00	0.01	CCND1/KRAS	2
hsa-miR-1-3p	**Cell cycle**	0.01	0.01	YWHAZ/CCND1/CDK4	3
hsa-miR-1-3p	**Relaxin signaling pathway**	0.01	0.01	EDN1/PIK3CA/KRAS	3
hsa-miR-1-3p	**FoxO signaling pathway**	0.01	0.01	CCND1/PIK3CA/KRAS	3
hsa-miR-1-3p	**Estrogen signaling pathway**	0.01	0.01	SP1/PIK3CA/KRAS	3
hsa-miR-1-3p	**Apelin signaling pathway**	0.01	0.01	MEF2A/CCND1/KRAS	3
hsa-miR-1-3p	**Measles**	0.01	0.01	CCND1/CDK4/PIK3CA	3
hsa-miR-1-3p	**Gastric cancer**	0.01	0.01	CCND1/PIK3CA/KRAS	3
hsa-miR-1-3p	**Non-alcoholic fatty liver disease**	0.01	0.01	CEBPA/PIK3CA/COX1	3
hsa-miR-1-3p	**Cushing syndrome**	0.01	0.01	CCND1/CDK4/SP1	3
hsa-miR-1-3p	**Hippo signaling pathway**	0.01	0.01	SNAI2/YWHAZ/CCND1	3
hsa-miR-1-3p	**Huntington disease**	0.01	0.02	BDNF/SP1/ND1/COX1	4
hsa-miR-1-3p	**Hepatitis B**	0.01	0.02	YWHAZ/PIK3CA/KRAS	3
hsa-miR-1-3p	**MicroRNAs in cancer**	0.01	0.02	PIM1/CCND1/PIK3CA/KRAS	4
hsa-miR-1-3p	**VEGF signaling pathway**	0.01	0.02	PIK3CA/KRAS	2
hsa-miR-1-3p	**Influenza A**	0.01	0.02	CCL2/CDK4/PIK3CA	3
hsa-miR-1-3p	**Human papillomavirus infection**	0.01	0.02	CCND1/CDK4/PIK3CA/KRAS	4
hsa-miR-1-3p	**Longevity regulating pathway - multiple species**	0.01	0.02	PIK3CA/KRAS	2
hsa-miR-1-3p	**Fc epsilon RI signaling pathway**	0.02	0.02	PIK3CA/KRAS	2
hsa-miR-1-3p	**Chemokine signaling pathway**	0.02	0.02	CCL2/PIK3CA/KRAS	3
hsa-miR-1-3p	**Transcriptional misregulation in cancer**	0.02	0.02	CEBPA/PAX3/SP1	3

hsa-miR-1-3p	**Central carbon metabolism in cancer**	0.02	0.02	PIK3CA/KRAS	2
hsa-miR-1-3p	**Mitophagy - animal**	0.02	0.02	SP1/KRAS	2
hsa-miR-1-3p	**p53 signaling pathway**	0.02	0.02	CCND1/CDK4	2
hsa-miR-1-3p	**Epstein-Barr virus infection**	0.02	0.02	CCND1/CDK4/PIK3CA	3
hsa-miR-1-3p	**Proteoglycans in cancer**	0.02	0.02	CCND1/PIK3CA/KRAS	3
hsa-miR-1-3p	**EGFR tyrosine kinase inhibitor resistance**	0.02	0.02	PIK3CA/KRAS	2
hsa-miR-1-3p	**Chemical carcinogenesis - receptor activation**	0.02	0.02	CCND1/PIK3CA/KRAS	3
hsa-miR-1-3p	**Alzheimer disease**	0.02	0.02	PIK3CA/ND1/COX1/KRAS	4
hsa-miR-1-3p	**Lipid and atherosclerosis**	0.02	0.02	CCL2/PIK3CA/KRAS	3
hsa-miR-1-3p	**B cell receptor signaling pathway**	0.02	0.02	PIK3CA/KRAS	2
hsa-miR-1-3p	**ErbB signaling pathway**	0.02	0.03	PIK3CA/KRAS	2
hsa-miR-1-3p	**Cardiac muscle contraction**	0.02	0.03	ASPH/COX1	2
hsa-miR-1-3p	**Longevity regulating pathway**	0.03	0.03	PIK3CA/KRAS	2
hsa-miR-1-3p	**PD-L1 expression and PD-1 checkpoint pathway in cancer**	0.03	0.03	PIK3CA/KRAS	2
hsa-miR-1-3p	**Thermogenesis**	0.03	0.03	ND1/COX1/KRAS	3
hsa-miR-1-3p	**Rheumatoid arthritis**	0.03	0.03	IL11/CCL2	2
hsa-miR-1-3p	**Melanogenesis**	0.03	0.03	EDN1/KRAS	2
hsa-miR-1-3p	**Progesterone-mediated oocyte maturation**	0.03	0.03	PIK3CA/KRAS	2
hsa-miR-1-3p	**Chagas disease**	0.03	0.03	CCL2/PIK3CA	2
hsa-miR-1-3p	**C-type lectin receptor signaling pathway**	0.03	0.03	PIK3CA/KRAS	2
hsa-miR-1-3p	**Parathyroid hormone synthesis, secretion and action**	0.04	0.03	MEF2A/SP1	2
hsa-miR-1-3p	**HIF-1 signaling pathway**	0.04	0.04	EDN1/PIK3CA	2
hsa-miR-1-3p	**Cholinergic synapse**	0.04	0.04	PIK3CA/KRAS	2
hsa-miR-1-3p	**Prion disease**	0.04	0.04	PIK3CA/ND1/COX1	3
hsa-miR-1-3p	**Pathways of neurodegeneration - multiple diseases**	0.04	0.04	BDNF/ND1/COX1/KRAS	4
hsa-miR-1-3p	**Sphingolipid signaling pathway**	0.04	0.04	PIK3CA/KRAS	2
hsa-miR-1-3p	**Growth hormone synthesis, secretion and action**	0.04	0.04	PIK3CA/KRAS	2
hsa-miR-1-3p	**AMPK signaling pathway**	0.04	0.04	CCND1/PIK3CA	2
hsa-miR-1-3p	**Natural killer cell mediated cytotoxicity**	0.05	0.05	PIK3CA/KRAS	2
hsa-miR-1-3p	**Oxidative phosphorylation**	0.05	0.05	ND1/COX1	2
hsa-miR-1-3p	**Apoptosis**	0.06	0.05	PIK3CA/KRAS	2
hsa-miR-1-3p	**Insulin signaling pathway**	0.06	0.05	PIK3CA/KRAS	2
hsa-miR-1-3p	**Yersinia infection**	0.06	0.05	CCL2/PIK3CA	2
hsa-miR-1-3p	**Autophagy - animal**	0.06	0.05	PIK3CA/KRAS	2

hsa-miR-1-3p	**Signaling pathways regulating pluripotency of stem cells**	0.06	0.05	PIK3CA/KRAS	2
hsa-miR-1-3p	**Spinocerebellar ataxia**	0.06	0.05	SP1/PIK3CA	2
hsa-miR-1-3p	**Phospholipase D signaling pathway**	0.07	0.05	PIK3CA/KRAS	2
hsa-miR-1-3p	**Oxytocin signaling pathway**	0.07	0.05	CCND1/KRAS	2
hsa-miR-1-3p	**mTOR signaling pathway**	0.07	0.05	PIK3CA/KRAS	2
hsa-miR-155-5p	**Kaposi sarcoma-associated herpesvirus infection**	0.00	0.00	IKBKE/KRAS/FADD/RAC1/CCND1/CXCL8/E2F2/MAPK14/MYC/HIF1A	10
hsa-miR-155-5p	**Hepatitis B**	0.00	0.00	TAB2/IKBKE/KRAS/FADD/YWHAZ/CXCL8/E2F2/MAPK14/MYC	9
hsa-miR-155-5p	**Colorectal cancer**	0.00	0.00	MSH6/MSH2/RHOA/KRAS/RAC1/CCND1/MYC	7
hsa-miR-155-5p	**AGE-RAGE signaling pathway in diabetic complications**	0.00	0.00	AGTR1/EDN1/KRAS/RAC1/CCND1/CXCL8/MAPK14	7
hsa-miR-155-5p	**Epstein-Barr virus infection**	0.00	0.00	TAB2/IKBKE/FADD/RAC1/CCND1/E2F2/MAPK14/MYC/CCND2	9
hsa-miR-155-5p	**Cellular senescence**	0.00	0.00	KRAS/ETS1/CCND1/CXCL8/E2F2/MAPK14/MYC/CCND2	8
hsa-miR-155-5p	**Human cytomegalovirus infection**	0.00	0.00	RHOA/KRAS/FADD/RAC1/CCND1/CXCL8/E2F2/MAPK14/MYC	9
hsa-miR-155-5p	**Bladder cancer**	0.00	0.00	KRAS/CCND1/CXCL8/E2F2/MYC	5
hsa-miR-155-5p	**Toll-like receptor signaling pathway**	0.00	0.00	TAB2/IKBKE/FADD/RAC1/CXCL8/MAPK14	6
hsa-miR-155-5p	**Hepatitis C**	0.00	0.00	IKBKE/KRAS/FADD/YWHAZ/CCND1/E2F2/MYC	7
hsa-miR-155-5p	**Prolactin signaling pathway**	0.00	0.00	SOCS1/KRAS/CCND1/MAPK14/CCND2	5
hsa-miR-155-5p	**Salmonella infection**	0.00	0.00	TAB2/RHOA/FADD/RAC1/ANXA2/CXCL8/MAPK14/MYC	8
hsa-miR-155-5p	**Pancreatic cancer**	0.00	0.00	KRAS/RAC1/CCND1/E2F2/RAD51	5
hsa-miR-155-5p	**Cell cycle**	0.00	0.00	YWHAZ/WEE1/CCND1/E2F2/MYC/CCND2	6
hsa-miR-155-5p	**Pathogenic Escherichia coli infection**	0.00	0.00	TAB2/MYO10/RHOA/FADD/RAC1/CXCL8/MAPK14	7
hsa-miR-155-5p	**Proteoglycans in cancer**	0.00	0.00	RHOA/KRAS/RAC1/CCND1/MAPK14/MYC/HIF1A	7
hsa-miR-155-5p	**Alcoholic liver disease**	0.00	0.00	TAB2/IKBKE/FADD/CCND1/CXCL8/MAPK14	6
hsa-miR-155-5p	**Lipid and atherosclerosis**	0.00	0.00	TAB2/RHOA/IKBKE/KRAS/RAC1/CXCL8/MAPK14	7
hsa-miR-155-5p	**IL-17 signaling pathway**	0.00	0.00	TAB2/IKBKE/FADD/CXCL8/MAPK14	5
hsa-miR-155-5p	**Hepatocellular carcinoma**	0.00	0.00	ARID2/KRAS/CCND1/SMARCA4/E2F2/MYC	6
hsa-miR-155-5p	**NOD-like receptor signaling pathway**	0.00	0.00	TAB2/RHOA/IKBKE/FADD/CXCL8/MAPK14	6
hsa-miR-155-5p	**Renal cell carcinoma**	0.00	0.00	KRAS/ETS1/RAC1/HIF1A	4
hsa-miR-155-5p	**Transcriptional misregulation in cancer**	0.00	0.00	RUNX2/FLI1/BCL6/CXCL8/MYC/CCND2	6
hsa-miR-155-5p	**RIG-I-like receptor signaling pathway**	0.00	0.00	IKBKE/FADD/CXCL8/MAPK14	4
hsa-miR-155-5p	**FoxO signaling pathway**	0.00	0.00	KRAS/BCL6/CCND1/MAPK14/CCND2	5
hsa-miR-155-5p	**Viral carcinogenesis**	0.00	0.00	RHOA/KRAS/YWHAZ/RAC1/CCND1/CCND2	6
hsa-miR-155-5p	**Chronic myeloid leukemia**	0.00	0.00	KRAS/CCND1/E2F2/MYC	4

hsa-miR-155-5p	**Yersinia infection**	0.00	0.00	TAB2/RHOA/RAC1/CXCL8/MAPK14	5
hsa-miR-155-5p	**Apelin signaling pathway**	0.00	0.00	AGTR1/KRAS/MEF2A/CCND1/TFAM	5
hsa-miR-155-5p	**Measles**	0.00	0.00	TAB2/IKBKE/FADD/CCND1/CCND2	5
hsa-miR-155-5p	**Fluid shear stress and atherosclerosis**	0.00	0.00	RHOA/EDN1/MEF2A/RAC1/MAPK14	5
hsa-miR-155-5p	**Human immunodeficiency virus 1 infection**	0.00	0.00	TAB2/KRAS/FADD/RAC1/WEE1/MAPK14	6
hsa-miR-155-5p	**Thyroid cancer**	0.00	0.00	KRAS/CCND1/MYC	3
hsa-miR-155-5p	**Human T-cell leukemia virus 1 infection**	0.00	0.00	KRAS/ETS1/CCND1/E2F2/MYC/CCND2	6
hsa-miR-155-5p	**MicroRNAs in cancer**	0.00	0.00	SOCS1/RHOA/KRAS/CCND1/E2F2/MYC/CCND2	7
hsa-miR-155-5p	**Endocrine resistance**	0.00	0.00	KRAS/CCND1/E2F2/MAPK14	4
hsa-miR-155-5p	**Wnt signaling pathway**	0.00	0.00	RHOA/RAC1/CCND1/MYC/CCND2	5
hsa-miR-155-5p	**C-type lectin receptor signaling pathway**	0.00	0.01	RHOA/IKBKE/KRAS/MAPK14	4
hsa-miR-155-5p	**TNF signaling pathway**	0.00	0.01	TAB2/EDN1/FADD/MAPK14	4
hsa-miR-155-5p	**Endometrial cancer**	0.00	0.01	KRAS/CCND1/MYC	3
hsa-miR-155-5p	**Sphingolipid signaling pathway**	0.00	0.01	RHOA/KRAS/RAC1/MAPK14	4
hsa-miR-155-5p	**Neurotrophin signaling pathway**	0.00	0.01	RHOA/KRAS/RAC1/MAPK14	4
hsa-miR-155-5p	**VEGF signaling pathway**	0.00	0.01	KRAS/RAC1/MAPK14	3
hsa-miR-155-5p	**Thyroid hormone signaling pathway**	0.00	0.01	KRAS/CCND1/MYC/HIF1A	4
hsa-miR-155-5p	**MAPK signaling pathway**	0.00	0.01	TAB2/KRAS/RAC1/RAPGEF2/MAPK14/MYC	6
hsa-miR-155-5p	**Osteoclast differentiation**	0.00	0.01	TAB2/SOCS1/RAC1/MAPK14	4
hsa-miR-155-5p	**Rap1 signaling pathway**	0.00	0.01	RHOA/KRAS/RAC1/RAPGEF2/MAPK14	5
hsa-miR-155-5p	**Chemical carcinogenesis - receptor activation**	0.01	0.01	IKBKE/KRAS/BCL6/CCND1/MYC	5
hsa-miR-155-5p	**Acute myeloid leukemia**	0.01	0.01	KRAS/CCND1/MYC	3
hsa-miR-155-5p	**Fc epsilon RI signaling pathway**	0.01	0.01	KRAS/RAC1/MAPK14	3
hsa-miR-155-5p	**Epithelial cell signaling in Helicobacter pylori infection**	0.01	0.01	RAC1/CXCL8/MAPK14	3
hsa-miR-155-5p	**Central carbon metabolism in cancer**	0.01	0.01	KRAS/MYC/HIF1A	3
hsa-miR-155-5p	**Melanoma**	0.01	0.01	KRAS/CCND1/E2F2	3
hsa-miR-155-5p	**Non-small cell lung cancer**	0.01	0.01	KRAS/CCND1/E2F2	3
hsa-miR-155-5p	**Mismatch repair**	0.01	0.01	MSH6/MSH2	2
hsa-miR-155-5p	**Platinum drug resistance**	0.01	0.01	MSH6/MSH2/FADD	3
hsa-miR-155-5p	**Signaling pathways regulating pluripotency of stem cells**	0.01	0.01	SMAD5/KRAS/MAPK14/MYC	4
hsa-miR-155-5p	**Glioma**	0.01	0.01	KRAS/CCND1/E2F2	3
hsa-miR-155-5p	**Coronavirus disease - COVID-19**	0.01	0.01	TAB2/AGTR1/IKBKE/CXCL8/MAPK14	5
hsa-miR-155-5p	**Breast cancer**	0.01	0.01	KRAS/CCND1/E2F2/MYC	4
hsa-miR-155-5p	**Pertussis**	0.01	0.01	RHOA/CXCL8/MAPK14	3

hsa-miR-155-5p	**Phospholipase D signaling pathway**	0.01	0.01	RHOA/AGTR1/KRAS/CXCL8	4
hsa-miR-155-5p	**Gastric cancer**	0.01	0.01	KRAS/CCND1/E2F2/MYC	4
hsa-miR-155-5p	**Hippo signaling pathway**	0.01	0.01	YWHAZ/CCND1/MYC/CCND2	4
hsa-miR-155-5p	**Shigellosis**	0.01	0.01	TAB2/RHOA/RAC1/CXCL8/MAPK14	5
hsa-miR-155-5p	**PI3K-Akt signaling pathway**	0.01	0.01	KRAS/YWHAZ/RAC1/CCND1/MYC/CCND2	6
hsa-miR-155-5p	**JAK-STAT signaling pathway**	0.01	0.01	SOCS1/CCND1/MYC/CCND2	4
hsa-miR-155-5p	**PD-L1 expression and PD-1 checkpoint pathway in cancer**	0.01	0.02	KRAS/MAPK14/HIF1A	3
hsa-miR-155-5p	**Tight junction**	0.01	0.02	RHOA/RAC1/RAPGEF2/CCND1	4
hsa-miR-155-5p	**Small cell lung cancer**	0.01	0.02	CCND1/E2F2/MYC	3
hsa-miR-155-5p	**TGF-beta signaling pathway**	0.01	0.02	SMAD5/RHOA/MYC	3
hsa-miR-155-5p	**Prostate cancer**	0.01	0.02	KRAS/CCND1/E2F2	3
hsa-miR-155-5p	**Choline metabolism in cancer**	0.01	0.02	KRAS/RAC1/HIF1A	3
hsa-miR-155-5p	**Chagas disease**	0.02	0.02	FADD/CXCL8/MAPK14	3
hsa-miR-155-5p	**T cell receptor signaling pathway**	0.02	0.02	RHOA/KRAS/MAPK14	3
hsa-miR-155-5p	**Chemokine signaling pathway**	0.02	0.02	RHOA/KRAS/RAC1/CXCL8	4
hsa-miR-155-5p	**Parathyroid hormone synthesis, secretion and action**	0.02	0.02	RHOA/RUNX2/MEF2A	3
hsa-miR-155-5p	**Toxoplasmosis**	0.02	0.02	TAB2/SOCS1/MAPK14	3
hsa-miR-155-5p	**Focal adhesion**	0.02	0.02	RHOA/RAC1/CCND1/CCND2	4
hsa-miR-155-5p	**Leukocyte transendothelial migration**	0.02	0.03	RHOA/RAC1/MAPK14	3
hsa-miR-155-5p	**Growth hormone synthesis, secretion and action**	0.02	0.03	SOCS1/KRAS/MAPK14	3
hsa-miR-155-5p	**Human papillomavirus infection**	0.03	0.03	IKBKE/KRAS/FADD/CCND1/CCND2	5
hsa-miR-155-5p	**Chemical carcinogenesis - reactive oxygen species**	0.03	0.03	KRAS/RAC1/MAPK14/HIF1A	4
hsa-miR-155-5p	**Relaxin signaling pathway**	0.03	0.03	EDN1/KRAS/MAPK14	3
hsa-miR-155-5p	**Vascular smooth muscle contraction**	0.03	0.04	RHOA/AGTR1/EDN1	3
hsa-miR-155-5p	**Ras signaling pathway**	0.03	0.04	RHOA/KRAS/ETS1/RAC1	4
hsa-miR-155-5p	**Hedgehog signaling pathway**	0.04	0.04	CCND1/CCND2	2
hsa-miR-155-5p	**Viral myocarditis**	0.04	0.04	RAC1/CCND1	2
hsa-miR-155-5p	**Oxytocin signaling pathway**	0.05	0.05	RHOA/KRAS/CCND1	3
hsa-miR-155-5p	**Non-alcoholic fatty liver disease**	0.05	0.05	RAC1/CXCL8/MAPK14	3
hsa-miR-155-5p	**Cushing syndrome**	0.05	0.05	AGTR1/CCND1/E2F2	3
hsa-miR-155-5p	**Renin secretion**	0.05	0.05	AGTR1/EDN1	2
hsa-miR-155-5p	**Adherens junction**	0.05	0.05	RHOA/RAC1	2
hsa-miR-155-5p	**Mitophagy - animal**	0.06	0.05	KRAS/HIF1A	2
hsa-miR-155-5p	**p53 signaling pathway**	0.06	0.05	CCND1/CCND2	2
